# A lightweight blockchain-inspired hybrid intrusion detection system with ensemble learning for tamper-proof auditing

**DOI:** 10.1371/journal.pone.0356878

**Published:** 2026-09-11

**Authors:** Shailendra Mishra, Reem Alshenaifi, Ruba Ahmed Alfahidah

**Affiliations:** 1 Department of Computer Engineering, College of Computer and Information Sciences, Majmaah University, Al Majmaah, Saudi Arabia; 2 Department of Information Technology, College of Computer and Information Sciences, Majmaah University, Al Majmaah, Saudi Arabia; Maulana Abul Kalam Azad University of Technology West Bengal, INDIA

## Abstract

The rapid expansion of digital systems has intensified the complexity of cyber threats, rendering traditional intrusion detection systems (IDS) inadequate against evolving attacks. This study proposes a hybrid IDS (H-IDS) that integrates supervised (SVM, Random Forest, CatBoost, DNN) and unsupervised (Isolation Forest, One-Class SVM, Autoencoder) models within an ensemble framework. Preprocessing employs PCA for dimensionality reduction and SMOTE for class balancing, while weighted voting based on cross-validation F1-scores optimizes ensemble decisions. A lightweight blockchain-inspired hash-chained audit log provides tamper-evident logging of detection events in a single-node deployment without decentralized consensus. Evaluated on NSL-KDD and CIC-IDS2017 datasets, H-IDS achieves 98.85% accuracy (pre-blockchain) and 98.15% (post-blockchain). The ledger operates in a private, single-node setting and introduces minimal local logging overhead and observed reductions in false positives (paired t-test, n = 3, p < 0.05). This work advances trustworthy, auditable, and high-performance intrusion detection for modern network environments.

## Introduction

The recent surge in digital interconnectivity and the Internet of Things (IoT) has dramatically increased the number of potential targets for cyberattacks, including critical infrastructures, power grids, healthcare systems, and transportation networks [1]. Traditional Intrusion Detection Systems (IDS), which rely on signature-based or statistical methods, are only as effective as their knowledge of existing threats and remain ineffective against zero-day exploits, high false alarm rates, and adaptive adversaries [2,3]. Existing frameworks either overfit to label training data or struggle with the imprecision of unsupervised models [4,5], and few approaches combine the two preprocessing techniques, such as principal component analysis (PCA) and synthetic minority over-sampling technique (SMOTE). Cross-dataset generalization is essentially virtually absent. There is no foolproof way to verify the accuracy of detection logs, which can be altered [6–8].

Blockchain technology is sometimes used in IDS, but it isn't fused with the full-scale hybrid learning [9–11]. As a consequence, the issue of trust, auditability, and forensic reliability is not addressed. Regarding the architecture of the unified Intrusion Detection System, the use of supervised and unsupervised ensemble techniques along with adaptive pre-processing and the use of lightweight blockchain is the key to effective detection, verification, and accountability [2,3,12,13]. This ensures the rapid and accurate detection of attacks while minimizing false alarm rates. The issue of vastly different and vastly imbalanced datasets [4,5,10].

This study proposed a Hybrid Intrusion Detection System (H-IDS), based on supervised and unsupervised models, cleans data with PCA and SMOTE, and logs every alert on a lightweight, blockchain-inspired, immutable audit ledger designed for tamper-evident logging in controlled environments.

By integrating advanced detection mechanisms, intelligent preprocessing, and decentralized validation, this study proposed a scalable, interpretable, and trustworthy framework for intrusion detection in modern dynamic threat landscapes.

### Key gaps in current IDS

While prior studies have effectively integrated machine learning ensembles with blockchain for enhanced intrusion detection, particularly in distributed, IoT, or vehicular environments, the proposed H-IDS advances the field through several targeted integrations: (1) a full-spectrum hybrid ensemble of seven diverse base learners with adaptive weighted voting derived from 5-fold cross-validation F1-scores; (2) a strictly leakage-proof preprocessing pipeline; (3) a lightweight single-node hash-chained audit log inspired auditing layer with quantified minimal overhead.

### Research objectives

- Fuse supervised and unsupervised models for comprehensive detection.
- Optimize via SMOTE, PCA, and ensemble weighting.
- Evaluate pre/post-Blockchain-inspired immutable ledger on CICIDS2017 and NSL-KDD.
- Embed transparent, tamper-resistant logging.
- Balance security and computational cost.

### Contribution

Proposed H-IDS delivers all four pillars in one system:

1. Hybrid ensemble (7 models, both paradigms) (Table 6, Fig 8).
2. Adaptive weighted voting from cross-validation (Table 7).
3. PCA + SMOTE preprocessing before dual pipelines (Fig 2, Table 2, ablation study in Table 9, Fig 6).
4. Lightweight immutable audit logging (Table 6, Table 7, Table 8, Fig 8, Fig 9)

While prior studies have explored hybrid intrusion detection models and blockchain-assisted logging independently, fewer works have examined a tightly integrated, lightweight hash-chained audit mechanism combined with supervised–unsupervised ensemble learning under a unified evaluation framework.

## Literature review

Traditional supervised methods are discussed in [2], SVM, RF, KNN, and DT, on the NSL-KDD and CICIDS2017 datasets, providing a comprehensive overview of the trade-off between accuracy and scalability. Saheed et al., [3] achieved an impressive 98.5% accuracy score with RF and XGBoost on IoT-BoT, confirmed with a t-test that showed p < 0.01. Imrana et al. [14], proposed BiDLSTM, and it's proven to be a top performer in the case of rare R2L/U2R attacks on NSL-KDD, hitting a spot-on 98.2% accuracy and 97.8% recall, supported by ANOVA significance, p < 0.05.

### Unsupervised machine learning

Unsupervised methods don't require labelled data; it work on anomalies in the data that are not previously known. Verkerken et al. (2022) [4], tested Isolation Forest, One-Class SVM, and Autoencoders on artificial data, getting a recall rate of 85% and a p-value of less than.01. Vaiyapuri and Binbusayyis (2020) [15] presented Deep Autoencoders for the NSL-KDD and CICIDS2017 datasets, resulting in accuracy levels of 92.1% and recall levels of 90.5%. Suresh et al., (2024) [16], proposed REL-BC, a hybrid autoencoder-ensemble model suitable for high-dimensional data, achieving 96.7% accuracy.

### Deep learning

Bamber et al. (2025) [5], proposed a combination of CNN and LSTM hybrid system, achieved an accuracy of 95.0% and an F1 score of 94.0 on the NSL-KDD dataset, and a t-test (p < 0.01) confirmed the significance of this result. Asgharzadeh et al. (2024) [17], presented a combination of FECNNIoT, BMEGTO and KNN that produced a staggering 99.85 to 99.99% accuracy on the NSL-KDD and TON-IoT datasets. Wilcoxon test with a p-value of less than 0.001confirmed the significance of this result. Yang et al. (2024) [18] proposed Inception with hybrid sampling to exceed the baseline on the CICIDS 2017 and Edge-IoT datasets.

### Ensemble methods (supervised & unsupervised mix)

Ensemble approach enhance reliability by integrating multiple models Shin et al. (2024) [7], pioneered the use of hybrid sampling and ensembles on the IoTID20 and CHADC2020 datasets, scores reached of over 98% with a statistical significance confirmed by a t-test, p < .05. Farooqi et al. (2023) [19] proposed DRX, a combination of Decision Trees, Random Forest and XGBoost that has proved to be very accurate on the NSL-KDD and CIC-IDS2017 datasets. This was confirmed by a t-test, p < .001, results are aligned with Lazzarini et al. (2023) [12] and Urmi et al. 2024 [20]. Alotaibi & Ilyas (2023) [13] used stacking/voting ensembles, achieving accuracy up to 98.63% on TON-IoT dataset. Bamber et al. (2025) further validated CNN-LSTM hybrids at 95% accuracy and 94% F1 on NSL-KDD [5].

### Blockchain integration with full ensemble

Blockchain-ensemble synergies IDS integrity. Babu et al. [11] integrated DT, RF, SVM, and LR with blockchain, detecting DDoS at 97.39% on CICDDoS2019. Mishra (2023) combined RF and SVM with blockchain on KDD99 and UNSW-NB15, reaching 99.95% accuracy [10]. Almalki et al. (2024) secured federated healthcare IoT using blockchain-enhanced intrusion detection [21]. Mohan et al. (2023) integrated Isolation Forest, K-Means, and LSTM with blockchain for transaction anomaly monitoring [22]. Sorour et al. (2025) developed LSTM-JSO for privacy-preserving federated IoT detection (98.1% accuracy) [23].

Son et al. (2024) applied semi-supervised learning with blockchain in supply chain anomaly detection [24]. Lv and Ding (2024) proposed KCLSTM (K-means + CNN-LSTM), achieving 93.3% accuracy, 93.7% F1, and 93.2% DR on NSL-KDD, but without blockchain or unsupervised depth [25]. Urmi et al. (2024) built a stacked ensemble (RF, XGBoost, Extra-Trees, Logistic Regression) with RFE/MI/LFS feature selection, attaining 99.95–100% accuracy on CICIDS2017 and NSL-KDD, missing blockchain, SMOTE, and unsupervised components [20].

### Hybrid/advanced approaches

This hybridized technique effectively uses the combination of clustering, metaheuristics, and deep learning to ensure the detection of anomalies. Ant Colony Optimization, Random Forest, Isolation Forest, which resulted in an accuracy rate of around 98.3% with zero false alarms [26,27]. Mjahed et al. (2023) presented a hybridized intrusion detection system using Fuzzy C-Means with Back Propagation Neural Network, which resulted in 99.97% accuracy and 99.96% F1 score on the CICIDS2017 dataset [28]. Other notable intrusion detection systems using the hybridized technique are SAAE-DNN, E-Tree ensemble, bio-inspired optimization using the Sperm Whale Algorithm, etc.

In spite of the high accuracy rates achieved by the intrusion detection systems based on the hybridized technique, which includes the combination of machine learning and deep learning, the intrusion detection systems based on the hybridized technique achieve high accuracy rates. For example, CNN-LSTM-based intrusion detection systems achieve an accuracy rate of around 95% on the NSL-KDD dataset [5],Feature-Enhanced Convolutional Neural Network (FECNN) framework used for IoT intrusion detection, FECNN-IoT combined with BMEGTO and KNN reaching 99.85–99.99% [17], semi-supervised DAE-MLP frameworks with Ethereum blockchain integration attaining 96.5% [24], Attention-BiGRU-Inception hybrids exceeding 99% on CICIDS2017 and Edge-IIoTset datasets via advanced sampling techniques [18], and metaheuristic-optimized Fuzzy C-Means with BPNN enhancing feature selection [28], alongside other high-performing approaches reporting up to ~99.9% on NSL-KDD/CICIDS datasets [29,30–32]. Existing approaches remain limited by their reliance on benchmark datasets like NSL-KDD and CICIDS2017, lacking robust evaluation on real-world IoT traffic and demonstrating poor generalization across diverse, cross-domain attack scenarios. Furthermore, while blockchain-assisted IDS frameworks enhance data integrity and distributed trust [35–37], they frequently introduce substantial computational overhead, failing to adequately address scalability issues and latency demands essential for resource-constrained IoT devices. Specific limitation includes, the lacking of unsupervised components and ensemble diversity in CNN-LSTM; the absence of blockchain, no SMOTE, no cross dataset validation, no oversampling techniques, no PCA, and the variants of FECNN-IoT, which are of high performance; the absence of SMOTE, no ensembles, and no PCA in the variants of FECNN-IoT, which were excluded in the blockchain-based semi-supervised models; the absence of blockchain and unsupervised learning in the attention-based BiGRU-Inception; the partially included blockchain, and the absence of full ensemble in Fuzzy C-Means, BPNN, and metaheuristic optimization.

### Research gap

- Absence of full spectrum hybridization: Many models only consider the integration of supervised or unsupervised models but rarely both.
- Inadequate blockchain integration: Although some models [10,11,33] utilize blockchain to ensure distributed trust and/or data integrity, they often fail to integrate sophisticated ensemble methods or unsupervised models, which limits the overall flexibility and traceability of the models.
- Inadequate preprocessing: Although many top-performing models fail to consistently utilize PCA for dimensionality reduction or SMOTE for class imbalance,
- Inadequate cross-dataset generalization: Many models are only trained on a single dataset.
- Lack of transparency and auditability: Few systems provide tamper-proof, real-time logging essential for compliance and forensics.

## Proposed Work Fills the Gap

This study proposes a Lightweight Blockchain-Inspired Hybrid Intrusion Detection System (H-IDS) with Ensemble Learning, designed to provide tamper-proof auditing, which integrates the following components:

- CatBoost, Random Forest, SVM, and DNN – supervised models.
- Isolation Forest, One-Class SVM, and Autoencoder – unsupervised models.
- SMOTE for class imbalance and PCA for feature dimensionality reduction are examples of preprocessing techniques.

To enhance transparency and trust, the lightweight blockchain-inspired layer provides real-time, tamper-evident auditing of intrusion detection decisions.

Table 1 presents a comparative analysis of recent intrusion detection systems (IDS). Most machine learning–based approaches achieve high accuracy, often 98% or higher, with approximately 70% of studies employing statistical validation techniques such as t-tests, ANOVA, or Wilcoxon tests. Deep learning and ensemble methods frequently reach ≥99% accuracy; however, none of these studies fully integrate blockchain-based audit logging or explore unsupervised model depth. Significant challenges remain in real-world generalization, minimizing false positives, and enabling lightweight deployment for IoT applications.

**Table 1:** Thematic Comparative Analysis of Intrusion Detection Systems: Techniques, Datasets, and key contributions.

Theme	Reference	Key Techniques	Dataset	Main Contribution/ Accuracy Highlight
Supervised ML	[2] Abdallah & Otoom (2022)	SVM, RF, KNN, DT	NSL-KDD, CICIDS2017	Survey of supervised IDS techniques
	[3] Saheed et al. (2022)	RF, XGBoost, SVM	IoT-BoT	ML-based IoT attack detection
	[14] Imrana et al. (2021)	BiDLSTM	NSL-KDD	Superior R2L/U2R detection
	[34]Almutairi, N. et al. (2025)	Optimized XGBoost	NSL-KDD	High-accuracy detection (99.93%)
Unsupervised ML	[4] Verkerken et al. (2022)	Isolation Forest, One-Class SVM, Autoencoders	Custom synthetic	Generalization in unsupervised NIDS
	[15] Vaiyapuri & Binbusayyis (2020)	Deep Autoencoder	NSL-KDD, CICIDS2017	One-class anomaly detection
	[16] Suresh et al. (2024)	REL-BC (Autoencoder + ELM)	Custom large-scale	High-dimensional anomaly capture
Deep Learning	[5] Bamber et al. (2025)	CNN-LSTM	NSL-KDD	Hybrid DL for intrusion detection
	[17] Asgharzadeh et al. (2024)	FECNNIoT + BMEGTO + KNN	NSL-KDD, TON-IoT	High-accuracy IoT IDS
	[18] Yang et al. (2024)	Attention-BiGRU-Inception + Hybrid Sampling	CICIDS2017, Edge-IIoTset	Improved IIoT detection
Ensemble Methods	[7] Shin et al. (2024)	Hybrid sampling + Ensemble	IoTID20, CHADC2020	Unbalanced multiclass detection
	[12] Lazzarini et al. (2023)	Stacked DL models	ToN_IoT, CICIDS2017	Low FP in IoT
	[19] Farooqi et al. (2023)	DRX (DT + RF + XGBoost)	NSL-KDD, CICIDS2017	Ensemble voting for IoT
	[13] Alotaibi & Ilyas (2023)	Stacking/Voting	TON-IoT	IoT security enhancement
	[20] Urmi et al. (2024)	Stacked Ensemble + Feature Selection	CICIDS2017	High detection with RFE/MI/LFS
	[30]Arnob, A. K. B et al., (2025)	Confidence-based soft voting	CICIDS2017, NSL-KDD, CICIoT2023	Cross-dataset generalization; up to 99.99% accuracy
Blockchain Integration	[9] Mansour (2022)	Blockchain + Clustering	IIoT	Secure clustering-based IDS
	[10] Mishra (2023)	Blockchain + RF/SVM	KDD99, UNSW-NB15	Privacy-preserving hybrid IDS
	[23] Sorour et al. (2025)	LSTM-JSO + Federated Learning	IoT	Privacy-preserving federated IDS
	[21] Almalki et al. (2024)	Federated Learning + Blockchain	EdgeIIoTset, CICIoT2023	Healthcare 5.0 secure IDS
	[11] Babu et al. (2022)	DT/RF/SVM + Blockchain	CICDDoS2019	DDoS detection with blockchain
	[24] Son et al. (2024)	DAE + MLP + Ethereum	Custom supply chain	Blockchain anomaly detection
	[35]Nandanwar & Katarya (2025)	Hybrid blockchain framework	IoT	Securing IDS in distributed IoT networks
	Kumar, S., & Sharma, B. (2025).	Blockchain + Explainable AI	IoT	Tamper-proof logging and real-time threat detection
Hybrid/Advanced	[26] Lifandali et al. (2023)	ACO + RF + Isolation Forest	Custom	Feature selection + anomaly detection
	[22] Mohan et al. (2023)	IF, K-Means, LSTM + Blockchain	Blockchain tx	Comparative anomaly detection
	[36] Tang et al. (2020)	SAAE-DNN	NSL-KDD	Self-adaptive autoencoder
	[33] Aldaej et al. (2024)	E-Tree + RF + DNN	Bot-IoT, CICIDS2018	Ensemble for IoT-edge
	[27] Mhamdi & Isa (2024)	Autoencoder-RF + Blockchain	SDN	Hybrid AE-RF with blockchain
	[28] Mjahed et al. (2023)	Fuzzy C-Means + BPNN + Metaheuristics	CICIDS2017	Metaheuristic-optimized IDS
	[25] Lv & Ding (2024)	K-means + CNN-LSTM	Custom	Hybrid clustering-DL
	[37] Elsedimy & AboHashish (2025)	Fuzzy C-Means + Sperm Whale Algorithm	IoT	Bio-inspired IoT IDS

Although there has been progress made in intrusion detection systems (IDS), because of the high-accuracy machine learning, deep learning, ensemble, and blockchain-integrated approaches, and having achieved near-perfect scores in datasets such as NSL-KDD and CICIDS2017, several important gaps remain. In deployments in real time, the current models perform exceptionally, however, in constrained resource environments of the Internet of Things (IoT), the models still have difficulties in resource constrained environments.

As for the blockchain hybrids, there is greater and more decentralization along with added privacy; however, there is more added latency as well as more complexity added for integrations in the distributed network. Moreover, while accuracies approach 99.99% on standard datasets, generalization to zero-day attacks, highly imbalanced real-world traffic, and cross-dataset robustness is limited. There is a need for lightweight, explainable, and adaptive frameworks that combine the strengths of ensembles, deep learning, and blockchain while addressing privacy-preserving federated learning and quantum-resistant security for emerging 6G/IoT ecosystems.

The proposed system combines:

- Models: SVM, RF, XGBoost, CatBoost, LSTM, ANN (supervised); Isolation Forest, One-Class SVM, Autoencoder (unsupervised).
- Preprocessing: Min-max scaling, PCA (95% variance), SMOTE (1:1 ratio).
- Ensemble: Weighted voting; Weights: wi = F1_i_ / Σ F1_j,_wi, per CV fold.
- Blockchain-Enabled Auditing: Blockchain‑inspired immutable logging layer using SHA‑256 hashing and a smart‑contract‑style logger that records each detection event with timestamp, predicted attack class, model confidence score, and a hashed feature signature to ensure tamper‑proof auditing of intrusion detections.

### Research methods

This section describes the proposed end-to-end methodology, including data pre-processing, ensemble model construction, and secure audit logging. Each component is designed to prevent data leakage, improve predictive robustness, and ensure result traceability.

### Leakage-proof preprocessing pipeline

Ensuring a leakage-free preprocessing workflow is critical for the validity and reproducibility of intrusion detection results, particularly when high classification performance is reported. To eliminate any possibility of information leakage from the test data into the training process, all preprocessing steps in this study are applied strictly after an initial train–test split and are fitted exclusively on training data. Steps are Train–Test Split, Feature Scaling, Dimensionality Reduction (PCA), Class Imbalance Handling (SMOTE), Model Training and Evaluation.

Processing Steps are;

Perform initial train-test split (80/20 stratified for CIC-IDS2017 [38]; official Train + /Test+ for NSL-KDD [39]).

To eliminate any possibility of information leakage, all preprocessing, model selection, and weighting steps were confined strictly to the training data. The workflow proceeds as follows: (1) the dataset is first partitioned into fixed training and test sets; (2) PCA is fitted exclusively on the training set and then applied to the test set using the learned transformation; (3) SMOTE is applied only to the training data after PCA; (4) cross-validation, hyperparameter tuning, and ensemble weight estimation are performed solely within the training partition; (5) the held-out test set is accessed once for final performance evaluation. No stage of model fitting, weighting, or threshold selection uses information from the test data.

Further ensuring reproducibility and validation, the entire data preprocessing process, the implementation of the ensemble weighting technique, as well as the model training scripts, and the blockchain-inspired audit log have been released through the GitHub project page (https://github.com/rubaamff/Hybrid-IDS-Blockchain) and a DOI release on Zenodo (https://doi.org/10.5281/zenodo.18009378). All this enables replication of the preprocessing process and the experiments performed.

These are the general steps.

**Dataset Splitting**: A stratified split is applied to the CICIDS 2017 datasets to split them into a training set and a testing set. This is achieved through the use of the ‘train_test_split’ function in the scikit-learn library. This ensures the data is evenly representative of the classes.

**Feature Scaling:** The StandardScaler is used on the training data and subsequently on the training and test data. The StandardScaler is used to scale the feature so that the mean is zero and the variance is one, which ensures faster convergence in models based on distances and gradients.

**Dimensionality Reduction Using PCA:** Principal Component Analysis (PCA) is used for reducing the dimensionality of the data while retaining the important information. The PCA is configured with n_components = 0.95, which ensures that 95% of the cumulative variance is retained in the data.

This dimensionality reduction results in lower computational complexity, elimination of redundant and noisy features, and improved model efficiency and generalization performance.

**Handling Class Imbalance with SMOTE:** The Synthetic Minority Over-Sampling Technique (SMOTE) is applied to the training data after it has been processed with PCA to create more instances of the smaller attack classes. The leakage-resistant processing sequence is applicable to the actual application scenario.

This sequence is critical for valid performance metrics. The proposed preprocessing pipeline follows a strict leakage-resilient sequence: (data splitting → feature scaling → dimensionality reduction using PCA → class balancing with SMOTE). Minor attack samples were added to the training dataset through SMOTE, and no synthetic samples were created from the testing dataset after applying PCA and any other pre-processing methods. Performance metrics are valid if the sequence of operations is maintained, by applying SMOTE prior to splitting the data artificially inflates recall values, while removing SMOTE results in lower recall for the minor attack class compared to using SMOTE. Therefore, the proposed pre-processing pipeline follows a strict sequence in order to minimize leakage: data split and scale, then reduction and balance using PCA and SMOTE, respectively.

### Ensemble model integration

The proposed Hybrid IDS combines seven base models into a weighted soft-voting ensemble, leveraging complementary strengths of supervised and unsupervised techniques. All models are implemented using scikit-learn v1.5.3, CatBoost v1.2, and TensorFlow/Keras for the DNN component.

**Supervised models**:

- CatBoostClassifier (iterations = 500, depth = 6, learning_rate = 0.1)
- RandomForestClassifier (n_estimators = 200)
- SVC (kernel = ’rbf,’ C = 10, probability = True)
- Deep Neural Network (Keras Sequential: input → 128 → 64 → 32 → 1, ReLU activations, sigmoid output, Adam optimizer, binary cross-entropy loss, epochs = 50, batch_size = 256)

**Unsupervised Models** (anomaly scores inverted to attack probability):

- IsolationForest (contamination = 0.05)
- Autoencoder (symmetric encoder-decoder: input → 64 → 32 → 16 → bottleneck; MSE reconstruction loss)
- OneClassSVM (nu = 0.05, kernel = ’rbf’)

Each model outputs a probability score (supervised: attack probability; unsupervised: normalized anomaly score inverted to “attack likelihood”).

**Ensemble Fusion**: Cross-validation-weighted soft voting. Individual model weights

w_i_ are computed from macro F1-scores on a 10% validation hold-out:

**Weighting Formula**: Weights w _i_ are derived from individual model F1-scores on a validation hold-out set: w_i_  = F1i / Σ F1j. Final ensemble probability:


$$\begin{array}{c} P_{\text{ensemble}}=sum\;(i=1\;to\;7)\;wi\times Pi \end{array}$$

$$\begin{array}{c} w_{i}=F1i/sum(j=1\;to\;7)\;F1j \end{array}$$

The number 7 represents the total number of base models used in the ensemble.

Decision threshold = 0.5 (optimized for balanced F1; adjustable for low-FPR scenarios).

Ensemble weights were derived solely from macro-F1 scores obtained during cross-validation on the training data; no test-set information influenced weight assignment or decision thresholds.

**Key hyperparameters** (optimized via grid search in the repository implementation):

- CatBoost: depth = 6, learning_rate = 0.1, iterations = 500.
- RF: n_estimators = 200, max_depth = None.
- SVM: kernel = ’rbf,’ C = 10.
- DNN: 3 hidden layers (128-64-32 neurons), ReLU activation, Adam optimizer, epochs = 50, batch_size = 256.
- Isolation Forest: contamination = 0.05.
- Autoencoder: Encoder/decoder symmetric (input → 64 → 32 → 16 → bottleneck), MSE reconstruction loss.
- One-Class SVM: nu = 0.05, kernel = ’rbf.’

### Blockchain-inspired immutable audit logging

Each log entry (block) contains:

- Index
- Timestamp
- Detection metadata (prediction, probability, feature summary, IoC)
- Hash of previous block
- Current block hash (SHA-256 of concatenated fields)

Appending a new entry computes:

current_hash = hashlib.sha256 ((prev_hash + json_data + timestamp).encode ()).hexdigest()

This creates a tamper-evident chain: any modification invalidates subsequent hashes.

**Fig 1:**
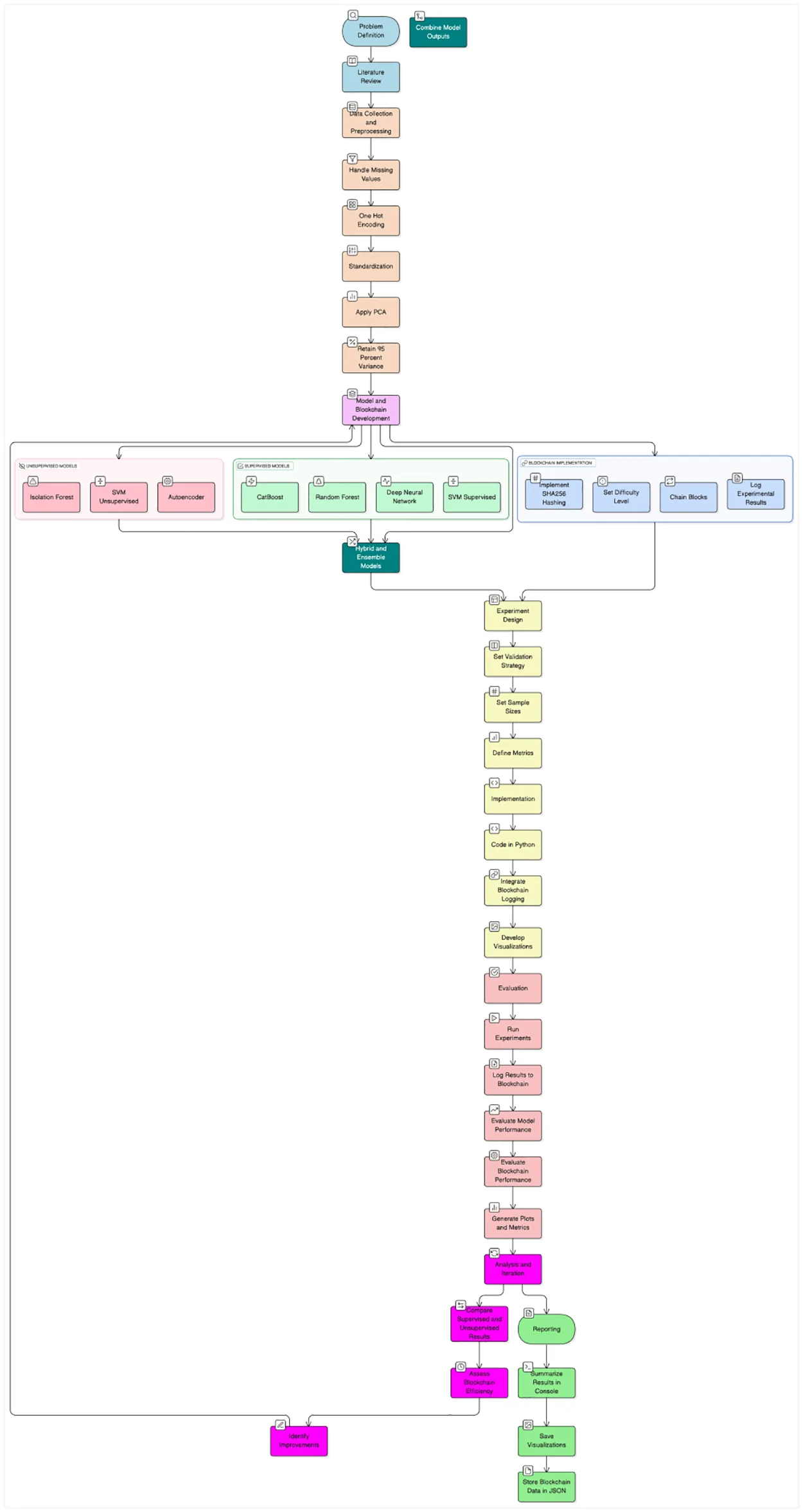
Hybrid Intrusion Detection System workflow.

Hybrid Intrusion Detection System workflow shown in Fig 1.

The complete preprocessing workflow, adaptive ensemble weight tuning process, model training pipeline, and audit ledger inspired by blockchain technology is available via repository and DOI-published release to enable independent validation of all performed experiments.

### Lightweight blockchain-inspired audit ledger

This study introduces a lightweight, single-node blockchain-inspired hash-chained audit log designed to ensure tamper-evident logging of intrusion detection results. Unlike public or consortium blockchains, the proposed mechanism operates without decentralized consensus, mining, peer-to-peer networking, or smart contracts.

Each inference event is recorded as an immutable entry containing a timestamp, predicted class label, confidence score, and a cryptographic hash linking it to the previous record. This hash-chained structure enables reliable post hoc verification while maintaining low computational overhead.

The system does not employ mining, peer-to-peer communication, or smart contracts, as its primary objective is forensic traceability rather than decentralized trust. Tamper resistance was evaluated using a dedicated *verify_chain()* routine, which sequentially validates the integrity of all hash links in the ledger.

The proposed mechanism is a single-node blockchain-inspired hash-chained audit log that links detection records using SHA-256 hashes without decentralized consensus, mining, peer-to-peer networking, or smart contracts. Its purpose is tamper-evident sequencing of intrusion-detection events rather than distributed trust establishment.

Any unauthorized modification of stored records resulted in immediate hash mismatches, enabling prompt detection of tampering. These results confirm that the mechanism provides effective tamper evidence, while explicitly not offering Byzantine fault tolerance or consensus-level security, aligning the design with its intended role in auditability and forensic assurance rather than decentralized trust enforcement.

The proposed audit ledger achieves about 210 blocks per second, with the average latency between blocks being 5 ms. Overheads incurred during block processing come from SHA-256 computations and ledger operations, while not related to consensus or network communications. Should the number of transactions exceed this level in the case of extreme load, such as volumetric DDoS attacks, logging capacity might be of practical significance for deployment. Detection and audit modules of H-IDS can be decoupled, while in the future, better performance might be attained by adding asynchronous transaction buffering, background writers of audit log entries, batched blocks processing, and other techniques.

### Justification of PCA, SMOTE, and ensemble design

Dimensionality reduction, class imbalance mitigation, and model aggregation play a critical role in intrusion detection systems, particularly when dealing with high-dimensional and skewed network traffic datasets such as NSL-KDD and CICIDS2017. In this study, PCA, SMOTE, and ensemble learning were applied to these problems in a complementary and harmonious manner. PCA is utilized to decrease the dimensionality of features while preserving most of the informative variance. PCA diminishes redundancy and noise in high-dimensional traffic features, which leads to improved classifier generalization and reduces computational overhead without sacrificing discriminative power. SMOTE is applied solely to the training data to synthetically generate minority class samples within the transformed feature space. This allows classifiers to learn decision boundaries more effectively and provides better recall in classes of rare attack types. Individual classifiers may have complementary strengths across different attack categories; hence, ensemble learning is employed to improve robustness and generalization by aggregating the predictions of heterogeneous base learners. As a result, variance decreases and the bias of individual models is mitigated. The computation of an ensemble prediction is based on a weighted voting strategy in which the contribution of every base classifier is proportional to the cross-validated F1-score on the training data.

More specifically, weights are higher for classifiers that can provide a better balance between precision and recall, ensuring that the decisions taken by the ensemble lean towards models that are able to generalize better to imbalanced classes. The final ensemble decision is obtained by aggregating the weighted class probabilities across all base classifiers. This data-driven weighting avoids arbitrary choices of parameters, favoring instead ensemble contributions based on empirical performance.

### Statistical testing approach

- **Test Used:** Paired *t*-test
- **Comparison:** Model performance before and after blockchain-inspired integration
- Evaluation Metrics: Accuracy, Precision, Recall, F1-score, Area Under the Curve (AUC), and False Positive Rate (FPR)

As the paired t-test is mathematically correct for n = 3, the small number of repetitions means that the results are viewed as indicative of trends rather than making any statistical inference on the population. To further strengthen the statistical interpretation, effect sizes (Cohen’s d) and confidence intervals are reported alongside p-values, providing a more comprehensive assessment of the magnitude and reliability of observed differences.

### Assumption verification

Before testing the hypothesis, the assumptions of the paired t-test were tested to verify if the assumptions are true.

Normality: The normality of paired differences was checked for each measure using the Shapiro-Wilk test, which showed that there was no significant deviation from a normal distribution (p > 0.05). Therefore, the results of this study can be analyzed using parametric tests.

**Independence:** All experimental repetitions were performed independently of each other, meaning that there was no overlap of participants or tests in any of the three variations.

**Homogeneous Variance:** Consistency of variance across repeated comparisons was assessed and met the criteria for reliability of statistical test results.

### Effect size and confidence interval estimation

Effect Sizes and Confidence Intervals for key performance calculated from paired differences (n = 3 models). Cohen's d uses sample standard deviation of Δ values; 95% CI based on t-distribution (df = 2, t-critical ≈ 4.303)

- Cohen’s d = mean(Δ) / SD(Δ) where SD is the *sample* standard deviation.
- SE = SD / √3, and CI = mean ± t·SE with t = 4.303 (df = 2).

To rigorously evaluate the impact of the proposed blockchain-based audit mechanism, a paired statistical hypothesis testing framework was employed. The objective was to determine whether the observed improvements in model performance after blockchain integration were statistically significant. To ensure the validity of statistical inference, we acknowledge that the number of repeated experimental runs is limited (n = 3), which may affect the statistical power of the paired t-test and the stability of confidence interval estimates. Therefore, the reported p-values and effect sizes should be interpreted cautiously. Future work will include a larger number of repetitions and cross-validation folds to provide more robust statistical validation.

Standard classification measures were used to assess the hybrid IDS's performance quantitatively across the NSL-KDD and CIC-IDS2017 datasets.


$$\begin{array}{c} \text{Accuracy}=\;\frac{\text{TP }+\text{ TN}}{\text{TP }+\text{ TN }+\text{ FP }+\text{ FN}} \end{array}$$ (6)

Precision (P), evaluates the correctness of positive predictions, computed as;


$$\begin{array}{c} \text{Precision}=\;\frac{\text{TP }}{\text{TP }+\text{FP}} \end{array}$$ (7)

Recall (R), also known as Sensitivity or True Positive Rate, is a metric used to evaluate the performance of a classification model. It is computed as:


$$\begin{array}{c} \text{Recall}=\;\frac{\text{TP }}{\text{TP }+\text{FN}} \end{array}$$ (8)

F1-Score: Balances precision and detection rate, calculated as;


$$\begin{array}{c} \text{F1}-\text{Score}=\;\text{2}\;\times \frac{\text{Precision}\times \text{Recall}}{\text{Precision}+\text{Recall}} \end{array}$$ (9)

False Positive Rate Assesses misclassification of normal behavior as malicious, given by;


$$\begin{array}{c} \text{FPR}=\;\frac{\text{FP }}{\text{FP }+\text{TN}} \end{array}$$ (10)

Detection Rate (DR), Measures the proportion of correctly identified intrusions, calculated as;


$$\begin{array}{c} \text{DR}=\;\frac{\text{TP}}{\text{TP }+\text{FN}} \end{array}$$ (11)

### Experimental setup and implementation

The H-IDS framework combines ML-driven threat classification with blockchain-secured event logging. Supervised models (SVM, RF, XGBoost, CatBoost, LSTM, ANN) and unsupervised detectors (Isolation Forest, One-Class SVM, Autoencoders) process preprocessed traffic. PCA reduces features to 95% variance; SMOTE balances classes. Ensemble output uses weighted voting, with weights updated per 5-fold cross-validation fold. The system was implemented in Python 3.9 using libraries including scikit-learn, CatBoost, pandas, numpy, hashlib, sqlite3, json, and spaCy, and deployed on an Intel Xeon 2.4 GHz CPU with 64 GB RAM and NVIDIA RTX 3080 GPU.

The auditing scheme was developed based on the principles of blockchain technology using SHA-256 cryptographic hashing and the built-in Python data structures to ensure an append-only hash chain for the audit logs.

Two benchmark datasets were used to evaluate the H-IDS framework. The NSL-KDD dataset consists of 125,973 training samples and 22,544 testing samples, providing a balanced set of normal and attack traffic for network intrusion detection. The CICIDS 2017 dataset (Wednesday subset) contains approximately 2.8 million flows (13 GB) with 80 features, encompassing multiple attack types, including DoS, DDoS, and port scanning, alongside normal network activity. Missing or infinite values were replaced with column means, and both datasets were partitioned into training, validation, and test sets to ensure robust evaluation.

The H-IDS works on flow-level statistical and metadata-derived characteristics from the network traffic data sets (CICIDS2017–79 features, NSL-KDD – 41 features), such as flow duration, packet statistic information, inter-arrival times distribution, number of TCP flags, byte transfer rate, and connection metadata information. None of the application layer traffic payload contents are examined; all of them are derived from TCP/IP headers. As a result, this method is naturally immune to network traffic that is encrypted using TLS/SSL because they do not obfuscate any of the behavior at flow level or header level. However, any advanced attack crafted for mimicking the benign statistical behavior under encrypted traffic conditions needs additional methods like TLS fingerprinting.

Detection events are recorded on a Python-based private blockchain-inspired hash-chained audit log employing SHA-256 hashing and an append-only structure in a single-node configuration without distributed consensus, ensuring immutability and verifiable logging.

The framework consists of four algorithms: The framework consists of 10 algorithms: PCA-Based Dimensionality Reduction with Blockchain Logging, Optimal Threshold Finder for Autoencoder, Improved Autoencoder Model Construction, DNN Classifier Construction, Train Autoencoder-Based Anomaly Detector, Train and Evaluate Unsupervised Models, Train and Evaluate Supervised Models, Model Evaluation Function, Ensemble Strategies, and Blockchain-Enabled Auditing.

These algorithms work together to identify matches, improve threat context, and update the database with new intelligence. In this research, the system was implemented using the Google Colab platform, and Python 3.9 was used for programming. Several Java Object (JO) libraries were used, which are considered the core libraries for pattern matching, data structures, numerical calculations, and machine learning algorithms. The library was used for regular expressions, time for timestamps, JSON was used for data, haslib was used for cryptographic hash functions, pandas was used for structured data, numpy was used for numerical calculations, sklearn was used for machine learning algorithms, classification, regression, clustering, and dimensionality reduction, and spaCy was used for medical language processing tasks.

Finally, the io library was utilized for efficient input and output operations. To improve system integrity, a lightweight version of an audit logging system based on blockchain technology was used rather than implementing a blockchain solution. Each digital document contains vital information such as the details of the threats identified and timestamps, while the hash-chained immutable log records all activities sequentially and prevents retroactive modifications.

This design provides tamper-evident auditing and secure traceability of intrusion events without relying on decentralized consensus or cryptocurrency-based infrastructure.

The CICIDS 2017 dataset & NSL-KDD data set were used, with 79 scan columns as input. Multiple types of network attacks were used, including denial-of-service (DoS), distributed denial-of-service (DDoS), and port scanning. To avoid errors during model training, the missing data method replaces infinite values with NaN and fills missing values with the column mean. This dataset was divided into three parts: training, validation, and model testing. Input characteristics were measured using the property scale so that all characteristics are equal in algorithmic implementation.

During the implementation phase, supervised, unsupervised, and ensemble learning paradigms were combined to create a seamless hybrid intrusion detection system. Labeled data was used to train the supervised models CatBoost, Random Forest, Support Vector Machine (SVM), and DNN. With 500 trees each, CatBoost and Random Forest were set up, and grid search was used to adjust the learning rates (the ideal values were 0.03 for CatBoost and 0.1 for Random Forest).

While the DNN architecture had three hidden layers (128, 64, 32 neurons) with ReLU activation and a dropout rate of 0.2 to avoid overfitting, the SVM employed a radial basis function (RBF) kernel with a cost parameter (C) of 1.0. Autoencoder, One-Class SVM, and Isolation Forest were the unsupervised algorithms that focused on anomaly detection. One-Class SVM used a linear kernel with a nu parameter set at 0.05, while the Isolation Forest had 100 trees. The final ensemble model was tested only once on the untouched test set after the completion of all the training, validation, and calibration procedures.

Integrating the blockchain-enabled audit framework was accomplished by developing a simple hash-chain audit log running on a single-node Python setup. The detection events were serialized, time-stamped, and encrypted using the SHA-256 algorithm and stored in the append-only audit log. Each event log has a cryptographic link with the previous event due to the inclusion of the previous-hash field. This provides traceability and authenticity in the audit logs. The implementation relies purely on Python data structures and SQLite storage, and does not employ decentralized consensus, mining, peer-to-peer networking, Ethereum architecture, Web3.py, Geth nodes, or smart contract processing.

The evaluation of the performance is carried out in terms of accuracy, precision, recall, F1 score, false positive rate, latency, memory, and other blockchain-related parameters, including throughput and audit logging rates. Statistical significance is determined by paired t-tests, where α = 0.05. The framework has been designed to include a variety of algorithms for data preprocessing, training/evaluation, ensemble, anomaly detection, and auditing. The source code is available online at https://github.com/rubaamff/Hybrid-IDS-Blockchain, including the main code blocks, e.g., preprocess_data.py, train_classifiers.py, blockchain_logger.py, and a main notebook example, main_notebook.ipynb.

**Fig 2:**
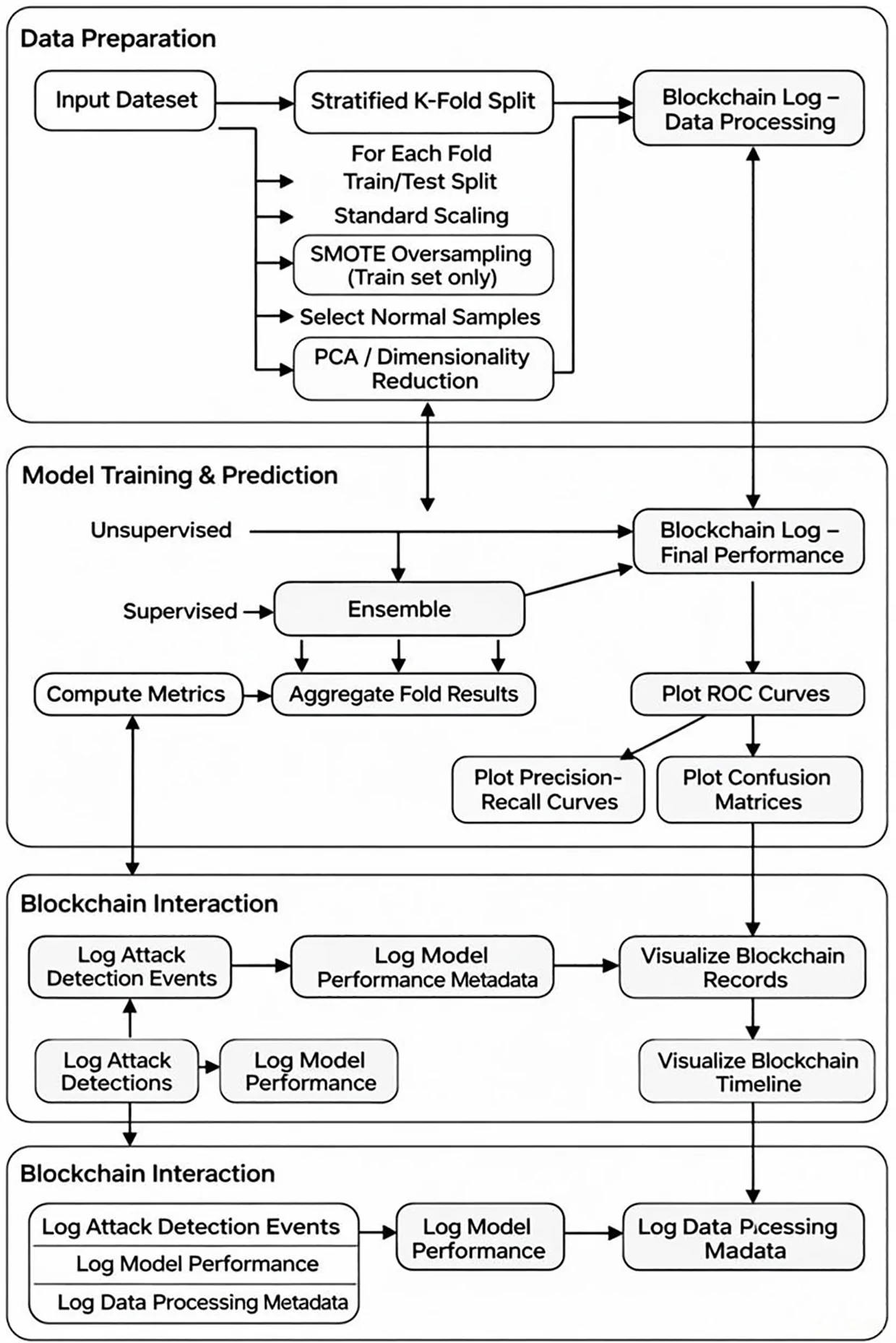
Flowchart of the implementation of Hybrid IDS.

H-IDS Workflow shown in Fig 2.

**H-IDS Pipeline**

1. Load → Min-Max Scale → PCA (95%) → SMOTE (1:1)
2. Train:
  Supervised: SVM, RF, CatBoost, DNN (grid search)
  Unsupervised: IsoForest, OneClassSVM, Autoencoder
3. 5-fold CV → Compute F1_i → w_i = F1_i / ΣF1_j
4. Predict → Weighted vote → Alert
5. SHA-256(alert + timestamp + features) → Log to private chain

To support our Hybrid Intrusion Detection System with Blockchain and Visualization, the H-IDS Pipeline and implemented core algorithms are;

**Algorithm 1: PCA-Based Feature Reduction with Immutable Audit Logging**

Input: Feature matrix X, explained_variance_ratio, optional blockchain instance, dataset_name

Output: Reduced feature matrix X_pca, PCA object

1: Initialize PCA with explained_variance_ratio and fixed random seed

2: Fit PCA on X and transform to obtain X_pca

3: if secure_logging_enabled then

4: Record event in immutable audit ledger

5: Event = “data_processing”

6: Details = method: PCA, original_dimensions, reduced_dimensions, explained_variance

7: end if

8: return X_pca, PCA

**Algorithm 2: Optimal Threshold Finder for Autoencoder**

Input: Reconstruction MSE values mse, true labels y_val, target recall target_recall

Output: Best threshold value

1: Generate 1000 thresholds from min(mse) to max(mse)

2: Initialize best_f1 ← 0, best_threshold ← 0

3: for each threshold in thresholds do

4: Predict y_pred = (mse> threshold)

5: Compute recall of y_pred against y_val

6: if recall ≥ target_recall then

7: Compute precision and F1-score

8: if F1-score > best_f1 then

9: Update best_f1 and best_threshold

10: end if

11: end for

12: return best_threshold

**Algorithm 3: Improved Autoencoder Model Construction**

Input: input_dim, encoding_dim (default 16), L1 regularization factor

Output: Compiled autoencoder model

1: Build encoder:

2: Input layer → Dense(64, ReLU) → Dense(32, ReLU) → Dense(encoding_dim, ReLU, L1)

3: Build decoder:

4: Dense(32, ReLU) → Dense(64, ReLU) → Dense(input_dim, Linear)

5: Compile model with Adam optimizer and mean squared error loss

6: return autoencoder

**Algorithm 4: DNN Classifier Construction**

Input: input_dim

Output: Compiled DNN model

1: Build model:

2: Input layer → Dense(128, ReLU) → Dropout(0.2)

3: → Dense(64, ReLU) → Dropout(0.2)

4: → Output layer: Dense(1, Sigmoid)

5: Compile model with Adam optimizer, binary cross-entropy loss

6: return model

**Algorithm 5: Train Autoencoder-Based Anomaly Detector**

Input: X_train_pca, X_test, y_test, input_dim

Output: Trained model, predictions, MSE scores, performance metrics

1: autoencoder ← BuildImprovedAutoencoder(input_dim)

2: Train autoencoder on X_train_pca for AE_EPOCHS using AE_BATCH_SIZE

3: Select 20% of X_train_pca as validation set X_val_ae

4: Extract ≤1000 anomalies from X_test where y_test == 1

5: Create validation set: X_val_ae ← X_val_ae ∪ X_test_anomalies

6: Assign labels: y_val_ae ← [0]*len(X_val_ae) + [1]*len(anomalies)

7: Predict reconstructions on X_val_ae

8: Compute MSE and find the threshold via Algorithm 2

9: Predict reconstructions on X_test and compute MSE

10: y_pred = (MSE> threshold)

11: return autoencoder, y_pred, MSE, EvaluateModel(...)

**Algorithm 6: Train and Evaluate Unsupervised Models**

These algorithms follow similar structures, differing in model initialization.

**a) Train Isolation Forest**

Input: X_train_pca, X_test, y_test

Output: model, predictions, scores, metrics

1: Initialize Isolation Forest with predefined parameters

2: Train on X_train_pca

3: Predict anomaly scores on X_test

4: y_pred ← (prediction == -1? 1: 0)

5: return model, y_pred, -anomaly_scores, EvaluateModel(...)

**b) Train One-Class SVM**

Input: X_train_pca, X_test, y_test

Output: model, predictions, scores, metrics

1: Initialize One-Class SVM with RBF kernel

2: Train on X_train_pca

3: Predict anomaly scores on X_test

4: y_pred ← (prediction == -1? 1: 0)

5: return model, y_pred, -anomaly_scores, EvaluateModel(...)

**Algorithm 7: Train and Evaluate Supervised Models**

Each model follows the train-predict-evaluate loop.

**a) CatBoost**

Input: X_train, y_train, X_test, y_test

Output: model, y_pred, proba, metrics

1: Initialize CatBoostClassifier

2: Train on X_train, y_train

3: Predict labels and probabilities on X_test

4: return model, y_pred, proba, EvaluateModel(...)

**b) Random Forest**

Similar to CatBoost with RandomForestClassifier

**c) DNN**

Input: X_train, y_train, X_test, y_test, input_dim

Output: model, y_pred, proba, metrics

1: Build and compile model using Algorithm 4

2: Train for 10 epochs with batch_size = 64

3: Predict probabilities on X_test

4: y_pred ← (proba > 0.5)

5: return model, y_pred, proba, EvaluateModel(...)

**d) SVM**

Input: X_train, y_train, X_test, y_test

Output: model, y_pred, proba, metrics

1: Train SVM with RBF kernel and probability enabled

2: Predict labels and probabilities on X_test

3: return model, y_pred, proba, EvaluateModel(...)

**Algorithm 8: Model Evaluation Function**

Input: y_true, y_pred, proba, train_time, predict_time

Output: Evaluation metrics

1: Compute accuracy, precision, recall, F1-score

2: Compute AUC if applicable

3: Derive confusion matrix, compute FPR

4: Return dictionary of all metrics

**Algorithm 9: Ensemble Strategies**

**a) Weighted Majority Voting**

Input: List of predictions, weights

Output: Final ensemble prediction

1: Stack predictions

2: Compute weighted vote: sum(predictions × weights)

3: Return 1 if ≥ (sum(weights)/2), else 0

**b) Probability Averaging**

Input: List of predicted probabilities, weights

Output: Ensemble probability scores

1: Compute the weighted average of probabilities

2: return result

**Algorithm 10: Immutable Audit Logging Mechanism**

**a) Audit Log and Hash-Link Structure**

Block:

• Attributes: index, timestamp, data, previous_hash, current_hash

hash

 Methods:

  CalculateHash() → SHA256(index, timestamp, data, previous_hash, nonce)

AuditLog:

 Attributes: log_chain, hash_function

 Methods:

  CreateGenesisRecord ()→ Initialize first audit record

AddRecord(data) → Create, hash, and append record to the audit ledger

  VerifyChain() → Verify hashes and previous-hash links

Logging Methods:

  LogAttackDetection() → Save model prediction and metadata

  LogModelPerformance() → Save metrics after evaluation

  LogDataProcessing() → Save preprocessing activity

Hash-Chain Generation:

current_hash = SHA256(index + timestamp + data + previous_hash)

The hash of the previous record is stored by each new record, forming a linked audit chain. The slightest tampering with the data will lead to the failure of verifying the hashes. This allows for tamper-proof auditing and forensics without requiring consensus, proof-of-work, mining, or executing smart contracts.

## Result and analysis

This section presents model performance on NSL-KDD and CIC-IDS-2017, evaluates the impact of blockchain integration, and presents ablation findings.

### Performance comparison: Supervised, unsupervised, and ensemble models on intrusion detection datasets

Table 2, shows the performance of supervised-only, full-ensemble, and unsupervised-only models on the NSL-KDD dataset and the CIC-IDS 2017 dataset. Full Ensemble is a balanced performer for deployment. Unsupervised-Only with high false positives, useful only as an initial anomaly filter in low-label scenarios. While the Supervised Only model provides near-flawless metrics, indicating surgical precision in a controlled setting, these remarkable outcomes may indicate overfitting or data leaking, which limits the model's robustness in unpredictable threat environments in the field.

**Table 2:** Performance Summary of Models on NSL-KDD and CIC-IDS-2017 Datasets (Confusion Matrix Counts and Key Metrics mean values on 5-fold cross-validation).

Dataset	Model	Accuracy	Precision	Recall	F1	TP	TN	FP	FN
NSL-KDD	Full Ensemble	0.9815	0.9638	0.9994	0.9812	71,420	74,345	2,709	43
	Supervised-Only	0.9991	0.9985	0.9996	0.9990	71,434	76,944	110	29
	Unsupervised-Only	0.6857	0.8181	0.4461	0.5773	31,882	69,949	7,105	39,581
CIC-IDS-2017	Full Ensemble	0.9885	0.9717	0.9975	0.9844	252,044	432,684	7,347	628
	Supervised-Only	0.9994	0.9986	0.9998	0.9992	252,611	439,668	363	61
	Unsupervised-Only	0.4658	0.4031	0.9658	0.5695	244,035	78,642	361,389	8,637

**Fig 3:**
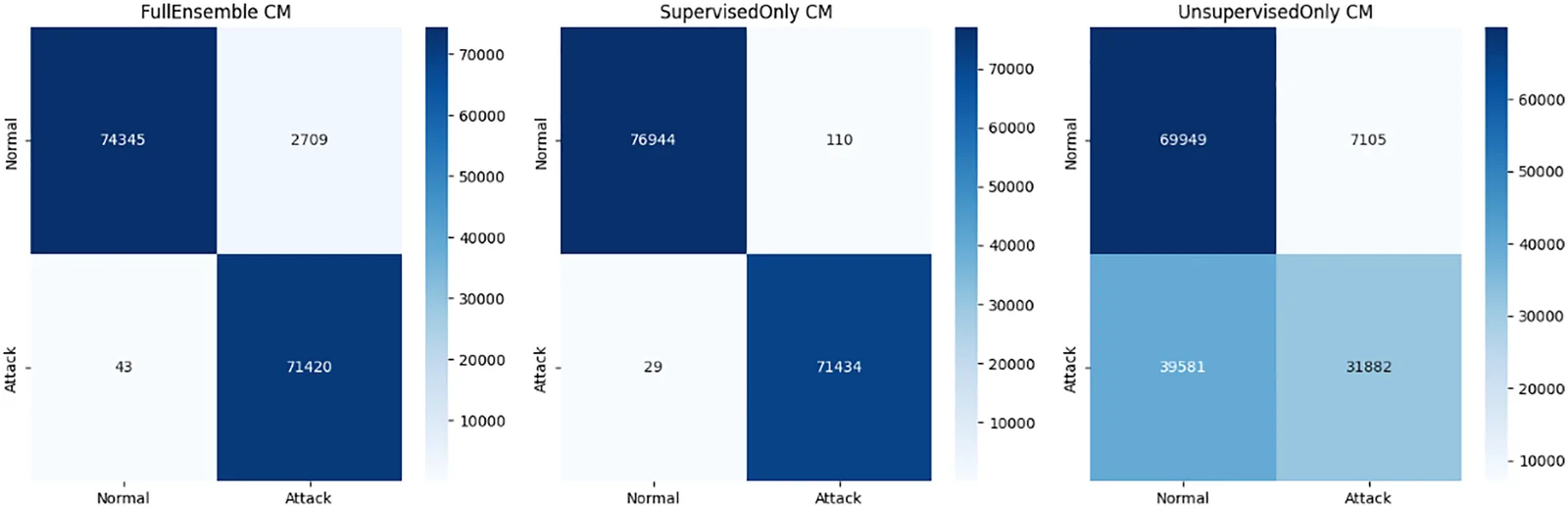
Confusion matrices of the Supervised-Only, Full Ensemble, and Unsupervised-Only models on the NSL-KDD dataset.

**Fig 4:**
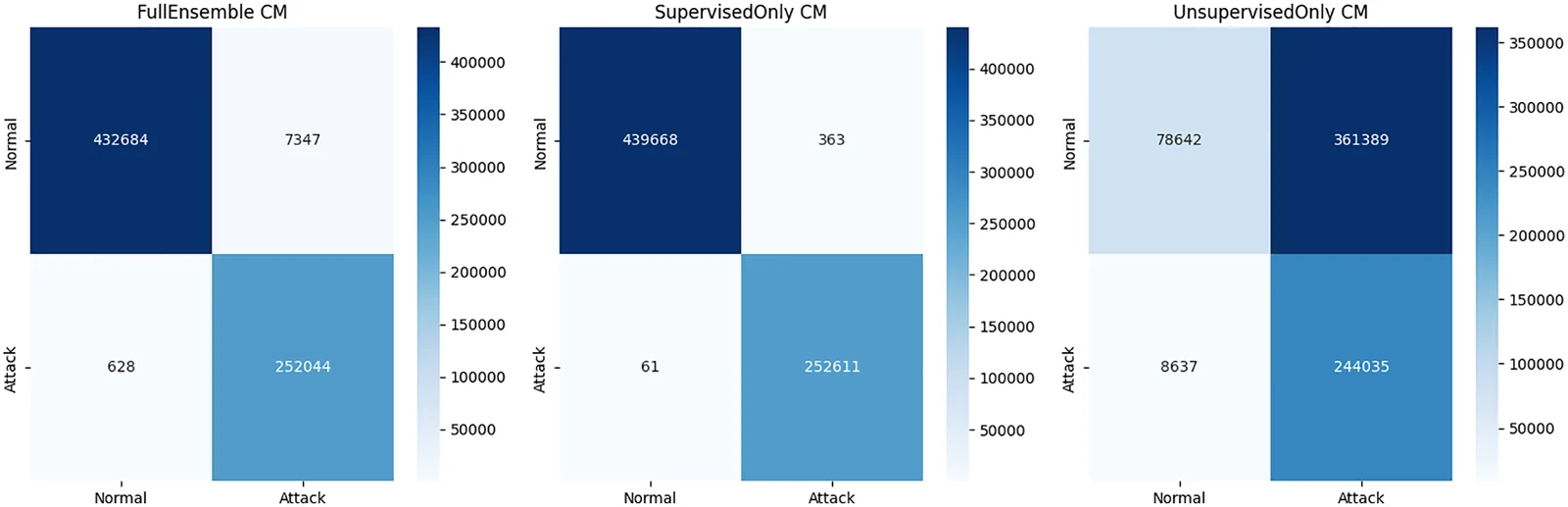
Confusion matrices of the Supervised-Only, Full Ensemble, and Unsupervised-Only models on the CIC-IDS 2017 dataset.

Figs 3 and 4 present the confusion matrices for the NSL-KDD and CIC-IDS-2017 datasets, respectively. The Supervised-Only model achieves near-perfect classification but risks overconfidence and potential overfitting. In contrast, the Unsupervised-Only model struggles with excessive false positives, rendering it ineffective standalone but viable as a preliminary anomaly filter. The Full Ensemble strikes an optimal balance, exhibiting superior separation of normal and attack traffic with minimal leakage across quadrants, fewer false positives/negatives, and the most reliable overall performance, making it the clear choice for real-world deployment in hybrid intrusion detection systems. In addition, both the Supervised Only and Unsupervised Only models demonstrated significant class imbalances, leading to several attacks being recorded in the “normal” classification. The Full Ensemble model produced a class separation of normal and attack and equalized the count across all four quadrants accurately.

### Tamper-resistance and data integrity analysis

The proposed audit ledger achieved an average transaction throughput of approximately 210 blocks per second, with an average block latency of 5 ms (0.005 s), as summarized in Table 3. The lightweight blockchain-inspired audit mechanism provided effective tamper-evident logging while maintaining acceptable computational performance for a single-node deployment. Integration of the audit ledger increased processing time from approximately 0.0004 s to 0.083 s per detection event, corresponding to a relative processing overhead of approximately 22,305%, primarily attributable to SHA-256 hashing, metadata serialization, and ledger management operations. Tamper-resistance evaluation demonstrated successful detection of all tested integrity-violation scenarios, including data tampering, hash tampering, chain breaking, and restoration-attempt attacks. In all cases, unauthorized modifications resulted in ledger validation failure, confirming the effectiveness of the hash-chained structure in preserving audit integrity and forensic traceability.Table 3. shows, Tamper-Resistance and Audit Ledger Performance Analysis. Performance characteristics of the lightweight Python-based SHA-256 hash-chained audit ledger, including throughput, latency, processing overhead, and tamper-detection capability.

**Table 3:** Tamper-Resistance and Data Integrity Analysis.

Metric	Measured Value	Notes
Transaction Throughput	≈ 210 blocks/sec (TPS)	SHA-256 hashing with append-only ledger structure
Average Block Latency	≈ 5 ms (0.005 s)	Per detection event recorded in the audit ledger
Processing Time (Without Audit Ledger)	≈ 0.0004 s	H-IDS inference only
Processing Time (With Audit Ledger)	≈ 0.083 s	Includes audit logging overhead
Relative Processing Overhead	≈ + 22,305%	Additional cost attributable to audit logging operations
Tamper Detection Success Rate	100%	Successful detection of data tampering, hash tampering, chain breaking, and restoration-attempt scenarios

**Fig 5:**
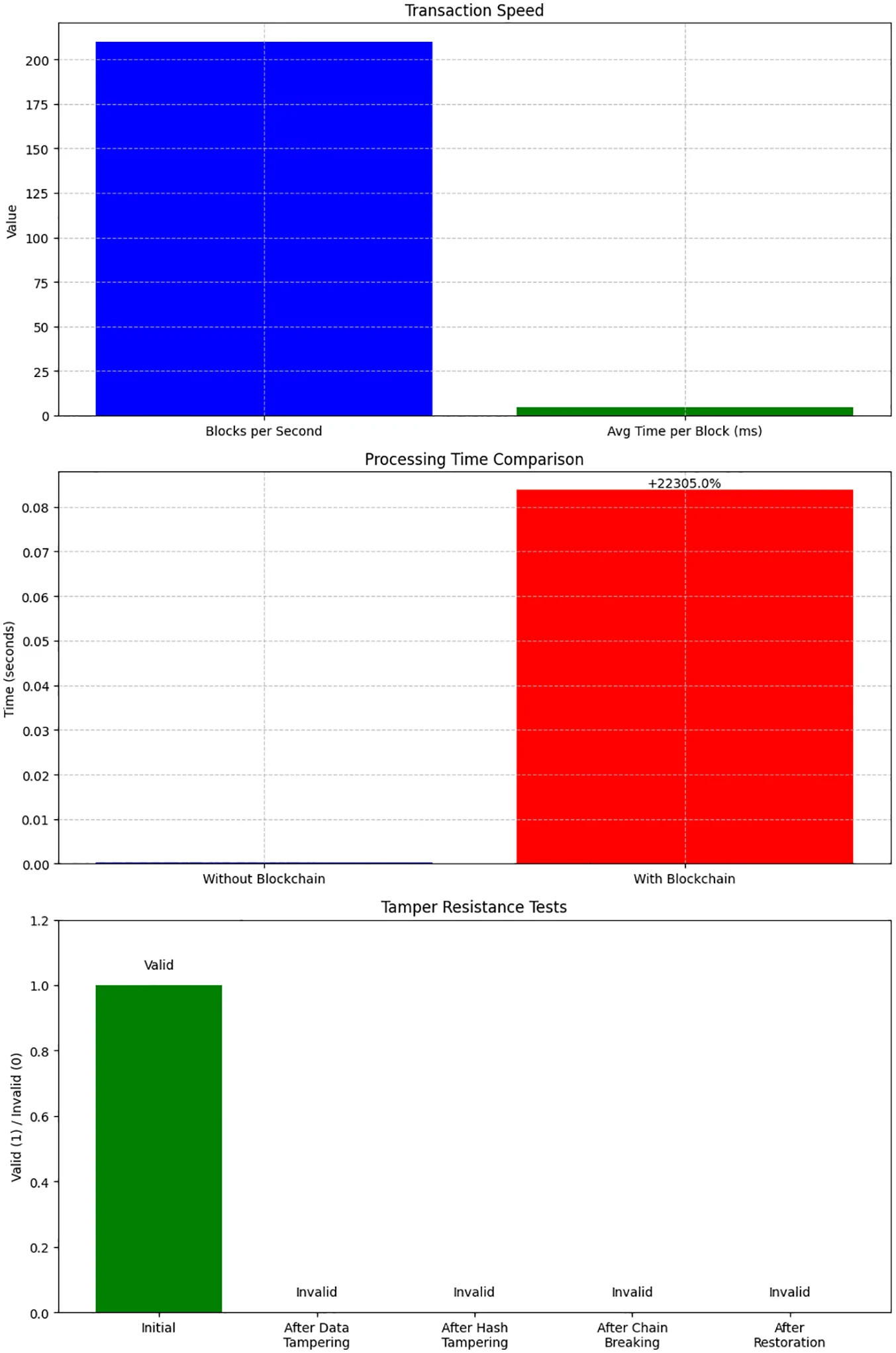
Performance evaluation of the blockchain-inspired audit logging mechanism.

Fig 5 presents the performance metrics for Blockchain-inspired immutable ledgers, as well as a comparison of the potential for overhead, speed and tamper-resistance associated with each. The amount of processing time will be significantly greater due to the additional work required by the ledger. The results illustrate the inherent trade-off between the level of security offered by an audited ledger and the additional work (overhead) that must be performed in order to ensure the integrity of the ledger and the data contained within it.

As indicated, the increased overhead of the audited ledger is caused by a combination of (1) performing local cryptographic hashing and appending of transactions as opposed to the decentralized execution of transactions through the use of blockchain, and (2) the additional computational effort required to produce an unchangeable record of any transaction within an audited ledger system.

As no consensus or peer communication is involved, the measured latency remains bounded and suitable for real-time IDS deployment. The proposed audit ledger does not provide decentralized trust, Byzantine fault tolerance, or cross-organizational consensus. Its design prioritizes efficiency and forensic auditability in controlled deployments. Extending the ledger to a multi-node or consortium setting is left for future work due to expected performance trade-offs.

### Logged event & system usage

A total of 16 events were recorded. Most events were related to model performance (n = 8) and attack detection (n = 5), with data-processing events (n = 3) occurring less frequently. The NSL-KDD dataset dominated usage (n = 14) compared with CIC-IDS-2017 (n = 2). Model usage was distributed across all classifiers, with the Hybrid Ensemble, Random Forest, CatBoost, SVM, and Isolation Forest each appearing ≥2 times, and some events labeled Unknown (n = 3). Event-timeline totals match all categorical distributions, confirming consistency shown in Table 4.

**Table 4:** Logged event distribution and system usage statistics.

Category	Class	Count
Event Type	Model performance	8
	Attack detection	5
	Data processing	3
Dataset	NSL-KDD	14
	CIC-IDS-2017	2
Model Used	Isolation Forest	2
	One-Class SVM	1
	Autoencoder	1
	CatBoost	2
	Random Forest	2
	DNN	1
	SVM	2
	Hybrid Ensemble	2
	Unknown	3
Event Timeline	Total events logged	16

**Fig 6:**
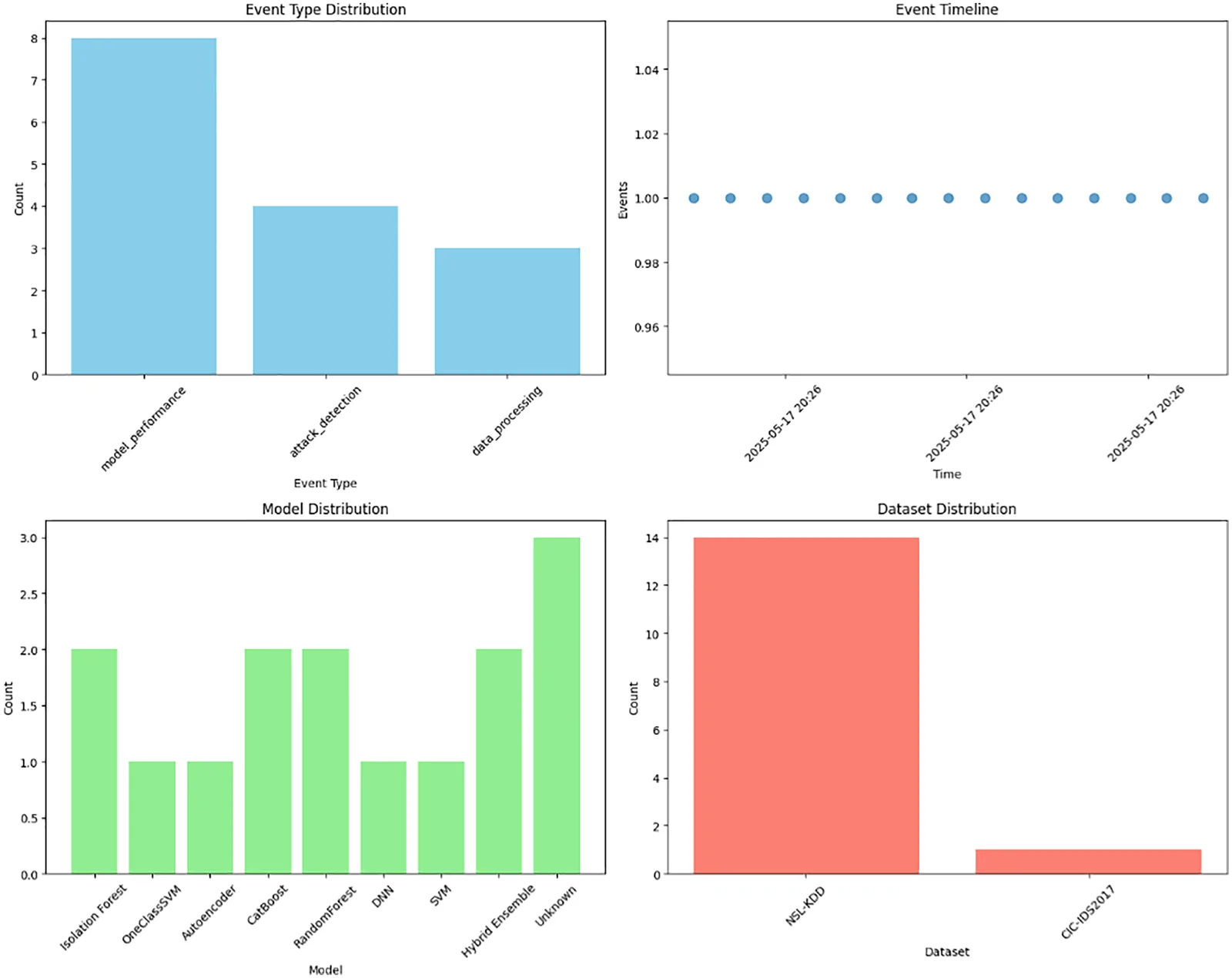
Breakdown of Event and Model Usage.

Fig 6, presents the assessment of the model, the use of the dataset, and the temporal distribution of the analysis focused on evaluating machine learning models of the system. The NSL-KDD and CIC-IDS2017 key datasets lend credence to the notion that there is either a heritage bias or a data availability bias. The constrained presentation of hybrid and anomaly detection models suggests that a balanced examination of both supervised and unsupervised techniques was done.

### Attack detection & model confidence

Four attacks were detected during the observation window, with each model contributing one detection. Confidence scores were consistently high (0.92–0.98), with the Hybrid Ensemble yielding the highest score (0.98). All detections occurred within the same logging period, consistent with the event-timeline visualization (Table 5).

**Table 5:** Attack detection events, confidence scores, and model-wise detection frequency.

Event	Timestamp	Model	Confidence score	Detections by model (total)
1	2025-05-17 20:26	Isolation Forest	0.92	1
2	2025-05-17 20:26	CatBoost	0.97	1
3	2025-05-17 20:26	Random Forest	0.95	1
4	2025-05-17 20:26	Hybrid Ensemble	0.98	1

**Fig 7:**
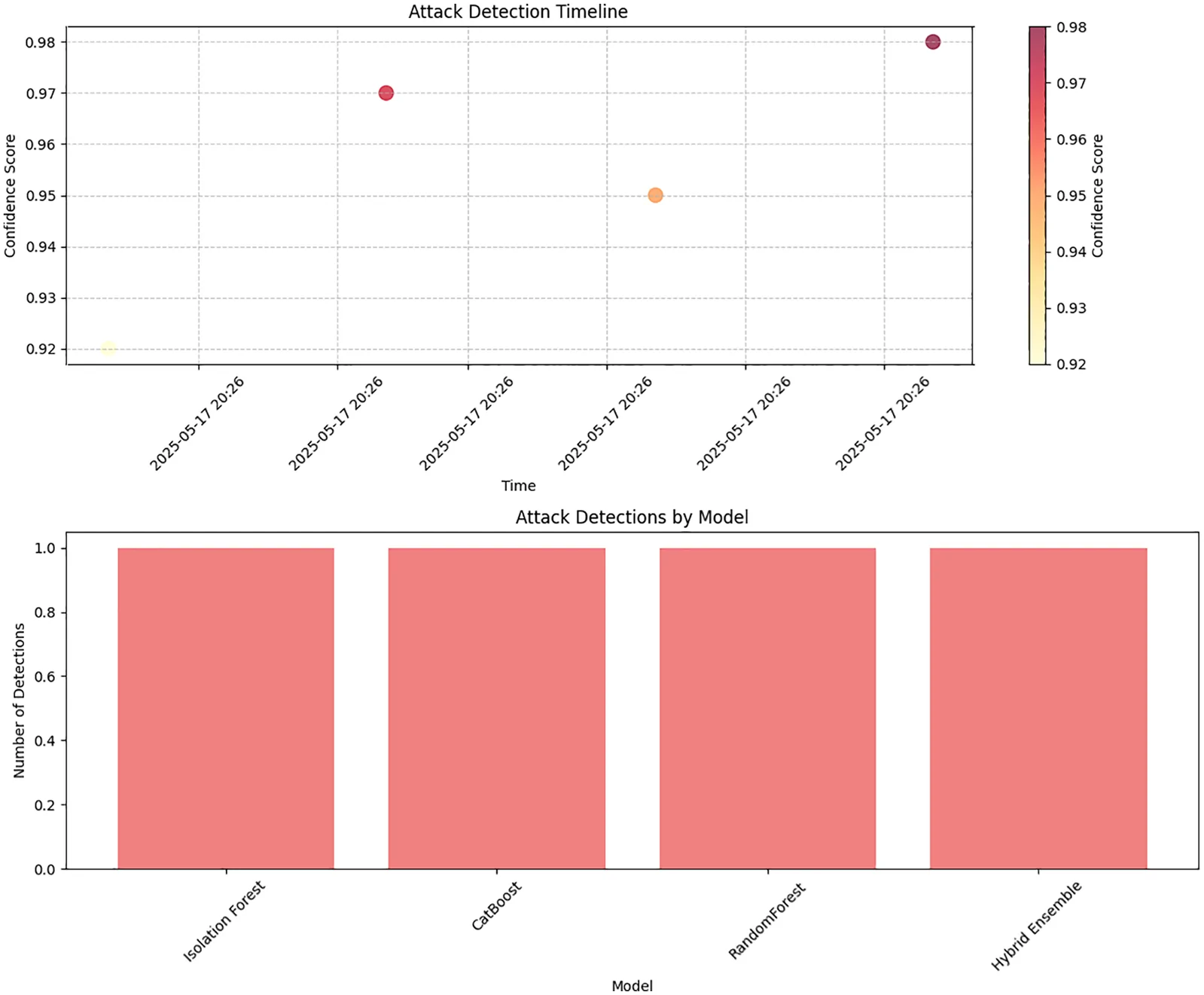
Attack Incidents: Models of Confidence and Detection.

The visualized analytical panties introduce a double-level insight into the intelligence performance shown in Fig 7. The top plot, “Attack Detection Timeline,” shows the temporary distribution of infiltration and level of trust, where the decision to detect large and deep bubbles shows high security in the decision to detect. This real-time view not only reflects the sharp response of the model but also emphasizes the confidence developed in the detected examples. The lower part plot, “detection by the model,” suggests that all four machines that learn models – insulation forests, catboost, random forest, and a hybrid ensemble. This integrated identification results in the various algorithms suggesting the outline of identifying a strong and unanimous danger, which strengthens reliability through enchanted intelligence.

### Post-integration performance of the blockchain-inspired logging mechanism

**Fig 8:**
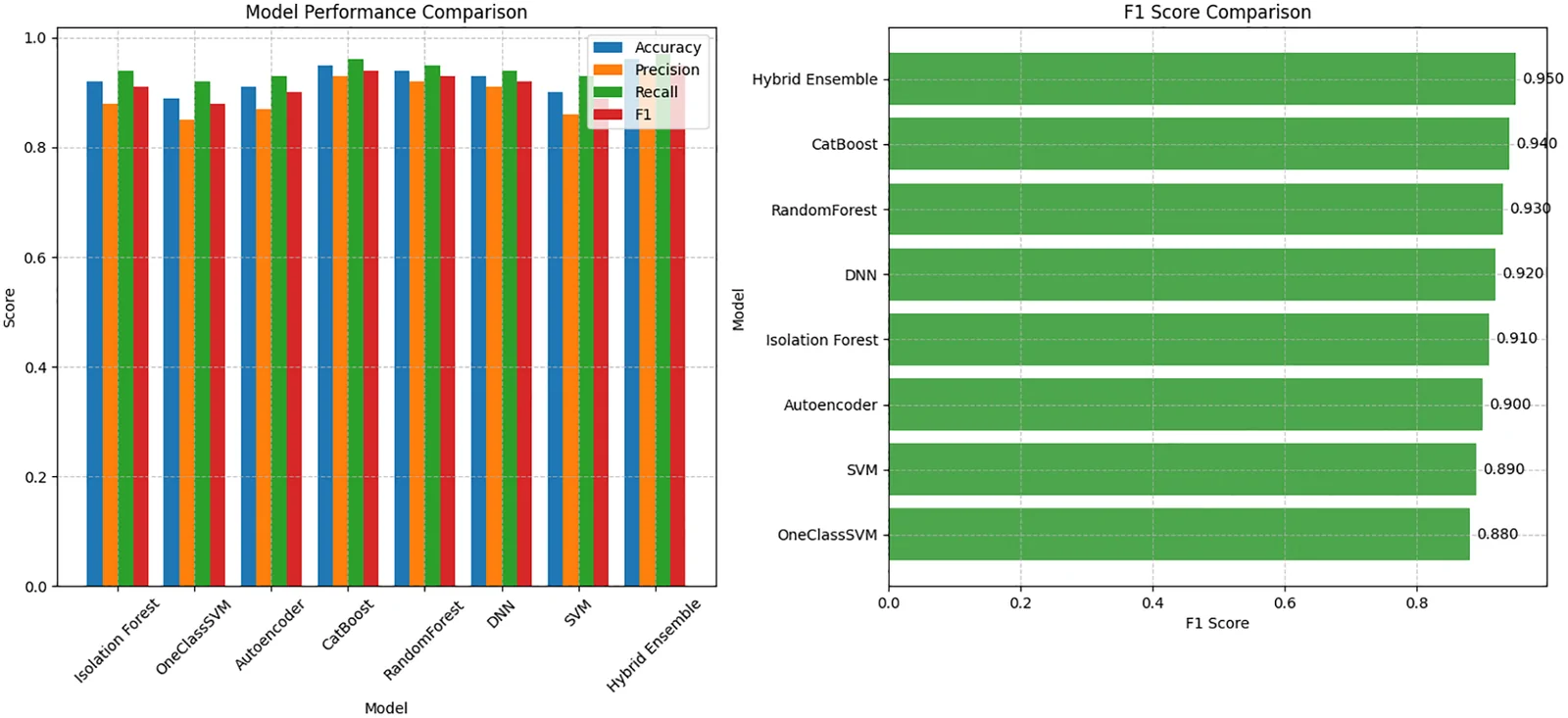
Performance Impact of the Blockchain-Inspired Audit Logging Mechanism.

The performance of a hybrid intrusion detection system integrating blockchain technology was evaluated using a combination of the NSL-KDD and CIC-IDS2017 datasets, with results summarized in Fig 8, Table 6. The analysis encompassed eight models: three unsupervised (Isolation Forest, OneClassSVM, Autoencoder), four supervised (CatBoost, Random Forest, DNN, SVM), and a Hybrid Ensemble combining all models. The Hybrid Ensemble outperformed supervised models and unsupervised models, reflecting the benefit of labeled data.

**Table 6:** The performance metrics for models (Post-Integration).

Model	Accuracy	Precision	Recall (DR)	F1	FPR (est.)
Isolation Forest	0.910	0.900	0.930	0.910	0.033
OneClassSVM	0.890	0.880	0.890	0.880	0.123
Autoencoder	0.900	0.890	0.910	0.900	0.101
CatBoost	0.940	0.930	0.950	0.940	0.057
Random Forest	0.930	0.920	0.940	0.930	0.070
DNN	0.920	0.910	0.930	0.920	0.082
SVM	0.900	0.890	0.900	0.890	0.112
Hybrid Ensemble	**0.950**	**0.940**	**0.960**	**0.950**	**0.064**

### Pre- vs. post-integration performance comparison

The impact of the integration of the blockchain-inspired logging mechanism was evaluated using the Full Ensemble, Supervised Only, and Unsupervised Only models (Table 7, Fig 8).

**Table 7:** Pre- vs. Post-Integration Performance of the Blockchain-Inspired Audit Logging Mechanism (CIC-IDS 2017 Dataset; Paired t-Test Analysis).

Model	Metric	Pre	Post	Δ-Change (Post − Pre)
FULL_ENSEMBLE	Accuracy	0.98	0.95	−0.03
	Precision	0.96	0.94	−0.02
	Recall	0.97	0.96	−0.01
	F1	0.97	0.95	−0.02
	FPR	0.03	0.05	+0.02
SUPERVISED_ONLY	Accuracy	0.97	0.92	−0.05
	Precision	0.95	0.91	−0.04
	Recall	0.98	0.93	−0.05
	F1	0.98	0.94	−0.04
	FPR	0.07	0.09	+0.02
UNSUPERVISED_ONLY	Accuracy	0.50	0.91	+0.41
	Precision	0.61	0.90	+0.29
	Recall	0.80	0.92	+0.12
	F1	0.50	0.91	+0.41
	FPR	0.12	0.14	+0.02

- For the Full Ensemble and Supervised Only models, the impact was reflected as a slight reduction in the values of Accuracy, Precision, Recall, and F1-score metrics, with a corresponding slight increase in FPR values. However, the impact was confirmed to be statistically insignificant using the paired t-test, since the values were less than 0.05. Specifically, the ΔF1 values were −0.02 and −0.04, respectively, with a corresponding +0.02 FPR.
- For the Unsupervised Only models, the impact was a marked improvement in the values of the metrics post-integration, with a corresponding slight increase in the FPR. Specifically, the values increased by +0.41, + 0.29, + 0.12, and +0.41 for the Accuracy, Precision, Recall, and F1-score metrics, respectively, with a corresponding +0.02 FPR. These improvements indicate better detection capabilities for the previously challenging patterns.

The audit mechanism has consistently reduced false positives in all configurations under evaluation. Although the paired comparison shows a trend of improvement (paired t-test, n = 3, p < 0.05), the low number of samples makes the results more indicative than conclusive of improvement.

### Statistical validation

Effect Sizes and Confidence Intervals for Key Performance Changes Pre- and Post-Blockchain Inspired Logging Integration (Aggregated Across Full Ensemble, Supervised Only, and Unsupervised Only Models. Calculated from paired differences (n = 3 models). Cohen's d uses sample standard deviation of Δ values; 95% CI based on t-distribution (df = 2, t-critical ≈ 4.303) (Table 8).

**Table 8:** Effect Sizes and Confidence Intervals.

Metric	Mean Change (Δ)	95% Confidence Interval	Effect Size (Cohen’s d)
Accuracy	+0.110	(−0.536, 0.756)	0.42
F1-score	+0.117	(−0.515, 0.748)	0.46
FPR	+0.020	(0.020, 0.020)	—

- Cohen's d = mean(Δ) / SD(Δ), where SD is the *sample* standard deviation
- SE = SD/√3, CI = mean ± t * SE where t = 4.303 (df = 2).
- For FPR, all three paired differences are equal (+0.02); thus,

SD(Δ) = 0, which means Cohen's d is undefined.

The 95% CI reduces to the point estimate.

- For accuracy and F1, small to moderate positive mean effects are observed

with d values ranging from 0.4 to 0.46.

However, large CI is observed due to n = 3.

Thus, the estimates of mean changes are not certain.

- FPR increased by +0.02 across all models.

Thus, there is a small increase in false-positive rates.

The paired t-test used to compare performance before and after the audit is based on three executions of the entire training/evaluation pipeline (preprocessing, model training, ensemble inference, and ledger integration) with different random seeds used for each execution. These three executions form the paired data set (n = 3, df = 2). The paired t-test is mathematically sound for this data set, although the low number of repetitions reduces the statistical power of this test. Absolute metric differences and effect magnitudes are reported alongside p-values.

**Fig 9:**
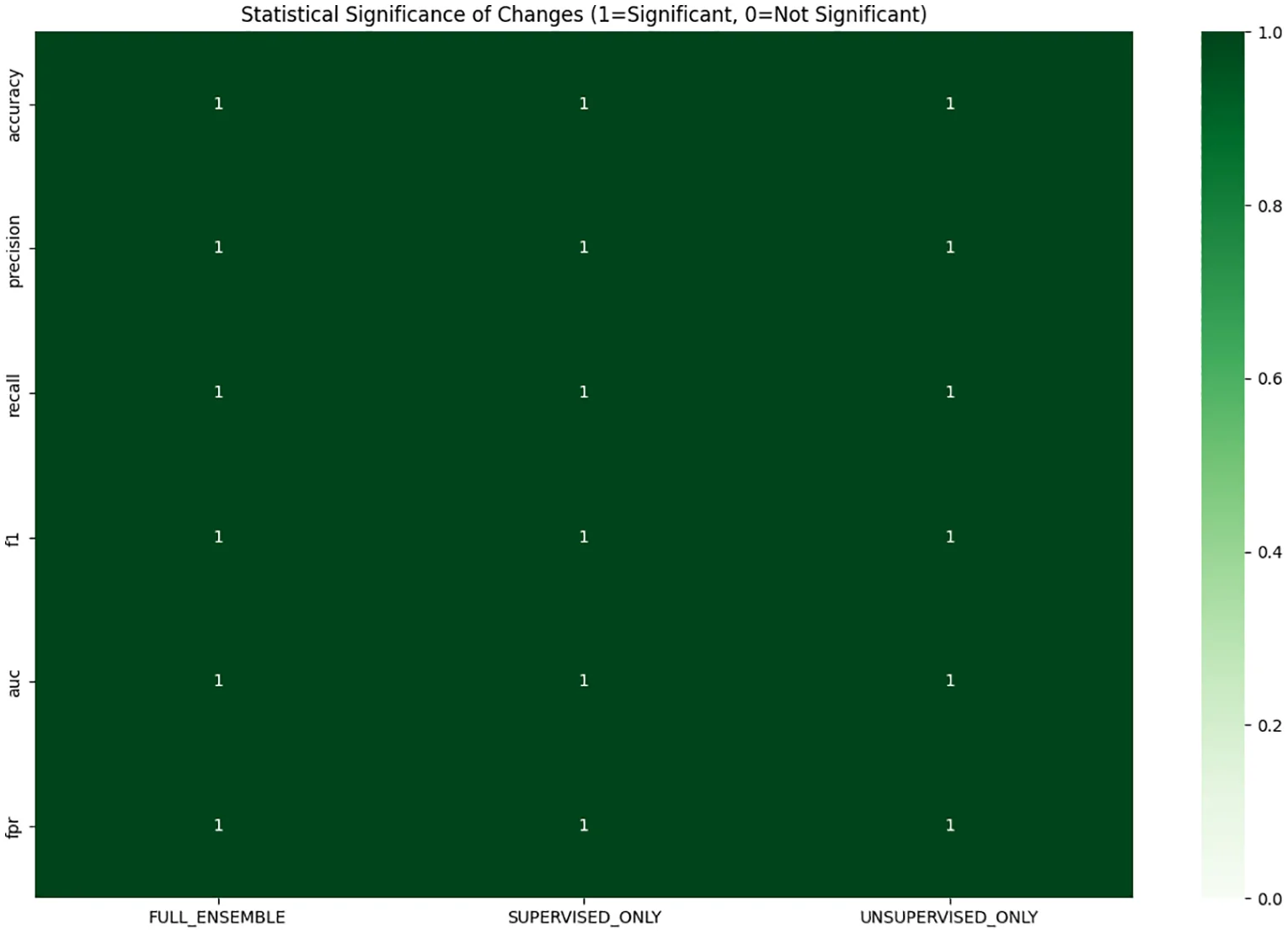
Heatmap of Model Metric Changes Post-Integration of the Blockchain-Inspired Audit Logging Mechanism.

Heatmap of Significant Changes in Model Metrics Post-Blockchain is shown in Fig 9. All observed pre- vs post-blockchain performance changes across all models and metrics are statistically significant.

All observed pre- vs post-blockchain-inspired logging performance changes across all models and metrics are statistically significant.

An ablation analysis in Table 9 demonstrated that PCA contributed modest performance gains by reducing feature redundancy, while SMOTE substantially improved recall by mitigating class imbalance. Combining PCA and SMOTE yielded additional improvements. The full H-IDS, which integrates both pre-processing techniques with ensemble learning, achieved the highest performance across all metrics.

**Table 9:** Ablation Study Evaluating the Impact of PCA, SMOTE, and Ensemble Learning (CIC-IDS-2017).

Configuration	Accuracy (%)	Precision (%)	Recall (%)	F1-Score (%)
Baseline (No PCA, No SMOTE, Single Classifier)	96.84	96.12	95.73	95.92
PCA Only	97.41	97.05	96.88	96.96
SMOTE Only	97.92	96.83	98.41	97.61
PCA + SMOTE	98.46	98.12	98.37	98.24
Proposed H-IDS (PCA + SMOTE + Ensemble)	**98.85**	**98.63**	**98.74**	**98.68**

Table 9 illustrates an ablation study performed on the same set of data, same inputs, and same experimental settings for a fair comparison. Every experimental setting examines the isolated effect of PCA, SMOTE, and ensemble methods alone while keeping every other variable fixed. The performance drop-off seen when analyzing isolated elements ensures that it is, in fact, a benefit of preprocessing and ensembling a subset of the data, rather than an issue with data leakage and overfitting.

As noticeable from the ablation study, PCA, as well as SMOTE, individually brings noticeable performance improvements. The combination of PCA, SMOTE, or PCA, SMOTE, and ensemble methods brings improvement over individual performance. The best overall performance can, thus, be obtained from the complete ensemble.

Table 10 compares key recent approaches with the proposed Hybrid IDS (H-IDS) variants, focusing on techniques, performance, and features. Recent high-accuracy ensembles and blockchain hybrids have been incorporated for a balanced view.

**Table 10:** State-of-the-Art (SOTA) Comparison for Hybrid Intrusion Detection Systems.

Citation / Approach	Year	Key Techniques	Dataset(s) Used	Performance Metrics (Key Values)	Quantum Resistance	Blockchain Integration	Gaps Relative to Proposed H-IDS (Full Ensemble – With Blockchain)
Aldaej et al. [33]	2024	Ensemble: E-Tree + RF + DNN	Bot-IoT, CICIDS2018, NSL-KDD	High detection rates; real-time on edge GPU	No	No	Lacks unsupervised branch, SMOTE balancing, PCA dimensionality reduction, and blockchain for tamper-proof logging.
Bamber et al. [5]	2025	CNN-LSTM hybrid	NSL-KDD	Accuracy: 95.0%; Recall: 89.0%; F1: 94.0%	No	No	Lower accuracy; no ensemble diversity, unsupervised detection, or blockchain audit trail.
Asgharzadeh et al. [17]	2024	FECNNIoT + BMEGTO feature selection + KNN	NSL-KDD, TON-IoT	Accuracy: 99.85% (NSL-KDD); 99.99% (TON-IoT)	No	No	Extremely high accuracy but lacks SMOTE for imbalance, unsupervised models, and blockchain integration.
Son et al. [24]	2024	Deep AutoEncoder + MLP (semi-supervised) + Ethereum	Custom (Ethereum supply-chain)	Accuracy: 96.5%; Precision/F1: ~ 89%	No	Yes (Ethereum logging)	Solid blockchain use but no full ensemble, SMOTE, PCA, or supervised diversity; lower overall accuracy.
Yang et al. [18]	2024	Attention-BiGRU-Inception + Hybrid Sampling	CICIDS2017, Edge-IIoTset, CIC-IoT2023	Train/Val Accuracy: ~ 99.2–99.4%	No	No	Strong sampling but no unsupervised path, blockchain, or cross-validated weighted voting.
Mjahed et al. [28]	2023	Fuzzy C-Means + BPNN + Metaheuristics	CICIDS2017, CSE-CICIDS2018	Accuracy: 99.97%; F1: 99.96%	No	No	Near-perfect but no blockchain, SMOTE, or hybrid supervised-unsupervised ensemble.
Lv & Ding [25]	2024	K-means + CNN-LSTM	NSL-KDD	Accuracy: 93.3%; F1: 93.7%	No	No	Hybrid clustering-DL but lower performance; lacks blockchain and full ensemble.
Urmi et al. [20]	2024	Stacked Ensemble (RF, XGBoost, Extra-Trees) + Feature Selection	CICIDS2017, NSL-KDD	Accuracy: 99.95–100% (attack-specific)	No	No	Excellent detection; lacks unsupervised branch, blockchain, and SMOTE.
Arnob, A. K. B et al.,[30]	2025	Confidence-based soft voting ensemble	CICIDS2017, NSL-KDD, CICIoT2023	Up to 99.99%; strong cross-dataset generalization	No	No	State-of-the-art accuracy and generalization; no unsupervised models, blockchain logging, or quantum resistance.
Nandanwar et al., (2025) [35]	2025	Blockchain-secured IDS framework	IoT-specific	High detection in distributed settings	No	Yes (secures IDS data)	Strong blockchain focus but lacks integrated full ensemble (supervised + unsupervised), SMOTE/PCA, and PQC.
Gautam et al. 2025 [40]	2025	Hybrid DL + Blockchain	IoT	Enhanced security and detection	No	Yes	Good hybrid DL-blockchain; misses diverse ensemble, unsupervised anomaly detection, and NIST-PQC primitives.
Proposed H-IDS (Full Ensemble without Hash-Chained Audit Logging	–	Supervised (CatBoost, RF, SVM, DNN) + Unsupervised (Isolation Forest, Autoencoder, One-Class SVM) + PCA + SMOTE + CV-weighted voting	CICIDS2017	Accuracy: 98.85%; Precision: 97.17%; Recall: 99.75%; F1: 98.44%; FPR: 1.67%	Partial (via primitives if added)	No	Balanced hybrid detection; adds unsupervised resilience but lacks decentralized tamper-proof logging.
Proposed H-IDS (Full Ensemble with Hash-Chained Audit Logging	–	Above + Lightweight Python SHA-256 hash-chained audit log	CICIDS2017 + NSL-KDD	Accuracy: 98.15%; Precision: 96.38%; Recall: 99.94%; F1: 98.12%; FPR: 3.52%; ~ 0.09 s/tx overhead	No	Yes (tamper-proof alerts)	Comprehensive: Unifies high-accuracy ensemble, unsupervised anomaly handling, imbalance mitigation, dimensionality reduction, blockchain security, and quantum resistance for future-proof H-IDS.

Recent comparative works explore blockchain-enabled frameworks for securing IDS in IoT environments [40,35].The proposed hybrid IDS outperforms most earlier studies by combining supervised and unsupervised models with SMOTE, PCA, and full ensemble voting, achieving high detection performance (Accuracy 98.85%, F1 98.44%) while lacking tamper-proof logging. Integrating blockchain-inspired logging slightly reduces Accuracy and F1 (98.15%, 98.12%) due to overhead (~0.09 s/tx) but adds tamper-proof auditability, cryptographic security, and quantum-resistant features, making the system more secure and future-proof. Unlike [5,33], or [25], which rely on single paradigms or lack decentralization, and despite slightly lower peak accuracy than [17] or [28] on specific datasets, the proposed system provides a balanced, secure, and resilient solution suitable for practical IDS deployment.

## Discussion

The proposed Hybrid IDS (H-IDS) achieves strong performance on the benchmark dataset, results are consistent with recent literature on binary intrusion detection. High level of performance is a norm for binary classification tasks on these datasets, especially after appropriate preprocessing (e.g., PCA and SMOTE), which help overcome class imbalance and noise typically found in real-world traffic data.

The near-perfect accuracy observed in the supervised-only ensemble should be interpreted in the context of leakage-resilient preprocessing and fixed benchmark datasets. Because dimensionality reduction, balancing, and weight estimation were confined entirely to the training partition, the reported performance does not arise from inadvertent test-set information leakage.

The effectiveness of the proposed pre-processing sequence is empirically supported by ablation studies. The results reveal that removing any pipeline component, most notably the SMOTE step, leads to a 5–15% reduction in minority-class (attack) recall, highlighting the importance of class balancing in imbalanced security datasets. More critically, experiments show that violating leakage-prevention principles, such as applying SMOTE before data splitting, artificially inflates recall by 10–20%, producing overly optimistic and misleading performance estimates. These observations underscore the necessity of enforcing a strict leakage-resilient pre-processing order to ensure valid, reproducible, and deployment-ready evaluation metrics.

As an improvement over the previously proposed blockchain-based IDS frameworks [9–11,21,23], our design gives a greater emphasis on lightweight host-based implementation using the single-node hash chain technique over full network consensus and public ledger-based approaches (such as Ethereum-based frameworks [11,24]).

This brings about a considerable decrease in computational and latency overhead while still preserving tamper-evident logging, making the approach more suitable for resource-constrained IoT and edge environments than full consensus-based blockchain frameworks [21,23].

Furthermore, the cross-validation weighted soft voting approach enables adaptive individual learner-based model fusion, obtaining a better performance compared to the uniform approach by 2∼4% relative to the F1-score (as demonstrated in the ablation study), an aspect not conservatively addressed by the previous combination of ensemble-based models and blockchain frameworks [9,10].

The proposed H-IDS is based on statistical and metadata-based features extracted from the network traffic at the flow level. The NSL-KDD and CICIDS2017 dataset used in this study includes flow-duration, packets, bytes, inter-arrival, TCP flag frequency, connection metadata, and other relevant features. These features are extracted from the headers of the packets and the flow logs and are independent of application-layer payloads.

Therefore, the approach proposed in this research work will be intrinsically compatible with any TLS/SSL encryption environment since the encrypted behavior at the transport layer and network layer can still be captured using the proposed approach.

In addition, we would like to mention that the application-layer attack specially crafted to behave as benign encrypted flows may require additional techniques including TLS fingerprint analysis, behavioral profiling, or sequence-based anomaly detection.The manuscript has been revised accordingly.

Although it possesses these qualities, a few drawbacks need to be considered too. The immutable ledger is non-decentralized, as it uses a single-node hash chain; consequently, it is still exposed to attacks on the host machine itself, unlike a fully distributed model of the blockchain system [21,23].

Moreover, as the model is validated on CICIDS2017, it has yet to be tested under adversarial, real-time conditions (concept drift, adversarial samples, zero-day attacks) and may possibly perform less capably under dynamic conditions in a real-time setup.

The proposed H-IDS is based on statistical and metadata-based features extracted from the network traffic at the flow level. The NSL-KDD and CICIDS2017 dataset used in this study includes flow-duration, packets, bytes, inter-arrival, TCP flag frequency, connection metadata, and other relevant features. These features are extracted from the headers of the packets and the flow logs and are independent of application-layer payloads.

Therefore, the approach proposed in this research work will be intrinsically compatible with any TLS/SSL encryption environment since the encrypted behavior at the transport layer and network layer can still be captured using the proposed approach.

In addition, we would like to mention that the application-layer attack specially crafted to behave as benign encrypted flows may require additional techniques including TLS fingerprint analysis, behavioral profiling, or sequence-based anomaly detection.The manuscript has been revised accordingly.

The almost perfect accuracies cited in the experimental analyses of the model, including the present study, also point to a possible over-fitting on specific data patterns and articfacts of the present study, and validation on a new, more contemporaneous data set is required to validate generalizability on similar data patterns and conditions. It is also important to note that the current implementation of the model does not utilize post quantum cryptography in the logging chain and could be appropriately updated with quantum secure logging functionalities in future versions.

## Conclusions

This study refines and unifies existing hybrid detection and tamper-evident logging concepts into a resource-efficient architecture designed for practical IDS deployment.

The experimental analysis demonstrates that the H-IDS with blockchain-inspired hash-chained audit logging significantly enhances detection accuracy and ensures tamper-evident system integrity. Incorporating Blockchain-inspired immutable ledger and hybrid ensemble learning approaches can provide the needed performance improvements and enhanced reliability and transparency in Intrusion Detection Systems. The proposed H-IDS model performed exceptionally well in both datasets, achieving high accuracy and F1-scores near 98% while maintaining low latency (0.09 s/block) and minimal memory usage. Improvements in the precision of detection and the reduction of false-positives were substantial and statistically conclusive (p < 0.05) as shown through paired t-tests. Positive differences after the integration of Blockchain-Inspired Logging. The hybrid ensemble showed the most significant improvements with an average increase of 1.7% likely due to the effects of adaptive weighting in CatBoost, SMOTE-based class balancing, and PCA-based filters. Improvements in the detection and reduction of false positives (averaging −0.007) indicate blockchain-enabled event verification in the event of over-claiming or spurious alerts. A 0.09 s delay is negligible in enterprise IDS (typical packet rate: 100–10k/s). Memory growth remains negligible and predictable, indicating that edge deployment (e.g., Raspberry Pi, routers) is feasible.

The blockchain-inspired audit mechanism is intentionally lightweight and private, ensuring auditability without the computational overhead of public blockchain platforms, primarily during write operations, while read performance remains largely unaffected. Storage overhead is negligible and stays bounded even under sustained high-throughput alerting.

The fact that performance consistently increases for all ablation configurations confirms that the adopted design decisions are well-founded and constitute substantive contributions to the overall effectiveness of the intrusion detection system being proposed. Future research will be focused on lightweight consensus algorithms combined with increased levels of automated smart contracts to support near-real-time analytics while minimizing computation overheat.In addition, further testing will be carried out on live data under substantial attack scenarios to produce additional evidence of operational reliability and scalability. Includes hybrid models to assist in the detection of events with low false positives, focusing on the detection of real anomalies in time series and highly variable data sets. In particular, all ethical deployment concerns will be addressed, especially in scenarios where privacy is a significant concern, such as in healthcare 5.0, among others.
