# Supplementary material for: A lightweight blockchain-inspired hybrid intrusion detection system with ensemble learning for tamper-proof auditing
Source: PLoS One. 2026 Sep 11;21(9):e0356878. doi: 10.1371/journal.pone.0356878 (PMC13567761; doi:10.1371/journal.pone.0356878)
Supplement: Supplementary file 2 [file pone.0356878.s003.docx]

**Response to Reviewers** Manuscript ID: PONE-D-25-59640R1

**Title:** A Lightweight Blockchain-Inspired Hybrid Intrusion Detection System with Ensemble Learning for Tamper-Proof Auditing

Dear Dr. Muhammad Anwar and Reviewers,

Greetings!

We sincerely thank both reviewers for their thorough evaluation of the revised manuscript and for the constructive final comments. We are pleased that both Reviewer #1 and Reviewer #2 now consider the major scientific and technical concerns from the previous round to have been adequately addressed, and that the work is viewed as technically sound, well-supported, clearly presented, and suitable for publication in PLOS ONE.

We have carefully considered the two *minor suggestions for final polish* raised by Reviewer #2 and implemented the corresponding improvements in the submitted files:

**Reviewer #2 – Comment 6.1 – Consistency Check**

**“While the authors state in the "Response to Reviewers" that storage overhead is corrected to "negligible," they should ensure that any remaining "0 KB" or "zero persistent growth" phrases in old text blocks (if any) are purged during the final proofing stage.”**

**Response:** Thank you for this careful observation. We performed a complete keyword search and manual proof-reading pass across the entire manuscript to identify and eliminate any remaining absolute-zero phrasing. All instances of “0 KB”, “zero persistent growth”, “zero memory growth”, and similar expressions have been replaced with technically accurate and consistent terminology such as “negligible”, “minimal”, or “very low and predictable”.

The most relevant corrected passages now read (highlighted in **red text** in the “Revised Manuscript with Track Changes” file):

- **Conclusions section –** performance summary paragraph (lines ≈940–944 in the tracked-changes document): “A 0.09 s delay is negligible in enterprise IDS (typical packet rate: 100–10k/s). Memory growth remains negligible and predictable, indicating that edge deployment (e.g., Raspberry Pi, routers) is feasible.”
- **Conclusions section** – audit mechanism description (lines ≈945–949): “The blockchain-inspired audit mechanism is intentionally lightweight and private, ensuring auditability without the computational overhead of public blockchain platforms, primarily during write operations, while read performance remains largely unaffected. Storage overhead is negligible and stays bounded even under sustained high-throughput alerting.”

These revisions ensure uniform, conservative, and evidence-aligned language throughout the manuscript.

**Reviewer #2 – Comment 6.2**

**Novelty Positioning: Ensure the "softened" novelty claims (acknowledging similar hybrid models) are consistently applied in both the Introduction and Conclusion to avoid contradictions.**

**Response:** We appreciate this valuable suggestion. We have further aligned the tone and phrasing concerning novelty/contribution across the major sections (Introduction, Discussion, and Conclusion) to consistently present our work as a meaningful refinement, unification, and practical integration of existing hybrid detection and lightweight tamper-evident logging concepts ,rather than claiming wholly novel components.

Key updated passages (highlighted in **red text** in the tracked-changes file) include:

- **Introduction –** contribution positioning paragraph (lines ≈84–87): “While prior studies have explored hybrid intrusion detection models and blockchain-assisted logging independently, fewer works have examined a tightly integrated, lightweight hash-chained audit mechanism combined with supervised–unsupervised ensemble learning under a unified evaluation framework.”
- **Discussion section –** blockchain comparison paragraph (lines ≈899–902): “This brings about a considerable decrease in computational and latency overhead while still preserving tamper-evident logging, making the approach more suitable for resource-constrained IoT and edge environments than full consensus-based blockchain frameworks.”
- **Conclusion section –** contribution summary paragraph (lines ≈924–925): “This study refines and unifies existing hybrid detection and tamper-evident logging concepts into a resource-efficient architecture designed for practical IDS deployment.”

We believe these final adjustments fully resolve the two minor points raised. No other changes were made beyond these targeted consistency improvements.

We confirm that:

- All data underlying the findings are available (code repository link and processed dataset splits are provided in the manuscript).
- The reference list has been checked for completeness and correctness; no retracted articles are cited.
- The marked-up (“Revised Manuscript with Track Changes”) file shows all changes since the previous submission, with the two sets of minor wording updates highlighted in **red text** for easy identification.

We are grateful for the detailed and constructive feedback throughout the review process, which has substantially improved the clarity, rigor, and scientific integrity of the manuscript.

Thank you again for considering our revised submission. We look forward to your final decision.

Sincerely,

Shailendra Mishra

Corresponding Author

(on behalf of all authors)

Department of Computer Engineering

College of Computer and Information Sciences

Majmaah University, Saudi Arabia
