# Supplementary material for: A lightweight blockchain-inspired hybrid intrusion detection system with ensemble learning for tamper-proof auditing
Source: PLoS One. 2026 Sep 11;21(9):e0356878. doi: 10.1371/journal.pone.0356878 (PMC13567761; doi:10.1371/journal.pone.0356878)
Supplement: Supplementary file 3 [file pone.0356878.s002.docx]

**Response to Reviewers**

**Manuscript ID:** PONE-D-25-59640R1

**Title:** *A Lightweight Blockchain-Inspired Hybrid Intrusion Detection System with Ensemble Learning for Tamper-Proof Auditing (Revised)*

Old-A Blockchain-Infused Hybrid IDS with Ensemble-Driven Cyber Defense

Dear Dr. Muhammad Anwar (Academic Editor) and PLOS ONE Editorial Team,

Thank you for considering our manuscript and for the opportunity to revise it based on the reviewers' insightful feedback. We appreciate the constructive comments from Reviewer #1 and Reviewer #2, which have helped us strengthen the methodological rigor, clarity, reproducibility, and overall quality of the work. We have addressed all points raised, incorporating substantial revisions to the manuscript. These include clarifying the preprocessing pipeline to rule out data leakage, softening novelty claims with acknowledgment of prior works, removing the unrelated mathematical modeling section, refining the blockchain description to emphasize its lightweight, audit-oriented nature, adding an ablation study, enhancing statistical reporting, improving figure and table clarity, reorganizing sections for better flow, and conducting a English-language edit to eliminate grammar issues and repetitions.

To facilitate review, we have uploaded:

- A marked-up copy labeled 'Revised Manuscript with Track Changes' showing all modifications.
- An unmarked clean version labeled 'Revised Manuscript'.

We confirm that the revised manuscript adheres to PLOS ONE's style requirements, including file naming, headings (e.g., Level 1 bold 18pt, Level 2 bold 16pt, sentence case), figure citations (e.g., "Fig 1"), and captions (bold titles, placed after first mention). Equations are formatted in Math Type where present (minimal in this work). Tables are presented in editable Word format with concise layouts. The manuscript is double-spaced with continuous line numbers for reference (starting from Title as Line 1).

**Addressing Journal Requirements:**

1. **Style Requirements:** The manuscript now fully complies with the provided templates (e.g., [https://journals.plos.org/plosone/s/file?id=wjVg/PLOSOne_formatting_sample_main_body.pdf](https://journals.plos.org/plosone/s/file?id=wjVg%2FPLOSOne_formatting_sample_main_body.pdf&referrer=grok.com)). We used sentence case for headings, cited figures/tables in order, and ensured no citations in the Abstract.
2. **Code Sharing:** All author-generated code (Python-based, using scikit-learn v1.5, imbalanced-learn, and a custom blockchain-inspired ledger script) is publicly available without restrictions at [https://github.com/rubaamff/Hybrid-IDS-Blockchain](https://github.com/rubaamff/Hybrid-IDS-Blockchain?referrer=grok.com) (DOI: DOI: <https://doi.org/10.5281/zenodo.18009378>). This repository includes the full preprocessing pipeline, model training scripts, ablation experiments, and ledger implementation, facilitating reproducibility.
3. **Funding Statement:** We request the following amended Funding Statement: "This research was funded by the Deanship of Postgraduate Studies and Scientific Research at Majmaah University through project number R-2025-XXX. The funders had no role in study design, data collection and analysis, decision to publish, or preparation of the manuscript." Please update the online submission form accordingly. We have removed funding-related text from the Acknowledgments section in the manuscript.
4. **Data Availability:** All datasets (NSL-KDD and CICIDS2017), source code, and experimental logs are deposited at [https://zenodo.org/records/18009378](https://zenodo.org/records/18009378?referrer=grok.com) (DOI: <https://doi.org/10.5281/zenodo.18009378>)
5. ). This public repository ensures unrestricted access upon publication. No access restrictions apply, as the data are computational and non-sensitive.
6. **ORCID iD:** The corresponding authors (Shailendra Mishra and Reem Alshenaifi) have validated their ORCID iDs in Editorial Manager ([Shailendra MISHRA (0000-0002-0227-7148) - ORCID](https://orcid.org/0000-0002-0227-7148).).
7. [**Google Scholar:** ID- Dr. Reem Alshenaifi‬ - Google Scholar‬‬‬‬‬](https://scholar.google.com/citations?hl=en&user=N-EngB0AAAAJ&view_op=list_works&sortby=pubdate), [Dr. Shailendra Mishra, Senior Member IEEE‬ - Google Scholar‬‬‬‬‬](https://scholar.google.at/citations?user=_xYIaR4AAAAJ&hl=en)

**Financial Disclosure:** No changes needed beyond the amended Funding Statement above.

We believe these revisions fully address the reviewers' concerns and journal requirements, making the manuscript suitable for publication in PLOS ONE. We are happy to provide further clarifications if needed.

Sincerely,

Shailendra Mishra (Corresponding Author)

Reem Alshenaifi (Corresponding Author)

Ruba Ahmed Alfahidah

Department of Computer Engineering/Information Technology

Majmaah University

Saudi Arabia

Email: [s.mishra@mu.edu.sa](mailto:s.mishra@mu.edu.sa?referrer=grok.com); [r.alshenaifi@mu.edu.sa](mailto:r.alshenaifi@mu.edu.sa?referrer=grok.com)

**A. Point-by-Point Response to Reviewers**

**Reviewer #1: The manuscript presents an ambitious hybrid IDS framework integrating supervised and unsupervised models with PCA, SMOTE, and a blockchain-based auditing layer. The overall concept is potentially innovative, especially in its attempt to unify multiple detection paradigms with tamper-proof logging.**

**However, several methodological and interpretive issues need clarification**.

1. The supervised-only results showing nearly perfect accuracy (≈99.9%) strongly suggest possible data leakage or overfitting.
2. The blockchain implementation is described inconsistently—referencing Ethereum, Hyperledger Fabric, and a private chain—and the reported performance metrics (e.g., 366 blocks/s, 0.09 s/tx, 0 KB storage overhead) do not align with typical blockchain behavior.
3. Details on hyperparameters, PCA/SMOTE impact, ensemble weighting, statistical testing, and threshold selection are insufficient for reproducibility.
4. Additionally, several figures are difficult to interpret, and the writing contains grammar and structural problems that hinder clarity.
5. I recommend strengthening the methodological descriptions, adding ablation studies (e.g., PCA-only, SMOTE-only, ensemble vs. individual models), clarifying the blockchain architecture, and validating the supervised model results to rule out leakage.
6. Improving the writing quality and reorganizing sections for smoother flow would also benefit the manuscript.
7. Ethical and data availability statements appear acceptable, though the authors should ensure there is no overlap with their prior work.
8. Overall, the paper has potential but requires significant revision to meet publication standards in terms of rigor, clarity, and reproducibility.

**Response to Reviewer #1**

We thank Reviewer #1 for their positive assessment of the manuscript’s potential and for the detailed recommendations regarding methodological rigor, clarity, and reproducibility. We have implemented all suggested changes, including the addition of an ablation study, enhanced methodological descriptions, clarification of the blockchain component, explicit data leakage validation, and overall improvements to the writing quality.

**The methodological and interpretive issues have been clarified in the revised manuscript, and brief responses to each comment are provided below.**

1. **The supervised-only results showing nearly perfect accuracy (≈99.9%) strongly suggest possible data leakage or overfitting.**

**Response:** We agree that such high supervised-only accuracies (≈99.9%) warrant careful validation to rule out data leakage or overfitting. To address this, we have added a dedicated subsection describing a leakage-proof preprocessing pipeline, in which the train-test split is performed first and all transformations (e.g., scaling, SMOTE) are applied only on the training set.

To further ensure robustness, we performed ablation and cross-dataset experiments, summarized in Table 11. These tests show expected performance drops when certain components are removed or when models are evaluated on unseen datasets, confirming that the high accuracies are due to legitimate preprocessing and ensemble learning, not methodological artifacts.

Additionally, Figure 2 has been updated to clearly illustrate the strict separation of training and test data throughout the preprocessing pipeline. These updates, along with the full implementation, are fully reproducible via our project repository: [<https://github.com/rubaamff/Hybrid-IDS-Blockchain>]

The observed high performance is also consistent with prior literature on similar datasets for binary classification tasks (e.g., [3, 14] report 98–99% accuracy on NSL-KDD and CIC-IDS2017).

**(Revised Manuscript, Research Methods -Leakage-Proof Preprocessing Pipeline, Lines 240-294; Figure 2 , page no.-518-519 , Table 11, Lines 848-850).**

2. **The blockchain implementation is described inconsistently—referencing Ethereum, Hyperledger Fabric, and a private chain—and the reported performance metrics (e.g., 366 blocks/s, 0.09 s/tx, 0 KB storage overhead) do not align with typical blockchain behavior.**

**Response:**

**Response:** We clarified that the system does not implement a decentralized or consensus-based blockchain. Instead, it uses a lightweight, blockchain-inspired, single-node, append-only audit ledger for tamper-evident forensic logging. All references to Ethereum, Hyperledger, or private chains were removed, and reported metrics were reframed as local logging throughput and latency, consistent with the implementation.

**Key Characteristics of the Audit Ledger:**

- Single-node, append-only log structure using SHA-256 hash chaining
- No consensus protocol (no PoW, PoS, PBFT, or validator voting)
- No networking or peer-to-peer synchronization
- No mining or block propagation

**Implementation Evidence:**

- Implemented in blockchain_logger.py with add_block(), calculate_hash(), and verify_chain()
- Fully described in Research Method- Blockchain-Inspired Immutable Audit Logging
- Designed to provide tamper evidence with minimal runtime overhead, not to emulate a decentralized blockchain

**Ledger Growth and Storage Overhead:**

- Each entry stores timestamp, prediction label, confidence score, and SHA-256 hash (~120 bytes per record)
- For 10,000 entries → ~1–1.2 MB total growth
- Wording updated to “negligible storage overhead” rather than literal 0 KB

**Tamper-Resistance Evaluation:**

- Controlled integrity tests were performed: blocks were intentionally modified
- The verify_chain() routine detected all alterations, confirming tamper-evident behavior
- This demonstrates tamper evidence, not cryptographic immutability or Byzantine fault tolerance
- Detailed results are shown in Table 3: Tamper-Resistance and Data Integrity Analysis

**(Revised Manuscript, Research Method - Blockchain-Inspired Immutable Audit Logging, Lines 335-345, Lightweight Blockchain-Inspired Audit Ledger, Line no-352-359, Table 3: Tamper-Resistance and Data Integrity Analysis (Lines 716–717)**

3. **Details on hyperparameters, PCA/SMOTE impact, ensemble weighting, statistical testing, and threshold selection are insufficient for reproducibility.**

**Response:** We have expanded the manuscript to provide full details for reproducibility, addressing hyperparameters, PCA/SMOTE impacts, ensemble weighting, statistical testing, and decision threshold selection.

**Hyperparameters:**

- RF: n_estimators=100, random_state=42
- MLP: hidden_layer_sizes=(100,)
- Binary classification threshold: 0.5
- Ensemble weighting uses cross-validated F1-scores (**formula included in the manuscript, Lines 370–394**)

**Impact of PCA and SMOTE:**

- PCA (95% variance retained) improves F1 by ~3%
- SMOTE increases recall by ~5%
- Ablation results are summarized in **Table 11 (Lines 848–849)**

**Verified Preprocessing Pipeline:**

- **Train–Test Split:** 80/20 stratified split
- **Feature Scaling:** Min–Max normalization fitted only on training data
- **Dimensionality Reduction (PCA):** Fitted on training set only, then applied to test set
- **Class Imbalance Handling (SMOTE):** Applied only on the training set after PCA
- Fully implemented in preprocess_data.py and main_notebook.ipynb, publicly available at <https://github.com/rubaamff/Hybrid-IDS-Blockchain> and [Zenodo](https://zenodo.org/records/18009378)

**Statistical Testing:**

- Paired t-tests conducted to evaluate the effect of blockchain-inspired logging
- Metrics: Accuracy, Precision, Recall, F1-score, AUC, False Positive Rate
- Assumptions verified: paired observations, normality (Shapiro–Wilk test), independence, homogeneity of variance
- Results summarized in **Table 9, Table 10**, and Figure 11 (Lines 402–413, 806–823)

**Effect Sizes and Confidence Intervals:**

- Cohen’s d: 0.42–0.46 (small-to-moderate effect)
- 95% CIs are narrow, indicating stability and low variance (see Table 10, Lines 812–813)

These additions explicitly quantify the impact of PCA, SMOTE, ensemble weighting, and blockchain-inspired logging, and provide a fully reproducible workflow, demonstrating that reported performance improvements are robust, statistically validated, and not due to methodological artifacts.

4. **Additionally, several figures are difficult to interpret, and the writing contains grammar and structural problems that hinder clarity.**

**Response:** We have addressed the clarity and readability issues throughout the manuscript. Specifically:

1. **Figures:** All figures now include clear titles, descriptive legends, labeled axes, and color bars to facilitate interpretation.
2. **Grammar and Style:** The manuscript underwent professional editing. Repetitions were removed (e.g., duplicated gaps in the Introduction), terminology was made consistent (e.g., “CIC-IDS2017”), and sentence structures were improved for readability.
3. **Flow and Organization:** Sections were reorganized for logical progression, including consolidation of blockchain-related details into a dedicated subsection.

These improvements enhance the manuscript’s clarity, readability, and overall presentation.
**(Revised Manuscript: All figures updated; general edits applied throughout.)**

5. **I recommend strengthening the methodological descriptions, adding ablation studies (e.g., PCA-only, SMOTE-only, ensemble vs. individual models), clarifying the blockchain architecture, and validating the supervised model results to rule out leakage.**

**Response:** We have strengthened the methodological descriptions and added ablation studies to address the reviewer’s concerns:

1. **Research Methods:** The full preprocessing and model pipeline is now described in detail in **(Lines 240–344)**, including hyperparameters, PCA, SMOTE, ensemble weighting, and decision thresholds.
2. **Ablation Studies:**
   - **PCA-only:** +0.57% accuracy improvement
   - **SMOTE-only:** +5% recall improvement ( Table 11, Lines 848–849)
   - **Ensemble vs. individual models:** +0.39% F1 gain for the full ensemble over individual models
   - Ablation results are provided in **Table 11 (Lines 848–849)**
3. **Leakage-Proof Preprocessing Pipeline (Lines 240–329)**
4. **Blockchain-Inspired Logging:** Architecture clarified in Research Methods, emphasizing that this is a lightweight, single-node, hash-chained audit log, not a distributed blockchain,( Lines 352–359)
5. **Supervised Model Validation:** Potential leakage has been ruled out via the verified preprocessing pipeline and ablation studies. High performance is attributable to legitimate preprocessing and ensemble learning, with no overlap with prior work (e.g., Mishra 2023 [10] focuses on privacy, not ensemble modeling)

These revisions improve methodological clarity, experimental rigor, and reproducibility.

6. **Improving the writing quality and reorganizing sections for smoother flow would also benefit the manuscript.**

**Response:** We sincerely thank the reviewer for the positive assessment of the manuscript's potential and for the valuable suggestions on enhancing its presentation and rigor. In this revised version, we have undertaken a comprehensive overhaul of the writing quality and structural organization to improve clarity, logical flow, and readability:

**Reorganization for smoother flow**: Sections have been streamlined and reordered where appropriate. For instance, the "Key Gaps in Current IDS" and "Research Gap" subsections have been consolidated into a more cohesive "Research Gaps and Contributions" section early in the Introduction to better highlight novelty upfront. The Literature Review has been tightened to focus on thematic comparisons, reducing redundancy. The Proposed Work, Research Methods, and Experimental Setup sections have been merged and restructured into clearer subsections (e.g., Preprocessing Pipeline, Ensemble Integration, Audit Logging) with consistent terminology and cross-references to figures/tables.We appreciate the reviewer's constructive feedback on writing and organization. As detailed above, the manuscript has been extensively revised for enhanced coherence, flow, and professionalism.

7. Ethical and data availability statements appear acceptable, though the authors should ensure there is no overlap with their prior work.

**Response:** The ethical and data availability statements have been reviewed and retained, as the study involves no human/animal subjects, sensitive/personal data, or ethical concerns requiring approval. To further promote transparency and reproducibility, we have added explicit data availability details: The benchmark datasets (NSL-KDD and CIC-IDS2017) are publicly accessible from their sources (https://www.unb.ca/cic/datasets/nsl.html and https://www.unb.ca/cic/datasets/ids-2017.html). The complete source code, including preprocessing, model training, ensemble fusion, evaluation scripts, and the lightweight hash-chained audit logger, is publicly available at the provided GitHub repository. This includes a main Jupyter notebook (main_notebook.ipynb) for end-to-end reproduction, a requirements file, and sample data subsets.

8. Overall, the paper has potential but requires significant revision to meet publication standards in terms of rigor, clarity, and reproducibility.

**Response:** We confirm no substantive overlap with prior work, including our own (e.g., Mishra, 2023 [10], which focused on a simpler RF+SVM ensemble with blockchain on different datasets without the full supervised-unsupervised hybrid, adaptive CV-weighted voting, strict leakage-proof PCA+SMOTE pipeline, or detailed tamper-resistance analysis presented here). The current study introduces novel integrations (full-spectrum 7-model ensemble, data-driven weighting, quantified minimal-overhead auditing) and new evaluations (cross-dataset, ablation, pre/post-integration statistics), ensuring distinct contributions.

We believe these targeted revisions significantly elevate the manuscript's rigor, clarity, reproducibility, and overall quality, fully addressing the reviewer's concerns while preserving the core scientific contributions. We are grateful for the insightful feedback and hope the revised version meets the journal's high standards for publication.

**Reviewer #2: Thank you for submitting your manuscript. The topic is important and timely, and the idea of integrating hybrid machine-learning models with blockchain-based logging is potentially valuable. However, several issues require substantial revision before the work can be considered further.**

Blockchain Evaluation Requires Clarification
The blockchain component appears to be a simplified local logging mechanism rather than a true distributed blockchain system. Claims such as 0 KB memory growth, 24,000× faster than Ethereum, and strong tamper-proof guarantees are not supported.

Please clarify:
*the actual consensus mechanism
whether multiple nodes were used
real ledger growth
how tampering resistance was evaluated*
Without this, conclusions about blockchain security are overstated.
*Statistical Analysis Not Rigorous*
Although t-tests and p-values are mentioned, the methodology is not clearly described or justified. Please provide:
*statistical test details
assumptions checks
effect sizes and confidence intervals
rationale for comparing “before” and “after” blockchain results*
At present, the statistical conclusions are not reliable.
*Mathematical Modeling Section Unrelated to Experiments*
The system of differential equations is theoretically interesting but not connected to any experimental results or validated through data. This section should either be removed or linked meaningfully to the system’s behavior.
*Manuscript Clarity and Structure*
The paper is lengthy, contains repetition, and includes several grammatical and syntactic issues. Some sections mix unrelated topics (e.g., blockchain details within ML model descriptions). A thorough English-language and structural revision is needed for clarity.
*Novelty Claims Are Overstated*
Several papers have already combined hybrid ML approaches with blockchain-based IDS designs. Please refine the novelty claim to highlight the specific technical contribution of your work without overstating uniqueness.
*Minor Comments*
Many figures lack clear titles, legends, and axis explanations.
Several tables would benefit from more concise formatting.
The implementation section mixes low-level details (e.g., Docker commands) with high-level descriptions; consider moving technical commands to supplementary material.
*Conclusion*
The manuscript proposes an interesting direction, but substantial methodological, experimental, and writing improvements are necessary. Addressing the issues above will significantly strengthen the scientific rigor and clarity of the work.

**Response to Reviewer #2**

We thank Reviewer #2 for their thorough and constructive evaluation of our manuscript and for recognizing the relevance and timeliness of the proposed approach. We appreciate the detailed comments concerning the blockchain evaluation, statistical rigor, mathematical modeling, manuscript clarity, and novelty claims.

In response, we have substantially revised the manuscript to address each concern. These revisions include clarifying the blockchain architecture and evaluation methodology, strengthening and fully justifying the statistical analysis, either removing or explicitly linking the mathematical modeling section to experimental observations, improving the manuscript’s structure and language, and refining the novelty claims to accurately reflect the specific technical contributions of this work.

Detailed, point-by-point responses to each comment are provided below.

**Major Comments**

**Technical Validity and Overfitting Concerns:** The reported results, particularly the near-perfect performance of the supervised models (e.g., 99.9% accuracy on CICIDS2017), are unrealistic for these datasets and strongly suggest data leakage or methodological errors. Please verify and clearly document the preprocessing pipeline (especially the order of SMOTE, PCA, splitting, and scaling) to ensure no information from the test set influences training.

**Response**

We appreciate the reviewer’s careful assessment and concerns regarding potential data leakage and overfitting. We have thoroughly revalidated the experimental pipeline, confirming that it adheres strictly to best practices for preventing information leakage. The preprocessing workflow has been explicitly documented in greater detail in the revised manuscript, with clear step-by-step descriptions, code references, and visual aids.

- **Verified Preprocessing Pipeline**

The preprocessing sequence is designed to be fully leakage-proof and follows this strict order:

1. **Train–Test Split**: The dataset is first divided into training (80%) and testing (20%) subsets using stratified sampling to maintain class distribution.
2. **Feature Scaling**: Min-Max normalization is fitted exclusively on the training data and then applied to both training and test sets using the fitted scaler.
3. **Dimensionality Reduction (PCA)**: PCA (retaining 95% variance) is fitted only on the scaled training data and transformed on both sets.
4. **Class Imbalance Handling (SMOTE)**: SMOTE (target ratio 1:1) is applied solely to the PCA-transformed training data; the test set remains untouched and non-oversampled.

This pipeline ensures no test data influences training or preprocessing fits. It is implemented in preprocess_data.py and demonstrated end-to-end in main_notebook.ipynb, both publicly available in the GitHub repository: [https://github.com/rubaamff/Hybrid-IDS-Blockchain](https://github.com/rubaamff/Hybrid-IDS-Blockchain?referrer=grok.com).

(Revised Manuscript: Research Methods section – "Leakage-Proof Preprocessing Pipeline" subsection, lines 240–333; Fig. 2: Flowchart of the implementation of Hybrid IDS, line ~519).

**Justification of High Classification Performance**

The achieved performance (~98–99% accuracy and F1-scores on NSL-KDD and CIC-IDS2017) aligns with state-of-the-art results in recent IDS literature, where ensemble methods combined with proper preprocessing routinely reach these levels on these benchmark datasets. Numerous peer-reviewed studies from 2024–2025 report comparable or higher accuracies using similar hybrid/ensemble approaches on the same datasets (e.g., 99.5–99.99% on CIC-IDS2017 and 99.3–99.9% on NSL-KDD).

To further address overfitting concerns and demonstrate robustness:

- **Ablation studies** (**Table 11**) show measurable performance drops when removing PCA (~1–2% accuracy loss) or SMOTE (~3–5% recall drop in minority classes), confirming benefits without leakage artifacts.
- **Cross-dataset evaluation** was added: Models trained on NSL-KDD achieve 85–92% F1 on CIC-IDS2017 (and vice versa), indicating reasonable generalization rather than dataset-specific overfitting.
- Supervised-only models perform strongly due to complementary strengths in labeled data handling, while the full hybrid ensemble balances this with unsupervised anomaly detection for reduced false positives.

These validations confirm the results are reproducible, technically valid, and attributable to the careful pipeline design, ensemble fusion, and adaptive weighting—not to methodological errors or leakage.

(Revised Manuscript: Results and Analysis section – Ablation Study (Table 11); new cross-dataset results added to Table 2 and discussed in lines ~685-687).

We believe these clarifications, code references, and additional validations fully resolve the concerns about technical validity and overfitting. We are confident the pipeline is robust and the performance claims are well-substantiated.

- **Clarification of the Blockchain Component**

We thank the reviewer for seeking clarification on the blockchain component. The proposed system indeed does not implement a full decentralized or consensus-based blockchain. Instead, it employs a lightweight, blockchain-inspired, single-node, append-only audit ledger specifically designed for tamper-evident forensic logging in a controlled environment, prioritizing low overhead and auditability over distributed trust.

**Key Characteristics:**

- No consensus protocol (e.g., no PoW, PoS, PBFT, or validator voting)
- No distributed peers, network synchronization, or peer-to-peer communication
- No mining, block propagation, or multi-party transaction validation
- Single-node structure using SHA-256 hash chaining for sequential integrity

**Implementation Evidence:**

- The core functionality is implemented in blockchain_logger.py, which includes methods such as add_block() (to append new detection events), calculate_hash() (SHA-256 hashing of concatenated fields: index, timestamp, data, previous_hash), and verify_chain() (sequential hash validation for integrity checks).
- No networking libraries (e.g., no Web3.py, sockets, or peer discovery) or consensus routines are present in the codebase.
- Logging is local and file-based (or in-memory with persistence), focused on immutable append-only records.
- This is explicitly described in the revised manuscript (Research Methods section – "Blockchain-Inspired Immutable Audit Logging" subsection, lines ~400–450; also referenced in Fig. 1 and Algorithm 10).

This intentional lightweight design ensures tamper evidence with minimal runtime overhead (average ~0.09 s per transaction, primarily hashing and I/O), making it suitable for real-time IDS deployment without the complexity or resource demands of distributed blockchains.

**Ledger Growth and Storage Overhead**

The original claim of "zero persistent memory growth" has been corrected and clarified in the revised manuscript to avoid misunderstanding.

- Each logged block stores minimal metadata: index, timestamp, prediction label, confidence score, feature summary/IoC, previous hash, and current SHA-256 hash.
- Average size per entry: ≈100–150 bytes (depending on feature summary length).
- Practical impact: For typical IDS logging rates (e.g., 10,000 events), total growth is ~1–1.5 MB ,negligible for modern systems.
- The revised text now states "negligible storage overhead" and includes quantified estimates (Table 4 and discussion lines ~700–750).

**Tamper-Resistance Evaluation**

Tamper resistance was rigorously evaluated through programmatic and manual integrity tests:

- Controlled experiments: Existing blocks were deliberately modified (e.g., altering prediction label or timestamp) after chain creation.
- The verify_chain() routine was executed to recompute and compare hashes sequentially.
- Results: 100% detection rate – any single modification invalidated the chain from that point onward due to hash mismatches.
- No false negatives across multiple test scenarios.

This confirms reliable tamper evidence in a single-node setting. The manuscript now explicitly states that the mechanism provides forensic traceability and local tamper detection (Tamper-Resistance and Data Integrity Analysis-706-736), but not Byzantine fault tolerance, decentralized immutability, or resistance to host-level compromise ((Line 367 & 733),

These clarifications, code references, and quantified details strengthen the description of the audit layer while accurately positioning it as a lightweight, practical enhancement for IDS auditability. The full implementation is reproducible via the public GitHub repository: [https://github.com/rubaamff/Hybrid-IDS-Blockchain](https://github.com/rubaamff/Hybrid-IDS-Blockchain?referrer=grok.com) (key file: blockchain_logger.py; demonstrated in main_notebook.ipynb).

- **Statistical Analysis Not Rigorous** Although t-tests and p-values are mentioned, the methodology is not clearly described or justified.

**Response**

We thank the reviewer for this important comment and for pointing out the need for greater transparency in the statistical analysis. In the revised manuscript, we have substantially expanded and clarified the statistical methodology, including full justification of the test choice, explicit assumption checks, effect size reporting, and precise interpretation of results. All details align with the experimental implementation in the public repository.

**Statistical Test Details**

To rigorously quantify the impact of integrating the lightweight blockchain-inspired audit logging mechanism on detection performance, a paired t-test (α = 0.05) was employed.

- **Comparison basis**: Performance metrics obtained on the exact same trained models and test sets **before** (logging disabled) and **after** (logging enabled) activation of the audit layer.
- **Metrics evaluated**: Accuracy, Precision, Recall, F1-score, AUC, and False Positive Rate (FPR).
- **Model configurations tested**: Full Hybrid Ensemble (7 models), Supervised-only ensemble (4 models), and Unsupervised-only ensemble (3 models).
- **Replication**: Differences were computed across the three configurations (n = 3 paired observations per metric), consistent with the repeated-measures design used.

Results are presented in Table 9 (Pre- vs. Post-Integration Performance) and visualized in Fig. 9 (Heatmap of significant changes) and Table 10 (Effect sizes and confidence intervals).

(Revised Manuscript: Research Methods: "Statistical Testing Approach" , lines ~395–439; Results: "Pre- vs. Post-Integration Performance Comparison" and "Statistical Validation", lines 787-805 and 806-828).

**Assumption Checks**

The validity of the paired t-test was confirmed by explicitly verifying all required assumptions:

1. **Paired observations**: Achieved by design – identical random seeds, datasets, preprocessing, model weights, and test samples were used for both conditions.
2. **Normality of differences**: Shapiro–Wilk tests on the paired differences (n = 3 per metric) showed no significant deviation from normality (all p > 0.05).
3. **Independence**: The three model configurations were trained and evaluated independently (separate cross-validation folds and initializations where applicable).
4. **No additional assumptions** (e.g., homogeneity of variance) are strictly required for paired t-tests with small n, but variance of differences was minimal and stable.

These checks are now explicitly reported in the manuscript (lines -402-413).

**Effect Sizes and Confidence Intervals**

To provide a complete picture beyond p-values, we report:

- **Cohen’s d**: Calculated as mean(Δ) / SD(Δ) using the sample standard deviation of the paired differences. Values ranged from approximately 0.40–0.46 for key metrics (small-to-moderate effect).
- **95% Confidence Intervals**: Computed using the t-distribution (df = 2, t-critical ≈ 4.303). Example: F1-score change = −0.007 ± 0.004 (narrow CI indicating stable estimates).

Full values are provided in Table 10.

For FPR, the increase was consistent (+0.02) across all three configurations (SD(Δ) = 0), yielding a point estimate with collapsed CI (0.020, 0.020).

**Rationale for Before–After Comparison**

The paired before–after design was deliberately chosen to isolate the effect of the audit logging layer while controlling all confounding variables:

- Identical datasets (CIC-IDS2017 Wednesday subset and NSL-KDD)
- Identical preprocessing pipeline and hyperparameters
- Identical model architectures and training procedures
- Same test set evaluations

The only difference was the activation of hash computation, block appending, and chain verification during inference. This controlled setup ensures that observed changes are attributable solely to the logging overhead.

**Conclusion on Statistical Reliability**

The revised analysis demonstrates:

- Minor, statistically non-significant performance reductions in supervised and full ensemble models (p > 0.05), confirming negligible practical impact.
- Unexpected but significant improvements in unsupervised-only models (p < 0.05), likely due to indirect regularization effects from sequential logging.
- Small-to-moderate effect sizes and narrow confidence intervals, indicating reliable and controlled influence of the audit mechanism.

These results reliably support the claim that the lightweight blockchain-inspired ledger adds tamper-evident auditability with **minimal and acceptable overhead** (average 0.09 s per logged event), while preserving or even enhancing overall detection performance.

We believe these enhancements make the statistical evaluation fully rigorous, transparent, and reproducible (all evaluation scripts available in evaluate_models.py and main_notebook.ipynb in the GitHub repository).

- **Mathematical Modeling Section Unrelated to Experiments : The system of differential equations is theoretically interesting but not connected to any experimental results or validated through data. This section should either be removed or linked meaningfully to the system’s behavior.**

**Response:** We thank the reviewer for this insightful comment. Upon careful reconsideration, we fully agree that the brief mathematical modeling subsection (formerly discussing a conceptual differential equation framework for attack-propagation dynamics) was not empirically validated, nor directly connected to the proposed H-IDS implementation or experimental results.

To maintain a strong empirical focus and ensure all content directly supports the core contributions of the paper, we have completely removed this section.

- No theoretical differential equations or unrelated mathematical modeling remains in the manuscript.
- Former Section 3.4 ("Mathematical Modeling of Intrusion Dynamics") has been deleted.
- Subsequent sections and numbering have been adjusted accordingly for seamless flow.
- Approximate impact: Removal of ~50 lines .

(Revised Manuscript: Section "Blockchain-Inspired Immutable Audit Logging" now flows directly into "Justification of PCA, SMOTE, and Ensemble Design"; no residual references to differential equations or unvalidated theoretical modeling appear anywhere in the text, figures, or tables.)

This revision strengthens the manuscript’s coherence, rigor, and alignment with the experimental validation of the hybrid ensemble and lightweight audit ledger. We appreciate the reviewer’s feedback, which has helped improve the overall quality and focus of the work.

- **Manuscript Clarity and Structure: The paper is lengthy, contains repetition, and includes several grammatical and syntactic issues. Some sections mix unrelated topics (e.g., blockchain details within ML model descriptions). A thorough English-language and structural revision is needed for clarity.**

**Response:** **Response**

We sincerely thank the reviewer for these valuable observations and fully agree that improvements in clarity, conciseness, and structural organization are essential for readability and impact.In the revised manuscript, we have undertaken a comprehensive editing and reorganization process to address these concerns systematically:

- **Reduction of length and elimination of repetition**:
  - Redundant statements (e.g., multiple near-identical listings of research gaps, objectives, and model enumerations in the Introduction and Proposed Work sections) have been consolidated or removed.
  - Overlapping descriptions of contributions across Abstract, Introduction, and Conclusions have been streamlined to appear only once in the most appropriate location.
  - Overall manuscript length has been reduced by approximately 15–20% without loss of essential content.
- **Resolution of grammatical and syntactic issues**:
  - All sections have been carefully proofread and edited for grammar, syntax, consistency, and style.
  - Examples of corrections: uniform dataset notation ("CIC-IDS2017" throughout), elimination of run-on sentences (e.g., rephrased repetitive "This study proposed..." constructions), corrected subject–verb agreement, and improved sentence flow.
- **Separation of unrelated topics and improved structural flow**:
  - Blockchain-related content has been fully consolidated into a dedicated subsection ("Blockchain-Inspired Immutable Audit Logging"), removing scattered references from machine learning model descriptions.
  - Methods section has been reorganized into clearly delineated subsections with logical progression: Leakage-Proof Preprocessing Pipeline → Ensemble Model Integration → Blockchain-Inspired Immutable Audit Logging → Justification of Design Choices → Statistical Testing Approach.
  - Smooth transitions and cross-references have been added between sections to guide the reader.
- **Enhanced reproducibility and supplementary guidance**:
  - The public GitHub repository includes an updated README.md with clear step-by-step instructions and a structured main_notebook.ipynb that mirrors the revised manuscript’s logical flow, further supporting clarity for readers wishing to reproduce the work.

These revisions have significantly improved the manuscript’s readability, logical coherence, and professional presentation while preserving all scientific content. We are confident that the revised version now offers a clear, concise, and well-structured narrative that meets high publication standards.

- **Novelty Claims Are Overstated:
  Several papers have already combined hybrid ML approaches with blockchain-based IDS designs. Please refine the novelty claim to highlight the specific technical contribution of your work without overstating uniqueness.**

**Response:** We thank the reviewer for this important feedback and agree that novelty claims must be precisely positioned relative to existing work.

In the revised manuscript, we have carefully refined all statements of contribution to avoid overstatement and to clearly acknowledge prior art while highlighting the specific, incremental advancements of this study:

- Explicit acknowledgment of related works: The Literature Review and Introduction now prominently cite and discuss prior blockchain-integrated IDS efforts (e.g., Mishra 2023 [10], Babu et al. 2022 [11], Almalki et al. 2024 [22], Sorour et al. 2025 [24]) and hybrid/ensemble approaches (e.g., Suresh et al. 2024 [16], Urmi et al. 2024 [20], Asgharzadeh et al. 2024 [17]).
- Refined novelty statement: Claims of “first” or absolute uniqueness have been removed. Instead, the specific technical contributions are articulated as:
  - A full-spectrum 7-model hybrid ensemble that integrates both supervised (SVM, Random Forest, CatBoost, DNN) and unsupervised (Isolation Forest, One-Class SVM, Autoencoder) paradigms with adaptive weighted voting derived from 5-fold cross-validation F1-scores.
  - A strictly leakage-proof preprocessing pipeline combining PCA (95% variance) and SMOTE (1:1 ratio) applied in the correct order after train–test split.
  - A lightweight, single-node blockchain-inspired immutable ledger providing quantified minimal overhead (average 0.09 s/transaction, negligible storage growth) and demonstrated tamper-evidence for forensic auditing.
  - Comprehensive evaluation including pre/post-integration statistical analysis, ablation studies, and cross-dataset validation.

The updated text in the Introduction -

While prior studies have effectively integrated machine learning ensembles with blockchain for enhanced intrusion detection, particularly in distributed, IoT, or vehicular environments, the proposed H-IDS advances the field through several targeted integrations: (1) a full-spectrum hybrid ensemble of seven diverse base learners with adaptive weighted voting derived from 5-fold cross-validation F1-scores; (2) a strictly leakage-proof preprocessing pipeline; (3) a lightweight private blockchain-inspired auditing layer with quantified minimal overhead.

The Literature Review has also been expanded with additional recent 2024–2025 citations (e.g., [5,17,18,24,33–38]) to provide a more balanced and up-to-date comparison (Table 1 and Table 12 updated accordingly).

(Revised Manuscript: Introduction – "Research Gaps and Contributions" subsection, lines ~100–200; Literature Review section, lines ~150–350; Table 12 "State-of-the-Art Comparison", lines ~1100–1150; Conclusions, lines ~1300–1350.)

These refinements ensure that the novelty is presented accurately and modestly as a targeted integration and empirical validation of complementary techniques, building directly on prior research while addressing specific remaining gaps in ensemble diversity, preprocessing rigor, and lightweight auditing. We believe this strengthens the manuscript’s credibility and scientific positioning.

**Minor Comments:**

**Many figures lack clear titles, legends, and axis explanations.**

**Response:** We thank the reviewer for pointing out this important aspect of visual clarity.

In the revised manuscript, all figures have been thoroughly updated to include clear, bold titles, informative legends, properly labeled axes (with units where applicable), and additional explanatory elements as needed. These improvements ensure that each figure is self-explanatory and fully compliant with journal guidelines for accessibility and reproducibility.

Specific enhancements include:

- **Fig. 1**: Added title "Overall Architecture of the Proposed Hybrid Intrusion Detection System (H-IDS)" with a detailed legend explaining components (preprocessing, ensemble models, audit ledger).
- **Fig. 2**: Updated to "Flowchart of the H-IDS Implementation Pipeline" with step-by-step labels and clear indication of the leakage-proof sequence.
- **Fig. 3 and Fig. 4**: Confusion matrices now include bold titles ("Confusion Matrix on NSL-KDD Dataset" and "Confusion Matrix on CIC-IDS2017 Dataset", respectively), axis labels (Predicted vs. Actual classes), and color scaling legend.
- **Fig. 5**: Title updated to "Performance and Overhead of the Blockchain-Inspired Audit Ledger", with dual y-axes clearly labeled (latency in seconds, throughput in transactions/second) and legend for different metrics.
- **Fig. 6**: Title "Distribution of Logged Events by Type, Dataset, and Model" with pie/subplot legends and percentage annotations.
- **Fig. 7**: Title "Attack Detection Timeline and Model Confidence Scores" with time axis labeled and bubble size legend for confidence.
- **Fig. 8**: Title "Pre- and Post-Integration Performance Across Models" with bar grouping legend and error bars indicating standard deviation.
- **Fig. 9**: Heatmap titled "Statistical Significance of Performance Changes Post-Blockchain Integration" with colorbar labeled (Δ metric values) and clear row/column annotations.

All figures now feature high-contrast colors suitable for both color and grayscale printing, consistent font sizes, and concise yet descriptive captions that reference the figure content without redundancy.

(Revised Manuscript: All figures and corresponding captions updated throughout the Results and Analysis section.)

These revisions significantly improve the interpretability and professional presentation of the visual results. We appreciate the reviewer’s attention to detail, which has helped enhance the overall quality of the manuscript.

**Several tables would benefit from more concise formatting.**

**Response:** We thank the reviewer for this useful suggestion to further improve the presentation of our results.

In the revised manuscript, all tables have been carefully reformatted for greater conciseness, clarity, and readability while retaining all essential information:

- **General improvements**:
  - Redundant or overlapping rows/columns have been merged or removed.
  - Table layouts have been simplified (e.g., fewer decimal places where appropriate, consistent significant figures).
  - Unnecessary coloring and shading have been eliminated to ensure compatibility with both color and grayscale printing.
  - Clear, descriptive captions have been added or enhanced for each table, making them more self-explanatory.
  - Units and abbreviations are now consistently defined in captions or footnotes.
- **Specific changes**:
  - **Table 2**: Performance summary consolidated by averaging across folds; redundant confusion matrix counts removed from the main table (retained only in figures).
  - **Table 3**: Tamper-resistance metrics streamlined into fewer rows with combined overhead indicators.
  - **Table 4**: Logged event distribution simplified by merging low-frequency categories and presenting percentages rounded to whole numbers.
  - **Table 5**: Attack detection events reduced to essential columns (model, confidence, timestamp summary).
  - **Table 6–10**: Metrics rounded to three decimal places where possible; redundant sub-tables merged.
  - **Table 11** (Ablation study): Rows reordered for logical progression and non-significant differences grayed out only via footnote (no coloring).
  - **Table 12** (SOTA comparison): Columns reduced by focusing on key differentiating features; prior works grouped thematically.

These reforms make the tables more compact and easier to interpret at a glance without sacrificing scientific content.

(Revised Manuscript: All tables and corresponding captions updated throughout the Results and Analysis section)

We believe these changes significantly enhance the overall professionalism and readability of the tabular data presentation. Thank you for helping us refine this aspect of the manuscript.

- **The implementation section mixes low-level details (e.g., Docker commands) with high-level descriptions; consider moving technical commands to supplementary material.**

**Response:** We thank the reviewer for this observation regarding the clarity of the implementation description.

Upon review, we confirm that the original manuscript did not include any Docker commands or similar low-level deployment instructions in the main text. The "Experimental Setup and Implementation" section focused primarily on high-level aspects: Python version (3.9), key libraries (scikit-learn v1.5.3, CatBoost v1.2, TensorFlow/Keras, pandas, numpy, hashlib), hardware specifications (Intel Xeon 2.4 GHz, 64 GB RAM, NVIDIA RTX 3080 GPU), and platform (Google Colab with Python 3.9).

To further improve focus and avoid any potential mixing of abstraction levels:

- All references to specific execution environments (e.g., Google Colab) and minor library versions have been streamlined in the main text to a concise high-level summary.
- Detailed deployment or environment setup instructions (should any be added in future extensions) would be placed in the repository README.
- The public GitHub repository now includes a comprehensive README.md with clear sections on environment setup, dependencies (requirements.txt), and step-by-step execution instructions using the provided scripts and main_notebook.ipynb. This serves as the primary supplementary material for technical commands and full reproducibility.

Full reproducibility is supported via the public GitHub repository, which includes a requirements.txt file listing all dependencies (installable via pip install -r requirements.txt in a virtual environment) and a main_notebook.ipynb for end-to-end execution.

(Revised Manuscript: "Experimental Setup and Implementation" subsection streamlined to high-level overview, lines -440-516)

This ensures the main text remains focused on scientific methodology and results, while complete technical details for replication are readily available in the public codebase and its documentation. We appreciate the reviewer’s attention to this aspect and believe the current structure appropriately balances readability with reproducibility.
