# Supplementary material for: A lightweight blockchain-inspired hybrid intrusion detection system with ensemble learning for tamper-proof auditing
Source: PLoS One. 2026 Sep 11;21(9):e0356878. doi: 10.1371/journal.pone.0356878 (PMC13567761; doi:10.1371/journal.pone.0356878)
Supplement: Supplementary file 4 [file pone.0356878.s004.docx]

**Response to Reviewers**

**Manuscript ID:** PONE-D-25-59640R2
**Title:** *A Lightweight Blockchain-Inspired Hybrid Intrusion Detection System with Ensemble Learning for Tamper-Proof Auditing*

Dear Academic Editor and Reviewers,

We sincerely thank both reviewers for their constructive and insightful evaluations. Reviewer #1’s positive assessment of the manuscript’s technical rigor, reproducibility, and balanced positioning of contributions is greatly appreciated. Reviewer #2’s recognition of the substantial improvements from the previous round, particularly the clarified leakage-safe preprocessing pipeline, moderated novelty claims, and explicit single-node scope of the audit mechanism, has further strengthened the manuscript.

All remaining points have been fully addressed through targeted clarifications, terminology standardization, and cautious statistical interpretation. The revised manuscript (tracked-changes version provided) incorporates all requested refinements while preserving the original scientific contributions.

Below we provide a complete point-by-point response with exact manuscript insertions.

**Reviewer #1:** This manuscript presents a hybrid intrusion detection system (H-IDS) integrating supervised and unsupervised machine learning models within a weighted ensemble framework, combined with a lightweight blockchain-inspired immutable audit mechanism. The work addresses key challenges in IDS research, including class imbalance, dimensionality reduction, ensemble robustness, and tamper-evident logging.

The manuscript is technically sound, methodologically detailed, and reproducible (code and datasets are publicly available). The authors clearly position their contribution as a refinement and integration of existing approaches rather than claiming entirely novel components, which strengthens the scientific integrity of the paper.

**Response:**
We sincerely thank the reviewer for this encouraging assessment.

**Reviewer #2**: I appreciate the substantial revisions made in response to the previous review round. The manuscript has improved significantly in clarity, methodological transparency, and positioning of its contributions. In particular, the clarification of the leakage-proof preprocessing pipeline (train–test split prior to scaling, PCA, and SMOTE), the softened novelty claims, and the explicit limitation of the blockchain-inspired component to a single-node hash-chained audit mechanism are positive and constructive improvements.

The integration of supervised and unsupervised models within a weighted ensemble framework is clearly described, and the experimental setup is reproducible, with code and datasets made publicly available. The manuscript is generally well structured and intelligible.

However, a few issues still require clarification before final acceptance:

**1- Statistical Rigor**
The paired t-test analysis appears to be conducted with a very small number of repetitions (n = 3, df = 2). While the computations are technically valid, such a small sample size limits statistical power and the robustness of inferential claims. The authors are encouraged either to:
1-Provide clarification on what constitutes the three paired observations and justify the adequacy of this sample size, or

**Response:**

We thank the reviewer for the constructive evaluation and clear guidance for final refinement. Each remaining issue has been addressed as detailed below.
We fully agree that n = 3 constrains inferential robustness. The paired observations represent three independent full-pipeline experimental executions comparing pre-ledger and post-ledger performance under identical configurations. Each execution includes preprocessing, model training, ensemble inference, and ledger integration, conducted with distinct random seeds to ensure independence and reproducibility.

To address the reviewer’s concern, we have:

- clarified the origin of paired observations
- explicitly stated statistical limitations
- tempered claims from “statistically significant” to cautious interpretation
- reported effects as indicative trends

**Manuscript Insert LINE NO (971-977) - (Statistical Analysis)**

The paired t-test comparing pre- and post-audit performance derives from three independent executions of the complete training–evaluation pipeline (preprocessing, model training, ensemble inference, and ledger integration) conducted with different random seeds. These executions constitute the paired observations (n = 3, df = 2). Although the paired t-test is mathematically valid for this sample size, the limited number of repetitions reduces statistical power; therefore, results are interpreted as indicative of consistent performance trends rather than definitive population-level inference. Absolute metric differences and effect magnitudes are reported alongside p-values.

**Manuscript Insert, LINE NO (938-941) - (Ledger Impact)**

The audit mechanism produced consistent reductions in false positives across all evaluated configurations. While paired comparisons indicate an improvement trend (paired t-test, n = 3, p < 0.05), the small number of observations limits statistical power; thus, results are interpreted as indicative rather than conclusive evidence of improvement.

**Manuscript Insert, LINE NO (25-26) - Abstract**

Original: “statistically significant reductions in false positives.”
Revised: observed reductions in false positives (paired t-test, n = 3, p < 0.05,).

2-Temper claims of statistical significance accordingly.

**Response:**
We agree with the reviewer that, given the limited number of paired observations (n = 3), strong inferential claims are not warranted. Accordingly, all statements implying definitive statistical significance have been revised to reflect cautious interpretation. Throughout the manuscript, wording such as “statistically significant improvement” has been replaced with neutral phrasing (e.g., “observed reduction,” “improvement trend,” or “paired t-test, n = 3”). We also explicitly state the limited statistical power and interpret the results as indicative rather than confirmatory.

**Revisions made:**

- **Abstract:**
  “statistically significant reductions” →
  “observed reductions (paired t-test, n = 3,p < 0.05)” (Line 25-26)
- **Research Methods – Statistical Analysis (line 494-496):**

As the paired t-test is mathematically correct for n = 3, the small number of repetitions means that the results are viewed as indicative of trends rather than making any statistical inference on the population.

**Results – Ledger Impact (**Line 938-941)

The audit mechanism has consistently reduced false positives in all configurations under evaluation. Although the paired comparison shows a trend of improvement (paired t-test, n = 3, p < 0.05), the low number of samples makes the results more indicative than conclusive of improvement.

These revisions appropriately temper statistical claims while preserving the descriptive reporting of observed performance differences.

**3-Near-Perfect Supervised-Only Performance**
The reported 99.9%+ accuracy on benchmark datasets (NSL-KDD and CIC-IDS2017) is exceptionally high. Although the preprocessing pipeline is described as leakage-resilient, the authors should further clarify how cross-validation, validation splits, and ensemble weighting are strictly isolated from the final test set to eliminate any possibility of inadvertent information leakage.

**Response:**
We appreciate this important safeguard check. The pipeline already enforced strict isolation; however, this was not sufficiently explicit. The manuscript now unequivocally states that:

- train/test split occurs first
- PCA fitted only on training data
- SMOTE applied only on training data
- cross-validation confined to training partition
- ensemble weights derived solely from training CV
- test set used exactly once for final evaluation

These clarifications have been added to Methods, Ensemble Integration, and Experimental Setup.

**Manuscript Insert, LINE NO (301-309), Research Methods (Leakage-Safe Pipeline)**

To eliminate any possibility of information leakage, all preprocessing, model selection, and weighting steps were confined strictly to the training data. The workflow proceeds as follows: (1) the dataset is first partitioned into fixed training and test sets; (2) PCA is fitted exclusively on the training set and then applied to the test set using the learned transformation; (3) SMOTE is applied only to the training data after PCA; (4) cross-validation, hyperparameter tuning, and ensemble weight estimation are performed solely within the training partition; (5) the held-out test set is accessed once for final performance evaluation. No stage of model fitting, weighting, or threshold selection uses information from the test data.

**Manuscript Insert, LINE NO (395-397), Ensemble Integration**

Ensemble weights were derived solely from macro-F1 scores obtained during cross-validation on the training data; no test-set information influenced weight assignment or decision thresholds.

**Manuscript Insert, LINE NO (608-610), Experimental Setup**

The final ensemble model was tested only once on the untouched test set after the completion of all the training, validation, and calibration procedures.

**Manuscript Insert, LINE NO (1028-1032), Discussion (Performance Interpretation)**

The near-perfect accuracy observed in the supervised-only ensemble should be interpreted in the context of leakage-resilient preprocessing and fixed benchmark datasets. Because dimensionality reduction, balancing, and weight estimation were confined entirely to the training partition, the reported performance does not arise from inadvertent test-set information leakage.

**4-Blockchain Terminology Precision**
The manuscript correctly clarifies that the proposed mechanism is single-node and does not implement decentralized consensus. To avoid possible conceptual confusion, the authors may consider ensuring consistent terminology (e.g., “blockchain-inspired hash-chained audit log”) throughout the manuscript to accurately reflect the security guarantees provided.

**Response:**
We fully agree and have standardized terminology throughout the manuscript to:

**“blockchain-inspired hash-chained audit log (single-node)”**

All ambiguous terms (“blockchain ledger,” “private blockchain,” “blockchain-based”) have been replaced to avoid implying decentralization or consensus.

**Global Terminology Revision**

- “blockchain ledger” → blockchain-inspired hash-chained audit log
- “private blockchain” → single-node hash-chained audit log
- “blockchain-based” → blockchain-inspired

**Manuscript Insert, LINE NO (443-446), Lightweight Blockchain-Inspired Audit Ledger**

The proposed mechanism is a single-node blockchain-inspired hash-chained audit log that links detection records using SHA-256 hashes without decentralized consensus, mining, peer-to-peer networking, or smart contracts. Its purpose is tamper-evident sequencing of intrusion-detection events rather than distributed trust establishment.

**Manuscript Insert, LINE NO (19-22), Abstract**

A lightweight blockchain-inspired hash-chained audit log provides tamper-evident logging of detection events in a single-node deployment without decentralized consensus.

The revised manuscript now incorporates:

- explicit clarification and cautious interpretation of paired t-test results (n = 3)
- unambiguous statements confirming zero test-set leakage across all pipeline stages
- uniform terminology: “blockchain-inspired hash-chained audit log”
- strengthened methodological transparency and reproducibility

We sincerely thank the reviewers and Academic Editor for their valuable guidance, which has substantially improved the clarity, rigor, and precision of this work. We believe the manuscript is now fully suitable for publication in *PLOS ONE* and remain available for any further clarification if required.

Sincerely,
**Shailendra Mishra** (on behalf of all authors)
Corresponding author: **Reem Alshenaifi**
Email: r.alshnaifi@mu.edu.sa
