# Supplementary material for: A lightweight blockchain-inspired hybrid intrusion detection system with ensemble learning for tamper-proof auditing
Source: PLoS One. 2026 Sep 11;21(9):e0356878. doi: 10.1371/journal.pone.0356878 (PMC13567761; doi:10.1371/journal.pone.0356878)
Supplement: Supplementary file 5 [file pone.0356878.s005.docx]

**Response to Reviewers**

PONE-D-25-59640R3

A Lightweight Blockchain-Inspired Hybrid Intrusion Detection System with Ensemble Learning for Tamper-Proof Auditing

PLOS One

Journal Requirements:

1. If the reviewer comments include a recommendation to cite specific previously published works, please review and evaluate these publications to determine whether they are relevant and should be cited. There is no requirement to cite these works unless the editor has indicated otherwise.

**Response-**

We thank the reviewers for their valuable suggestions, including recommendations to consider citing additional previously published works. We have carefully reviewed the suggested references and evaluated their relevance to our study. Relevant works have been incorporated where they meaningfully support the context and discussion of the manuscript, while those not directly aligned with the scope of this study were not included, in accordance with the journal guidelines. We also appreciate the positive feedback from both reviewers. We are grateful for Reviewer #1’s acknowledgment of the improved structure and academic clarity, and Reviewer #2’s detailed evaluation highlighting enhancements in methodological transparency, data handling, and reproducibility.

We have addressed all comments accordingly and believe the manuscript has been significantly strengthened. We thank the reviewers and the editor for their constructive feedback and consideration.

2. Please review your reference list to ensure that it is complete and correct. If you have cited papers that have been retracted, please include the rationale for doing so in the manuscript text, or remove these references and replace them with relevant current references. Any changes to the reference list should be mentioned in the rebuttal letter that accompanies your revised manuscript. If you need to cite a retracted article, indicate the article’s retracted status in the References list and also include a citation and full reference for the retraction notice.

**Response-** We have carefully reviewed the entire reference list(1–40) to ensure completeness, accuracy, and relevance in accordance with the journal guidelines. All cited works were verified for their publication status. To the best of our knowledge, none of the references included in the manuscript have been retracted.

Additionally, we ensured that all references are current, relevant to the scope of the study, and appropriately cited within the manuscript. No retracted articles were identified; therefore, no replacements or justifications were required.

We confirm that the reference list complies with the journal’s requirements.

[Note: HTML markup is below. Please do not edit.]

Reviewer's Responses to Questions

**Comments to the Author**

1. If the authors have adequately addressed your comments raised in a previous round of review and you feel that this manuscript is now acceptable for publication, you may indicate that here to bypass the “Comments to the Author” section, enter your conflict of interest statement in the “Confidential to Editor” section, and submit your "Accept" recommendation.

Reviewer #1: All comments have been addressed

Reviewer #2: (No Response)

**Response-** We thank the reviewers for their time and valuable feedback. We are pleased that Reviewer #1 has confirmed that all previously raised comments have been adequately addressed. We also appreciate Reviewer #2’s earlier constructive suggestions, which helped us improve the clarity, methodology, and overall quality of the manuscript.

We believe that all concerns have been resolved and that the manuscript is now suitable for publication.

2. Is the manuscript technically sound, and do the data support the conclusions?

The manuscript must describe a technically sound piece of scientific research with data that supports the conclusions. Experiments must have been conducted rigorously, with appropriate controls, replication, and sample sizes. The conclusions must be drawn appropriately based on the data presented.

Reviewer #1: Yes

Reviewer #2: Partly

**Response-**

3. Has the statistical analysis been performed appropriately and rigorously?

Reviewer #1: Yes

Reviewer #2: No

**Response:**

We thank the reviewers for their careful evaluation of the statistical analysis. We are encouraged by Reviewer #1’s positive assessment and have further strengthened the manuscript to address Reviewer #2’s concern regarding statistical rigor.

In the revised manuscript, we have clarified and improved the statistical methodology as follows:

- We explicitly described the paired t-test setup, including the use of independent experimental runs (n = 3) with different random seeds, ensuring reproducibility and proper pairing of observations.
- We verified and reported key assumptions of the paired t-test, including normality , independence of observations, and variance consistency.
- The p-values with effect size (Cohen’s d) and confidence interval estimates to provide a more comprehensive statistical interpretation.
- We clearly acknowledged the limitation of the small sample size (n = 3) and explicitly stated that the statistical results should be interpreted as indicative trends rather than definitive population-level conclusions.

These revisions improve the transparency, correctness, and interpretation of the statistical analysis. While the paired t-test is mathematically valid for the given experimental design, we have carefully moderated our claims and aligned conclusions with the available evidence.

We believe the statistical analysis is now appropriately conducted, clearly explained, and consistent with the scope of the study.

4. Have the authors made all data underlying the findings in their manuscript fully available?

The [PLOS Data policy](https://eur03.safelinks.protection.outlook.com/?url=http%3A%2F%2Ftrack.editorialmanager.com%2FCL0%2Fhttp%3A%252F%252Fwww.plosone.org%252Fstatic%252Fpolicies.action%2523sharing%2F1%2F010f019cf9f02162-e4cd85f8-20f3-43fe-ad7e-4d55c0c16931-000000%2FRDsYRzR7u377v0G7QJ0Wgv9MT6vnBCQEgU0LbpwYY-s%3D254&data=05%7C02%7Cs.mishra%40mu.edu.sa%7Cbc8fd2fddb6348c655e008de83d92d28%7Cadd7e95a90f746f58bb7c861a9d333a5%7C0%7C0%7C639093165992739311%7CUnknown%7CTWFpbGZsb3d8eyJFbXB0eU1hcGkiOnRydWUsIlYiOiIwLjAuMDAwMCIsIlAiOiJXaW4zMiIsIkFOIjoiTWFpbCIsIldUIjoyfQ%3D%3D%7C0%7C%7C%7C&sdata=lc1rrnxDbCCBaA0NqFBe8nfw2Dx2mHC5F11rySpqDck%3D&reserved=0) requires authors to make all data underlying the findings described in their manuscript fully available without restriction, with rare exception (please refer to the Data Availability Statement in the manuscript PDF file). The data should be provided as part of the manuscript or its supporting information, or deposited to a public repository. For example, in addition to summary statistics, the data points behind means, medians and variance measures should be available. If there are restrictions on publicly sharing data—e.g. participant privacy or use of data from a third party—those must be specified.

Reviewer #1: Yes

Reviewer #2: Yes

**Response:**

We thank the reviewers for confirming that the data underlying the findings are fully available. All datasets used in this study (NSL-KDD and CIC-IDS2017) are publicly accessible and have been properly referenced in the manuscript.

In addition, we have ensured that all relevant data, preprocessing steps, and implementation details are clearly described, and the source code has been made publicly available to support reproducibility.

We confirm that the manuscript complies with the PLOS Data Availability policy.

5. Is the manuscript presented in an intelligible fashion and written in standard English?

PLOS ONE does not copyedit accepted manuscripts, so the language in submitted articles must be clear, correct, and unambiguous. Any typographical or grammatical errors should be corrected at revision, so please note any specific errors here.

Reviewer #1: Yes

Reviewer #2: Yes

**Response:**

We thank the reviewers for their positive assessment of the manuscript’s clarity and language. We have carefully proofread the revised manuscript to ensure that it is written in clear, correct, and standard English, and that all typographical and grammatical issues have been addressed.

We confirm that the manuscript is presented in an intelligible and unambiguous manner in accordance with the journal’s requirements.

6. Review Comments to the Author

Please use the space provided to explain your answers to the questions above. You may also include additional comments for the author, including concerns about dual publication, research ethics, or publication ethics. (Please upload your review as an attachment if it exceeds 20,000 characters)

Reviewer #1: well writen compared with last time considering the rigorous template and the bullet point to prove the academic thesis

**Response:**

We sincerely thank the reviewer for the positive feedback and for recognizing the improvements in the manuscript. We appreciate the acknowledgment of the enhanced structure, adherence to the rigorous template, and the clearer presentation of the academic contributions.

We are grateful for the reviewer’s guidance throughout the revision process, which has helped us significantly improve the overall quality and clarity of the manuscript.

Reviewer #2: Thank you for the careful revision of the manuscript and the detailed responses to the reviewers’ comments. The revised version has improved in clarity and methodological transparency. In particular, the clarification of the leakage-safe preprocessing pipeline, the moderation of statistical claims, and the consistent terminology describing the blockchain-inspired hash-chained audit log have strengthened the manuscript.

The methodology is clearly described, and the availability of the source code and datasets improves the reproducibility of the study. The explanation of the training–test separation and ensemble weighting procedure also addresses the earlier concerns regarding potential data leakage.

While the statistical comparison is based on a limited number of repetitions, the authors appropriately acknowledge this limitation and present the results cautiously. Overall, the manuscript now provides a technically sound and clearly presented study.

I believe the manuscript is suitable for publication in PLOS ONE.

**Response :**

We sincerely thank the reviewer for the thorough evaluation and for recognizing the improvements made in the revised manuscript. We are grateful for the positive feedback regarding the enhanced clarity, methodological transparency, and overall presentation of the study.

We appreciate the acknowledgment of the leakage-safe preprocessing pipeline, the careful moderation of statistical claims, and the consistent description of the blockchain-inspired hash-chained audit log. We are also pleased that the explanations of training–test separation and ensemble weighting have addressed the earlier concerns regarding potential data leakage.

We acknowledge the reviewer’s note regarding the limited number of repetitions and confirm that this limitation has been clearly stated and carefully considered in the interpretation of results.

We are thankful for the reviewer’s support and for considering the manuscript suitable for publication in PLOS ONE.

We sincerely thank the reviewers and the editor for their time, constructive feedback, and valuable suggestions, which have significantly improved the quality and clarity of our manuscript. We greatly appreciate their consideration and support, and we look forward to the final decision.

Shailendra Mishra

(on the behalf of All authors)
