# Supplementary material for: A lightweight blockchain-inspired hybrid intrusion detection system with ensemble learning for tamper-proof auditing
Source: PLoS One. 2026 Sep 11;21(9):e0356878. doi: 10.1371/journal.pone.0356878 (PMC13567761; doi:10.1371/journal.pone.0356878)
Supplement: Supplementary file 6 [file pone.0356878.s006.docx]

**Point to Point -RESPONSE TO ACADEMIC EDITOR & REVIEWER# 2 COMMENTS**

**Additional Editor Comments:**

The manuscript is technically sound, methodologically rigorous, and presents a valuable H-IDS framework combining high detection accuracy with blockchain-inspired auditability. The study is suitable for publication after minor revisions, mainly requiring brief clarification on scalability under high-volume attacks, handling of encrypted traffic, and provision of code/data availability details to support reproducibility. No major methodological or ethical concerns were identified.

**Response:**

We sincerely thank the Academic Editor for the positive evaluation of our work and for identifying three important areas where additional clarification would strengthen the manuscript.

To address these recommendations:

1. **Scalability under high-volume attacks** -Scalability under high volume attack situations has been improved by having a separate discussion on the scalability under volumetric DDoS attacks, the throughput of the blockchain-based audit ledger, the behavior under volumetric DDoS attacks, and potential future scalability improvements such as asynchronous event buffering, batching of events, distributed ledger partitioning, and stream processing. The discussion now clearly indicates that the detection and audit modules are independent, enabling scalability without impacting the detection module.
2. **Handling of encrypted traffic**-Compatibility of the H-IDS with encrypted traffic was addressed through the explicit specification that the H-IDS works solely with statistical and metadata-based features derived from packet headers and flows, without relying on the content of the payload at the application layer. Therefore, the approach can be used effectively with encrypted flows utilizing TLS/SSL encryption. It is also noted that for application layer attacks performed using encrypted flows, other measures might need to be implemented.
3. **Provision of code/data availability details to support reproducibility-** The reproducibility of the study and compliance with open science principles have been enhanced through the explicit inclusion of the public GitHub repository (<https://github.com/rubaamff/Hybrid-IDS-Blockchain>) and the DOI-registered Zenodo archive (<https://doi.org/10.5281/zenodo.18009378>), ensuring full reproducibility, transparency, and long-term accessibility in accordance with PLOS ONE open science policy. The release includes the complete source code, data pre-processing pipeline, ensemble weighting algorithm, blockchain-based audit logging components, experimental configurations, and all other artifacts necessary to reproduce the reported results.

Also, all the descriptions of manuscripts have been checked to ensure that they comply with the audit technique that is used in the system, which is a minimalistic hash chaining audit ledger inspired by blockchains and implemented using Python code.The manuscript has been revised accordingly.

**RESPONSE TO REVIEWER #2**

**Comments:**

Reviewer #2: The manuscript presents a technically sound and well-executed study on a Hybrid Intrusion Detection System (H-IDS) that integrates machine learning ensembles with a blockchain-inspired auditing layer. The core strength of the work lies in its dual focus: achieving high detection accuracy (98%+) while providing a verifiable, tamper-proof forensic trail for network events. The methodology demonstrates high academic rigor, particularly in its handling of data preprocessing to prevent leakage.

1. Technical Rigor and Methodology The authors have established a robust pipeline for evaluating their H-IDS: Data Integrity: The use of Min-Max Scaling, PCA, and SMOTE is handled correctly by applying these transformations only after the train-test split. This ensures that the results are not artificially inflated by data leakage. Ensemble Design: The combination of seven diverse models—ranging from supervised learners like CatBoost and DNN to unsupervised anomaly detectors like Autoencoders—is well-justified for capturing both known and zero-day threats. Auditability: The integration of a SHA-256 hash-chaining mechanism for logging is a significant contribution. The "Tamper Evidence" analysis effectively proves the system's ability to identify unauthorized modifications to historical logs.

2. Statistical AnalysisThe statistical validation is appropriate for a study of this nature: The use of 5-fold cross-validation ensures that the performance metrics are stable and generalizable. The application of a paired t-test ($p < 0.05$) provides the necessary formal evidence that the hybrid ensemble's performance gains are statistically significant compared to individual base learners.

**3. Areas for Improvement & Recommendations** -While the manuscript is strong, addressing the following minor points would enhance its impact: **Scalability Concerns**: The throughput is reported at 210 blocks per second. The authors should include a brief discussion on how the system would behave under extreme traffic conditions, such as a volumetric DDoS attack, where log generation might exceed this rate. **Encrypted Traffic**: Given that the majority of modern network traffic is encrypted, it would be beneficial for the authors to clarify if the H-IDS operates on packet headers or if it requires decrypted payloads. **Reproducibility:** To align with PLOS ONE's commitment to open science, the authors are encouraged to provide a link to a public repository containing the custom weighting logic and the ledger implementation.

4. Ethics and Dual Publication Research Ethics: No ethical concerns were identified. The study utilizes public benchmarks (NSL-KDD and CIC-IDS2017) and does not involve human subjects or sensitive personal data. Publication Ethics: There are no indicators of dual publication or self-plagiarism. The work appears original and provides a distinct contribution to the intersection of cybersecurity and distributed ledger principles. Final Recommendation: Weak Accept The manuscript is a strong candidate for publication following minor clarifications regarding scalability and data availability

**Comment 3(1): Scalability Under Extreme Traffic Conditions**

Reviewer Comment:

"The throughput is reported at 210 blocks per second. The authors should include a brief discussion on how the system would behave under extreme traffic conditions, such as a volumetric DDoS attack, where log generation might exceed this rate."

Response:

We would like to acknowledge this insightful comment by the reviewer. We agree that practical deployment considerations under high-volume attack conditions warrant explicit discussion.

The proposed audit ledger supports about 210 blocks/second with average latency of about 5 milliseconds for each block. Overhead is mainly due to SHA-256 hash calculations, metadata serialization, and operations related to append-only ledger management; not due to consensus, mining, peer-to-peer communication, or smart-contract execution, as the design of the suggested system is deliberately aimed at being lightweight, blockchain-inspired single-node hash chain audit ledger. For volumetric DDoS attacks, the rate of alerts generated can briefly surpass the logging capabilities of the ledger. Such instances may impose logging backpressure; however, the proposed architecture distinguishes between detection capability and audit logging capability. This allows for potential improvement regarding scalability in future implementations via asynchronous event buffering, dedicated ledger writers, batching of blocks, ledger partitioning across multiple nodes, and use of stream processing frameworks, such as Apache Kafka or Apache Flink. To address this concern, a new discussion has been incorporated into the manuscript in discussion section also.

**TRACK CHANGE MANUSCRIPT**

Section:

**Lightweight Blockchain-Inspired Audit Ledger** - (Line No 795-812)

**Discussion Section** = (Line No –1009-1021)

**Comment 3 (2): Encrypted Traffic Handling**

Reviewer Comment:

"Given that the majority of modern network traffic is encrypted, it would be beneficial for the authors to clarify if the H-IDS operates on packet headers or if it requires decrypted payloads."

Response:

We thank the reviewer for highlighting this important deployment consideration.

The proposed H-IDS is based on statistical and metadata-based features extracted from the network traffic at the flow level. The NSL-KDD and CICIDS2017 dataset used in this study includes flow-duration, packets, bytes, inter-arrival, TCP flag frequency, connection metadata, and other relevant features. These features are extracted from the headers of the packets and the flow logs and are independent of application-layer payloads. Therefore, the approach proposed in this research work will be intrinsically compatible with any TLS/SSL encryption environment since the encrypted behavior at the transport layer and network layer can still be captured using the proposed approach. In addition, the application-layer attack specially crafted to behave as benign encrypted flows may require additional techniques including TLS fingerprint analysis, behavioral profiling, or sequence-based anomaly detection. The manuscript has been revised accordingly. To address this concern, a new discussion has been incorporated into the manuscript in discussion section also.

**TRACK CHANGE MANUSCRIPT**

Section:

Experimental Setup and Implementation (Line no- 503-512)

Discussion Section = (Line No –1030-1042)

**Comment 3(3): Reproducibility and Open Science Compliance**

Reviewer Comment:

"To align with PLOS, ONE's commitment to open science, the authors are encouraged to provide a link to a public repository containing the custom weighting logic and the ledger implementation."

Response:

We fully agree with the reviewer regarding the importance of reproducibility and open science.

To support independent verification of all reported experiments, all the reported experiments, the entire implementation of the H-IDS architecture has been made freely available for use by all users. This includes leakage-proof pre-processing, PCA and SMOTE processing modules, supervised and unsupervised models training scripts, adaptive ensemble weighting process implementation, SHA-256 hash-chain inspired audit ledger, notebooks, configuration files, and other reproducibility resources.

The repository is publicly accessible at:

Public GitHub repository (<https://github.com/rubaamff/Hybrid-IDS-Blockchain>) and the DOI-registered Zenodo archive (<https://doi.org/10.5281/zenodo.18009378>), ensuring full reproducibility, transparency, and long-term accessibility in accordance with PLOS ONE open science policy.

The manuscript has been revised accordingly.

**TRACK CHANGE MANUSCRIPT**

Section: Leakage-Proof Preprocessing Pipeline

Line No -269-274

Section: Data Availability Statement ( After Conclusion )

Line No -1089-1096

We sincerely thank the Academic Editor and Reviewer #2 for their constructive comments, which have helped improve the clarity, transparency, reproducibility, consistency, and practical relevance of the manuscript. We believe that the revised manuscript fully addresses all remaining concerns and respectfully submit it for further consideration.

Sincerely,

Shailendra Mishra

(On behalf of all the authors)
