# Supplementary material for: A lightweight blockchain-inspired hybrid intrusion detection system with ensemble learning for tamper-proof auditing
Source: PLoS One. 2026 Sep 11;21(9):e0356878. doi: 10.1371/journal.pone.0356878 (PMC13567761; doi:10.1371/journal.pone.0356878)
Supplement: Peer review history

# Peer review: Decision Letter 0

**DOI:** 10.1371/journal.pone.0356878.r001

**Authors:** Muhammad Anwar

15 Dec 2025

Dear Dr. Mishra,

Thank you for submitting your manuscript to PLOS ONE. After careful consideration, we feel that it has merit but does not fully meet PLOS ONE’s publication criteria as it currently stands. Therefore, we invite you to submit a revised version of the manuscript that addresses the points raised during the review process.

Please submit your revised manuscript by Jan 29 2026 11:59PM. If you will need more time than this to complete your revisions, please reply to this message or contact the journal office at plosone@plos.org. When you're ready to submit your revision, log on to https://www.editorialmanager.com/pone/ and select the 'Submissions Needing Revision' folder to locate your manuscript file.

- A letter that responds to each point raised by the academic editor and reviewer(s). You should upload this letter as a separate file labeled 'Response to Reviewers'.
- A marked-up copy of your manuscript that highlights changes made to the original version. You should upload this as a separate file labeled 'Revised Manuscript with Track Changes'.
- An unmarked version of your revised paper without tracked changes. You should upload this as a separate file labeled 'Manuscript'.

If you would like to make changes to your financial disclosure, please include your updated statement in your cover letter. Guidelines for resubmitting your figure files are available below the reviewer comments at the end of this letter.

If applicable, we recommend that you deposit your laboratory protocols in protocols.io to enhance the reproducibility of your results. Protocols.io assigns your protocol its own identifier (DOI) so that it can be cited independently in the future. For instructions see: https://journals.plos.org/plosone/s/submission-guidelines#loc-laboratory-protocols. Additionally, PLOS ONE offers an option for publishing peer-reviewed Lab Protocol articles, which describe protocols hosted on protocols.io. Read more information on sharing protocols at https://plos.org/protocols?utm_medium=editorial-email&utm_source=authorletters&utm_campaign=protocols.

We look forward to receiving your revised manuscript.

Kind regards,

Muhammad Anwar, Ph.D.

Academic Editor

PLOS One

Journal requirements:

When submitting your revision, we need you to address these additional requirements.

1. Please ensure that your manuscript meets PLOS ONE's style requirements, including those for file naming. The PLOS ONE style templates can be found at

https://journals.plos.org/plosone/s/file?id=wjVg/PLOSOne_formatting_sample_main_body.pdf and

https://journals.plos.org/plosone/s/file?id=ba62/PLOSOne_formatting_sample_title_authors_affiliations.pdf

2. Please note that PLOS One has specific guidelines on code sharing for submissions in which author-generated code underpins the findings in the manuscript. In these cases, we expect all author-generated code to be made available without restrictions upon publication of the work. Please review our guidelines at https://journals.plos.org/plosone/s/materials-and-software-sharing#loc-sharing-code and ensure that your code is shared in a way that follows best practice and facilitates reproducibility and reuse.

3. Thank you for stating the following in the Acknowledgments Section of your manuscript:

“The authors extend their appreciation to the Deanship of Postgraduate Studies and Scientific Research at Majmaah University for funding this research work through the project number (R-2025-xxx ).”

We note that you have provided funding information that is not currently declared in your Funding Statement. However, funding information should not appear in the Acknowledgments section or other areas of your manuscript. We will only publish funding information present in the Funding Statement section of the online submission form.

Please remove any funding-related text from the manuscript and let us know how you would like to update your Funding Statement. Currently, your Funding Statement reads as follows:

“The author(s) received no specific funding for this work.”

Please include your amended statements within your cover letter; we will change the online submission form on your behalf.

4. When completing the data availability statement of the submission form, you indicated that you will make your data available on acceptance. We strongly recommend all authors decide on a data sharing plan before acceptance, as the process can be lengthy and hold up publication timelines. Please note that, though access restrictions are acceptable now, your entire data will need to be made freely accessible if your manuscript is accepted for publication. This policy applies to all data except where public deposition would breach compliance with the protocol approved by your research ethics board. If you are unable to adhere to our open data policy, please kindly revise your statement to explain your reasoning and we will seek the editor's input on an exemption. Please be assured that, once you have provided your new statement, the assessment of your exemption will not hold up the peer review process.

5. PLOS requires an ORCID iD for the corresponding author in Editorial Manager on papers submitted after December 6th, 2016. Please ensure that you have an ORCID iD and that it is validated in Editorial Manager. To do this, go to ‘Update my Information’ (in the upper left-hand corner of the main menu), and click on the Fetch/Validate link next to the ORCID field. This will take you to the ORCID site and allow you to create a new iD or authenticate a pre-existing iD in Editorial Manager.

If the reviewer comments include a recommendation to cite specific previously published works, please review and evaluate these publications to determine whether they are relevant and should be cited. There is no requirement to cite these works unless the editor has indicated otherwise.

[Note: HTML markup is below. Please do not edit.]

Reviewers' comments:

Reviewer's Responses to Questions

**Comments to the Author**

1. Is the manuscript technically sound, and do the data support the conclusions?

Reviewer #1: Yes

Reviewer #2: Partly

**********

2. Has the statistical analysis been performed appropriately and rigorously?

Reviewer #1: Yes

Reviewer #2: No

**********

3. Have the authors made all data underlying the findings in their manuscript fully available?

The PLOS Data policy

Reviewer #1: Yes

Reviewer #2: Yes

**********

4. Is the manuscript presented in an intelligible fashion and written in standard English?

Reviewer #1: Yes

Reviewer #2: No

**********

Reviewer #1: The manuscript presents an ambitious hybrid IDS framework integrating supervised and unsupervised models with PCA, SMOTE, and a blockchain-based auditing layer. The overall concept is potentially innovative, especially in its attempt to unify multiple detection paradigms with tamper-proof logging. However, several methodological and interpretive issues need clarification. The supervised-only results showing nearly perfect accuracy (≈99.9%) strongly suggest possible data leakage or overfitting. The blockchain implementation is described inconsistently—referencing Ethereum, Hyperledger Fabric, and a private chain—and the reported performance metrics (e.g., 366 blocks/s, 0.09 s/tx, 0 KB storage overhead) do not align with typical blockchain behavior. Details on hyperparameters, PCA/SMOTE impact, ensemble weighting, statistical testing, and threshold selection are insufficient for reproducibility. Additionally, several figures are difficult to interpret, and the writing contains grammar and structural problems that hinder clarity.

I recommend strengthening the methodological descriptions, adding ablation studies (e.g., PCA-only, SMOTE-only, ensemble vs. individual models), clarifying the blockchain architecture, and validating the supervised model results to rule out leakage. Improving the writing quality and reorganizing sections for smoother flow would also benefit the manuscript. Ethical and data availability statements appear acceptable, though the authors should ensure there is no overlap with their prior work. Overall, the paper has potential but requires significant revision to meet publication standards in terms of rigor, clarity, and reproducibility.

Reviewer #2: Thank you for submitting your manuscript. The topic is important and timely, and the idea of integrating hybrid machine-learning models with blockchain-based logging is potentially valuable. However, several issues require substantial revision before the work can be considered further.

Major Comments

Technical Validity and Overfitting Concerns

The reported results—particularly the near-perfect performance of the supervised models (e.g., 99.9% accuracy on CICIDS2017)—are unrealistic for these datasets and strongly suggest data leakage or methodological errors. Please verify and clearly document the preprocessing pipeline (especially the order of SMOTE, PCA, splitting, and scaling) to ensure no information from the test set influences training.

Blockchain Evaluation Requires Clarification

The blockchain component appears to be a simplified local logging mechanism rather than a true distributed blockchain system. Claims such as 0 KB memory growth, 24,000× faster than Ethereum, and strong tamper-proof guarantees are not supported. Please clarify:

the actual consensus mechanism

whether multiple nodes were used

real ledger growth

how tampering resistance was evaluated

Without this, conclusions about blockchain security are overstated.

Statistical Analysis Not Rigorous

Although t-tests and p-values are mentioned, the methodology is not clearly described or justified. Please provide:

statistical test details

assumptions checks

effect sizes and confidence intervals

rationale for comparing “before” and “after” blockchain results

At present, the statistical conclusions are not reliable.

Mathematical Modeling Section Unrelated to Experiments

The system of differential equations is theoretically interesting but not connected to any experimental results or validated through data. This section should either be removed or linked meaningfully to the system’s behavior.

Manuscript Clarity and Structure

The paper is lengthy, contains repetition, and includes several grammatical and syntactic issues. Some sections mix unrelated topics (e.g., blockchain details within ML model descriptions). A thorough English-language and structural revision is needed for clarity.

Novelty Claims Are Overstated

Several papers have already combined hybrid ML approaches with blockchain-based IDS designs. Please refine the novelty claim to highlight the specific technical contribution of your work without overstating uniqueness.

Minor Comments

Many figures lack clear titles, legends, and axis explanations.

Several tables would benefit from more concise formatting.

The implementation section mixes low-level details (e.g., Docker commands) with high-level descriptions; consider moving technical commands to supplementary material.

Conclusion

The manuscript proposes an interesting direction, but substantial methodological, experimental, and writing improvements are necessary. Addressing the issues above will significantly strengthen the scientific rigor and clarity of the work.

**********

what does this mean?). If published, this will include your full peer review and any attached files.

If you choose “no”, your identity will remain anonymous but your review may still be made public.

**Do you want your identity to be public for this peer review?**  For information about this choice, including consent withdrawal, please see our Privacy Policy

Reviewer #1: **Yes:**  Wenke Du

Reviewer #2: No

**********

[NOTE: If reviewer comments were submitted as an attachment file, they will be attached to this email and accessible via the submission site. Please log into your account, locate the manuscript record, and check for the action link "View Attachments". If this link does not appear, there are no attachment files.]

To ensure your figures meet our technical requirements, please review our figure guidelines: https://journals.plos.org/plosone/s/figures

You may also use PLOS’s free figure tool, NAAS, to help you prepare publication quality figures: https://journals.plos.org/plosone/s/figures#loc-tools-for-figure-preparation.

NAAS will assess whether your figures meet our technical requirements by comparing each figure against our figure specifications.

# Author comment: Author response to Decision Letter 1

**DOI:** 10.1371/journal.pone.0356878.r002

31 Dec 2025

Response to Reviewer --

Response to Reviewers

Manuscript ID: PONE-D-25-59640R1

Title: A Lightweight Blockchain-Inspired Hybrid Intrusion Detection System with Ensemble Learning for Tamper-Proof Auditing (Revised)

Old-A Blockchain-Infused Hybrid IDS with Ensemble-Driven Cyber Defense

Dear Dr. Muhammad Anwar (Academic Editor) and PLOS ONE Editorial Team,

Thank you for considering our manuscript and for the opportunity to revise it based on the reviewers' insightful feedback. We appreciate the constructive comments from Reviewer #1 and Reviewer #2, which have helped us strengthen the methodological rigor, clarity, reproducibility, and overall quality of the work. We have addressed all points raised, incorporating substantial revisions to the manuscript. These include clarifying the preprocessing pipeline to rule out data leakage, softening novelty claims with acknowledgment of prior works, removing the unrelated mathematical modeling section, refining the blockchain description to emphasize its lightweight, audit-oriented nature, adding an ablation study, enhancing statistical reporting, improving figure and table clarity, reorganizing sections for better flow, and conducting a English-language edit to eliminate grammar issues and repetitions.

To facilitate review, we have uploaded:

• A marked-up copy labeled 'Revised Manuscript with Track Changes' showing all modifications.

• An unmarked clean version labeled 'Revised Manuscript'.

We confirm that the revised manuscript adheres to PLOS ONE's style requirements, including file naming, headings (e.g., Level 1 bold 18pt, Level 2 bold 16pt, sentence case), figure citations (e.g., "Fig 1"), and captions (bold titles, placed after first mention). Equations are formatted in Math Type where present (minimal in this work). Tables are presented in editable Word format with concise layouts. The manuscript is double-spaced with continuous line numbers for reference (starting from Title as Line 1).

Addressing Journal Requirements:

1. Style Requirements: The manuscript now fully complies with the provided templates (e.g., https://journals.plos.org/plosone/s/file?id=wjVg/PLOSOne_formatting_sample_main_body.pdf). We used sentence case for headings, cited figures/tables in order, and ensured no citations in the Abstract.

2. Code Sharing: All author-generated code (Python-based, using scikit-learn v1.5, imbalanced-learn, and a custom blockchain-inspired ledger script) is publicly available without restrictions at https://github.com/rubaamff/Hybrid-IDS-Blockchain (DOI: DOI: https://doi.org/10.5281/zenodo.18009378). This repository includes the full preprocessing pipeline, model training scripts, ablation experiments, and ledger implementation, facilitating reproducibility.

3. Funding Statement: We request the following amended Funding Statement: "This research was funded by the Deanship of Postgraduate Studies and Scientific Research at Majmaah University through project number R-2025-XXX. The funders had no role in study design, data collection and analysis, decision to publish, or preparation of the manuscript." Please update the online submission form accordingly. We have removed funding-related text from the Acknowledgments section in the manuscript.

4. Data Availability: All datasets (NSL-KDD and CICIDS2017), source code, and experimental logs are deposited at https://zenodo.org/records/18009378 (DOI: https://doi.org/10.5281/zenodo.18009378)

5. ). This public repository ensures unrestricted access upon publication. No access restrictions apply, as the data are computational and non-sensitive.

6. ORCID iD: The corresponding authors (Shailendra Mishra and Reem Alshenaifi) have validated their ORCID iDs in Editorial Manager (Shailendra MISHRA (0000-0002-0227-7148) - ORCID.).

7. ‪Google Scholar: ID- Dr. Reem Alshenaifi‬ - ‪Google Scholar‬‬‬‬‬, ‪Dr. Shailendra Mishra, Senior Member IEEE‬ - ‪Google Scholar‬‬‬‬‬

Financial Disclosure: No changes needed beyond the amended Funding Statement above.

We believe these revisions fully address the reviewers' concerns and journal requirements, making the manuscript suitable for publication in PLOS ONE. We are happy to provide further clarifications if needed.

Sincerely,

Shailendra Mishra (Corresponding Author)

Reem Alshenaifi (Corresponding Author)

Ruba Ahmed Alfahidah

Department of Computer Engineering/Information Technology

Majmaah University

Saudi Arabia

Email: s.mishra@mu.edu.sa; r.alshenaifi@mu.edu.sa

A. Point-by-Point Response to Reviewers

Reviewer #1: The manuscript presents an ambitious hybrid IDS framework integrating supervised and unsupervised models with PCA, SMOTE, and a blockchain-based auditing layer. The overall concept is potentially innovative, especially in its attempt to unify multiple detection paradigms with tamper-proof logging.

However, several methodological and interpretive issues need clarification.

1. The supervised-only results showing nearly perfect accuracy (≈99.9%) strongly suggest possible data leakage or overfitting.

2. The blockchain implementation is described inconsistently—referencing Ethereum, Hyperledger Fabric, and a private chain—and the reported performance metrics (e.g., 366 blocks/s, 0.09 s/tx, 0 KB storage overhead) do not align with typical blockchain behavior.

3. Details on hyperparameters, PCA/SMOTE impact, ensemble weighting, statistical testing, and threshold selection are insufficient for reproducibility.

4. Additionally, several figures are difficult to interpret, and the writing contains grammar and structural problems that hinder clarity.

5. I recommend strengthening the methodological descriptions, adding ablation studies (e.g., PCA-only, SMOTE-only, ensemble vs. individual models), clarifying the blockchain architecture, and validating the supervised model results to rule out leakage.

6. Improving the writing quality and reorganizing sections for smoother flow would also benefit the manuscript.

7. Ethical and data availability statements appear acceptable, though the authors should ensure there is no overlap with their prior work.

8. Overall, the paper has potential but requires significant revision to meet publication standards in terms of rigor, clarity, and reproducibility.

Response to Reviewer #1

We thank Reviewer #1 for their positive assessment of the manuscript’s potential and for the detailed recommendations regarding methodological rigor, clarity, and reproducibility. We have implemented all suggested changes, including the addition of an ablation study, enhanced methodological descriptions, clarification of the blockchain component, explicit data leakage validation, and overall improvements to the writing quality.

The methodological and interpretive issues have been clarified in the revised manuscript, and brief responses to each comment are provided below.

1. The supervised-only results showing nearly perfect accuracy (≈99.9%) strongly suggest possible data leakage or overfitting.

Response: We agree that such high supervised-only accuracies (≈99.9%) warrant careful validation to rule out data leakage or overfitting. To address this, we have added a dedicated subsection describing a leakage-proof preprocessing pipeline, in which the train-test split is performed first and all transformations (e.g., scaling, SMOTE) are applied only on the training set.

To further ensure robustness, we performed ablation and cross-dataset experiments, summarized in Table 11. These tests show expected performance drops when certain components are removed or when models are evaluated on unseen datasets, confirming that the high accuracies are due to legitimate preprocessing and ensemble learning, not methodological artifacts.

Additionally, Figure 2 has been updated to clearly illustrate the strict separation of training and test data throughout the preprocessing pipeline. These updates, along with the full implementation, are fully reproducible via our project repository: [https://github.com/rubaamff/Hybrid-IDS-Blockchain]

The observed high performance is also consistent with prior literature on similar datasets for binary classification tasks (e.g., [3, 14] report 98–99% accuracy on NSL-KDD and CIC-IDS2017).

(Revised Manuscript, Research Methods -Leakage-Proof Preprocessing Pipeline, Lines 240-294; Figure 2 , page no.-518-519 , Table 11, Lines 848-850).

2. The blockchain implementation is described inconsistently—referencing Ethereum, Hyperledger Fabric, and a private chain—and the reported performance metrics (e.g., 366 blocks/s, 0.09 s/tx, 0 KB storage overhead) do not align with typical blockchain behavior.

Response:

Response: We clarified that the system does not implement a decentralized or consensus-based blockchain. Instead, it uses a lightweight, blockchain-inspired, single-node, append-only audit ledger for tamper-evident forensic logging. All references to Ethereum, Hyperledger, or private chains were removed, and reported metrics were reframed as local logging throughput and latency, consistent with the implementation.

Key Characteristics of the Audit Ledger:

• Single-node, append-only log structure using SHA-256 hash chaining

• No consensus protocol (no PoW, PoS, PBFT, or validator voting)

• No networking or peer-to-peer synchronization

• No mining or block propagation

Implementation Evidence:

• Implemented in blockchain_logger.py with add_block(), calculate_hash(), and verify_chain()

• Fully described in Research Method- Blockchain-Inspired Immutable Audit Logging

• Designed to provide tamper evidence with minimal runtime overhead, not to emulate a decentralized blockchain

Ledger Growth and Storage Overhead:

• Each entry stores timestamp, prediction label, confidence score, and SHA-256 hash (~120 bytes per record)

• For 10,000 entries → ~1–1.2 MB total growth

• Wording updated to “negligible storage overhead” rather than literal 0 KB

Tamper-Resistance Evaluation:

• Controlled integrity tests were performed: blocks were intentionally modified

• The verify_chain() routine detected all alterations, confirming tamper-evident behavior

• This demonstrates tamper evidence, not cryptographic immutability or Byzantine fault tolerance

• Detailed results are shown in Table 3: Tamper-Resistance and Data Integrity Analysis

(Revised Manuscript, Research Method - Blockchain-Inspired Immutable Audit Logging, Lines 335-345, Lightweight Blockchain-Inspired Audit Ledger, Line no-352-359, Table 3: Tamper-Resistance and Data Integrity Analysis (Lines 716–717)

3. Details on hyperparameters, PCA/SMOTE impact, ensemble weighting, statistical testing, and threshold selection are insufficient for reproducibility.

Response: We have expanded the manuscript to provide full details for reproducibility, addressing hyperparameters, PCA/SMOTE impacts, ensemble weighting, statistical testing, and decision threshold selection.

Hyperparameters:

• RF: n_estimators=100, random_state=42

• MLP: hidden_layer_sizes=(100,)

• Binary classification threshold: 0.5

• Ensemble weighting uses cross-validated F1-scores (formula included in the manuscript, Lines 370–394)

Impact of PCA and SMOTE:

• PCA (95% variance retained) improves F1 by ~3%

• SMOTE increases recall by ~5%

• Ablation results are summarized in Table 11 (Lines 848–849)

Verified Preprocessing Pipeline:

• Train–Test Split: 80/20 stratified split

• Feature Scaling: Min–Max normalization fitted only on training data

• Dimensionality Reduction (PCA): Fitted on training set only, then applied to test set

• Class Imbalance Handling (SMOTE): Applied only on the training set after PCA

• Fully implemented in preprocess_data.py and main_notebook.ipynb, publicly available at https://github.com/rubaamff/Hybrid-IDS-Blockchain and Zenodo

Statistical Testing:

• Paired t-tests conducted to evaluate the effect of blockchain-inspired logging

• Metrics: Accuracy, Precision, Recall, F1-score, AUC, False Positive Rate

• Assumptions verified: paired observations, normality (Shapiro–Wilk test), independence, homogeneity of variance

• Results summarized in Table 9, Table 10, and Figure 11 (Lines 402–413, 806–823)

Effect Sizes and Confidence Intervals:

• Cohen’s d: 0.42–0.46 (small-to-moderate effect)

• 95% CIs are narrow, indicating stability and low variance (see Table 10, Lines 812–813)

These additions explicitly quantify the impact of PCA, SMOTE, ensemble weighting, and blockchain-inspired logging, and provide a fully reproducible workflow, demonstrating that reported performance improvements are robust, statistically validated, and not due to methodological artifacts.

4. Additionally, several figures are difficult to interpret, and the writing contains grammar and structural problems that hinder clarity.

Response: We have addressed the clarity and readability issues throughout the manuscript. Specifically:

1. Figures: All figures now include clear titles, descriptive legends, labeled axes, and color bars to facilitate interpretation.

2. Grammar and Style: The manuscript underwent professional editing. Repetitions were removed (e.g., duplicated gaps in the Introduction), terminology was made consistent (e.g., “CIC-IDS2017”), and sentence structures were improved for readability.

3. Flow and Organization: Sections were reorganized for logical progression, including consolidation of blockchain-related details into a dedicated subsection.

These improvements enhance the manuscript’s clarity, readability, and overall presentation.

(Revised Manuscript: All figures updated; general edits applied throughout.)

5. I recommend strengthening the methodological descriptions, adding ablation studies (e.g., PCA-only, SMOTE-only, ensemble vs. individual models), clarifying the blockchain architecture, and validating the supervised model results to rule out leakage.

Response: We have strengthened the methodological descriptions and added ablation studies to address the reviewer’s concerns:

1. Research Methods: The full preprocessing and model pipeline is now described in detail in (Lines 240–344), including hyperparameters, PCA, SMOTE, ensemble weighting, and decision thresholds.

2. Ablation Studies:

• PCA-only: +0.57% accuracy improvement

• SMOTE-only: +5% recall improvement ( Table 11, Lines 848–849)

• Ensemble vs. individual models: +0.39% F1 gain for the full ensemble over individual models

• Ablation results are provided in Table 11 (Lines 848–849)

3. Leakage-Proof Preprocessing Pipeline (Lines 240–329)

4. Blockchain-Inspired Logging: Architecture clarified in Research Methods, emphasizing that this is a lightweight, single-node, hash-chained audit log, not a distributed blockchain,( Lines 352–359)

5. Supervised Model Validation: Potential leakage has been ruled out via the verified preprocessing pipeline and ablation studies. High performance is attributable to legitimate preprocessing and ensemble learning, with no overlap with prior work (e.g., Mishra 2023 [10] focuses on privacy, not ensemble modeling)

These revisions improve methodological clarity, experimental rigor, and reproducibility.

6. Improving the writing quality and reorganizing sections for smoother flow would also benefit the manuscript.

Response: We sincerely thank the reviewer for the positive assessment of the manuscript's potential and for the valuable suggestions on enhancing its presentation and rigor. In this revised version, we have undertaken a comprehensive overhaul of the writing quality and structural organization to improve clarity, logical flow, and readability:

Reorganization for smoother flow: Sections have been streamlined and reordered where appropriate. For instance, the "Key Gaps in Current IDS" and "Research Gap" subsections have been consolidated into a more cohesive "Research Gaps and Contributions" section early in the Introduction to better highlight novelty upfront. The Literature Review has been tightened to focus on thematic comparisons, reducing redundancy. The Proposed Work, Research Methods, and Experimental Setup sections have been merged and restructured into clearer subsections (e.g., Preprocessing Pipeline, Ensemble Integration, Audit Logging) with consistent terminology and cross-references to figures/tables.We appreciate the reviewer's constructive feedback on writing and organization. As detailed ab

Attachment Submitted filename: Responce to Reviewer.docx

# Peer review: Decision Letter 1

**DOI:** 10.1371/journal.pone.0356878.r003

**Authors:** Muhammad Anwar

29 Jan 2026

Dear Dr. Mishra,

Thank you for submitting your manuscript to PLOS ONE. After careful consideration, we feel that it has merit but does not fully meet PLOS ONE’s publication criteria as it currently stands. Therefore, we invite you to submit a revised version of the manuscript that addresses the points raised during the review process.

Please submit your revised manuscript by Mar 15 2026 11:59PM. If you will need more time than this to complete your revisions, please reply to this message or contact the journal office at plosone@plos.org. When you're ready to submit your revision, log on to https://www.editorialmanager.com/pone/ and select the 'Submissions Needing Revision' folder to locate your manuscript file.

- A letter that responds to each point raised by the academic editor and reviewer(s). You should upload this letter as a separate file labeled 'Response to Reviewers'.
- A marked-up copy of your manuscript that highlights changes made to the original version. You should upload this as a separate file labeled 'Revised Manuscript with Track Changes'.
- An unmarked version of your revised paper without tracked changes. You should upload this as a separate file labeled 'Manuscript'.

If applicable, we recommend that you deposit your laboratory protocols in protocols.io to enhance the reproducibility of your results. Protocols.io assigns your protocol its own identifier (DOI) so that it can be cited independently in the future. For instructions see: https://journals.plos.org/plosone/s/submission-guidelines#loc-laboratory-protocols. Additionally, PLOS ONE offers an option for publishing peer-reviewed Lab Protocol articles, which describe protocols hosted on protocols.io. Read more information on sharing protocols at https://plos.org/protocols?utm_medium=editorial-email&utm_source=authorletters&utm_campaign=protocols.

We look forward to receiving your revised manuscript.

Kind regards,

Muhammad Anwar, Ph.D.

Academic Editor

PLOS One

**Journal Requirements:**

1. If the reviewer comments include a recommendation to cite specific previously published works, please review and evaluate these publications to determine whether they are relevant and should be cited. There is no requirement to cite these works unless the editor has indicated otherwise.

2. Please review your reference list to ensure that it is complete and correct. If you have cited papers that have been retracted, please include the rationale for doing so in the manuscript text, or remove these references and replace them with relevant current references. Any changes to the reference list should be mentioned in the rebuttal letter that accompanies your revised manuscript. If you need to cite a retracted article, indicate the article’s retracted status in the References list and also include a citation and full reference for the retraction notice.

[Note: HTML markup is below. Please do not edit.]

Reviewers' comments:

Reviewer's Responses to Questions

**Comments to the Author**

Reviewer #1: All comments have been addressed

Reviewer #2: All comments have been addressed

**********

2. Is the manuscript technically sound, and do the data support the conclusions?

Reviewer #1: Yes

Reviewer #2: Yes

**********

3. Has the statistical analysis been performed appropriately and rigorously?

Reviewer #1: Yes

Reviewer #2: Yes

**********

4. Have the authors made all data underlying the findings in their manuscript fully available?

The PLOS Data policy

Reviewer #1: No

Reviewer #2: Yes

**********

5. Is the manuscript presented in an intelligible fashion and written in standard English?

Reviewer #1: Yes

Reviewer #2: Yes

**********

Reviewer #1: Overall Review

The revised manuscript entitled “A Lightweight Blockchain-Inspired Hybrid Intrusion Detection System with Ensemble Learning for Tamper-Proof Auditing” presents a technically sound and well-executed study addressing an important problem in modern cybersecurity. Following substantial revision, the authors have adequately addressed the major concerns raised in the previous round of review.

The methodological framework is now clearly described and demonstrates appropriate rigor. In particular, the authors have convincingly clarified the preprocessing pipeline to rule out data leakage, provided detailed hyperparameter settings, and justified the use of PCA, SMOTE, and adaptive weighted ensemble voting. The inclusion of comprehensive ablation studies and cross-dataset validation strengthens confidence in the reported performance and demonstrates robustness beyond dataset-specific effects. The statistical analysis has been substantially improved, with clear descriptions of test design, assumption checks, effect sizes, and confidence intervals, making the conclusions well supported by the data.

Importantly, the blockchain-inspired component has been appropriately reframed and clarified. The authors no longer overstate decentralization or consensus properties, instead positioning the mechanism as a lightweight, single-node, hash-chained audit ledger designed for tamper-evident forensic logging. The evaluation of overhead, storage growth, and tamper detection is now transparent, technically realistic, and aligned with the stated design goals.

The manuscript has also benefited from significant improvements in structure, clarity, and language quality. Figures and tables are clearer, redundancy has been reduced, and novelty claims have been moderated to accurately reflect incremental but meaningful contributions relative to prior work. Data and code availability are clearly stated and meet PLOS ONE’s reproducibility requirements.

Overall, the study now meets the journal’s standards for technical correctness, clarity, and transparency. The conclusions are supported by the experimental evidence, and the work makes a useful contribution to the literature on hybrid intrusion detection systems with auditable logging. I therefore consider the manuscript suitable for publication.

Reviewer #2: he authors have performed a commendable and thorough revision of the manuscript. The most significant improvement is the transition from a broad "blockchain" claim to a precisely defined "lightweight blockchain-inspired audit ledger." This transparency, combined with the public release of the full codebase and datasets, significantly enhances the manuscript’s scientific integrity and reproducibility. The technical concerns regarding data leakage and statistical rigor have been addressed with new validation experiments and robust reporting.

2. Methodological Rigor and Technical Validity

Data Leakage Mitigation: The implementation of a strict "leakage-proof" preprocessing pipeline—where splitting occurs before any data-dependent transformations like scaling, PCA, or SMOTE—effectively addresses the primary concern regarding over-optimistic performance. The updated Fig. 2 and the accompanying code references provide the necessary evidence for this correction.

Ensemble Strategy: The use of seven supervised and unsupervised models fused via adaptive weighted voting based on cross-validation F1-scores is a sound approach for handling the high dimensionality of NSL-KDD and CIC-IDS2017 datasets.

Ablation Study: The newly added Table 11 provides crucial insights, quantifying the performance gains from PCA (+0.57% accuracy) and SMOTE (~5% recall boost), justifying their inclusion in the pipeline.

3. Audit Ledger (Blockchain Component)

Refined Scope: The authors successfully clarified that the system uses a single-node, hash-chained ledger for forensic traceability rather than a decentralized consensus-based blockchain. This framing is much more realistic for a lightweight IDS.

Performance Metrics: The reported average latency of ~0.09 s per transaction and the correction from "0 KB" to "negligible" (~1.2 MB for 10,000 entries) storage growth align with expected behavior for local cryptographic hashing.

Tamper Resistance: The inclusion of a dedicated verify_chain() routine and the 100% detection rate of manual record modifications provides strong evidence for the ledger's efficacy in auditability.

4. Statistical Analysis

Rigor: The transition to reporting p-values from paired t-tests, Cohen’s d effect sizes (0.42–0.46), and narrow 95% confidence intervals significantly strengthens the results section.

Interpretation: The observation that the audit layer introduces only a minor, non-significant accuracy drop (from 98.85% to 98.15%) is now statistically substantiated.

5. Clarity and Presentation

Structural Improvements: Removing the unrelated differential equations section and consolidating the "Research Gaps" has significantly improved the manuscript's focus and flow.

Visual Aids: Figures have been updated with bold titles, descriptive legends, and labeled axes, addressing the previous concerns regarding interpretability.

6. Minor Suggestions for Final Polish

Consistency Check: While the authors state in the "Response to Reviewers" that storage overhead is corrected to "negligible," they should ensure that any remaining "0 KB" or "zero persistent growth" phrases in old text blocks (if any) are purged during the final proofing stage.

Novelty Positioning: Ensure the "softened" novelty claims (acknowledging similar hybrid models) are consistently applied in both the Introduction and Conclusion to avoid contradictions.

Final Recommendation: Accept with Minor (Formatting) Revisions. The manuscript now meets PLOS ONE’s requirements for technical rigor, data availability, and reporting standards.

**********

what does this mean?). If published, this will include your full peer review and any attached files.

If you choose “no”, your identity will remain anonymous but your review may still be made public.

**Do you want your identity to be public for this peer review?**  For information about this choice, including consent withdrawal, please see our Privacy Policy

Reviewer #1: **Yes:**  Wenke Du

Reviewer #2: No

**********

[NOTE: If reviewer comments were submitted as an attachment file, they will be attached to this email and accessible via the submission site. Please log into your account, locate the manuscript record, and check for the action link "View Attachments". If this link does not appear, there are no attachment files.]

To ensure your figures meet our technical requirements, please review our figure guidelines: https://journals.plos.org/plosone/s/figures

You may also use PLOS’s free figure tool, NAAS, to help you prepare publication quality figures: https://journals.plos.org/plosone/s/figures#loc-tools-for-figure-preparation.

NAAS will assess whether your figures meet our technical requirements by comparing each figure against our figure specifications.

# Author comment: Author response to Decision Letter 2

**DOI:** 10.1371/journal.pone.0356878.r004

3 Feb 2026

Response to Reviewers Manuscript ID: PONE-D-25-59640R1

Title: A Lightweight Blockchain-Inspired Hybrid Intrusion Detection System with Ensemble Learning for Tamper-Proof Auditing

Dear Dr. Muhammad Anwar and Reviewers,

Greetings!

We sincerely thank both reviewers for their thorough evaluation of the revised manuscript and for the constructive final comments. We are pleased that both Reviewer #1 and Reviewer #2 now consider the major scientific and technical concerns from the previous round to have been adequately addressed, and that the work is viewed as technically sound, well-supported, clearly presented, and suitable for publication in PLOS ONE.

We have carefully considered the two minor suggestions for final polish raised by Reviewer #2 and implemented the corresponding improvements in the submitted files:

Reviewer #2 – Comment 6.1 – Consistency Check

“While the authors state in the "Response to Reviewers" that storage overhead is corrected to "negligible," they should ensure that any remaining "0 KB" or "zero persistent growth" phrases in old text blocks (if any) are purged during the final proofing stage.”

Response: Thank you for this careful observation. We performed a complete keyword search and manual proof-reading pass across the entire manuscript to identify and eliminate any remaining absolute-zero phrasing. All instances of “0 KB”, “zero persistent growth”, “zero memory growth”, and similar expressions have been replaced with technically accurate and consistent terminology such as “negligible”, “minimal”, or “very low and predictable”.

The most relevant corrected passages now read (highlighted in red text in the “Revised Manuscript with Track Changes” file):

• Conclusions section – performance summary paragraph (lines ≈940–944 in the tracked-changes document): “A 0.09 s delay is negligible in enterprise IDS (typical packet rate: 100–10k/s). Memory growth remains negligible and predictable, indicating that edge deployment (e.g., Raspberry Pi, routers) is feasible.”

• Conclusions section – audit mechanism description (lines ≈945–949): “The blockchain-inspired audit mechanism is intentionally lightweight and private, ensuring auditability without the computational overhead of public blockchain platforms, primarily during write operations, while read performance remains largely unaffected. Storage overhead is negligible and stays bounded even under sustained high-throughput alerting.”

These revisions ensure uniform, conservative, and evidence-aligned language throughout the manuscript.

Reviewer #2 – Comment 6.2

Novelty Positioning: Ensure the "softened" novelty claims (acknowledging similar hybrid models) are consistently applied in both the Introduction and Conclusion to avoid contradictions.

Response: We appreciate this valuable suggestion. We have further aligned the tone and phrasing concerning novelty/contribution across the major sections (Introduction, Discussion, and Conclusion) to consistently present our work as a meaningful refinement, unification, and practical integration of existing hybrid detection and lightweight tamper-evident logging concepts ,rather than claiming wholly novel components.

Key updated passages (highlighted in red text in the tracked-changes file) include:

• Introduction – contribution positioning paragraph (lines ≈84–87): “While prior studies have explored hybrid intrusion detection models and blockchain-assisted logging independently, fewer works have examined a tightly integrated, lightweight hash-chained audit mechanism combined with supervised–unsupervised ensemble learning under a unified evaluation framework.”

• Discussion section – blockchain comparison paragraph (lines ≈899–902): “This brings about a considerable decrease in computational and latency overhead while still preserving tamper-evident logging, making the approach more suitable for resource-constrained IoT and edge environments than full consensus-based blockchain frameworks.”

• Conclusion section – contribution summary paragraph (lines ≈924–925): “This study refines and unifies existing hybrid detection and tamper-evident logging concepts into a resource-efficient architecture designed for practical IDS deployment.”

We believe these final adjustments fully resolve the two minor points raised. No other changes were made beyond these targeted consistency improvements.

We confirm that:

• All data underlying the findings are available (code repository link and processed dataset splits are provided in the manuscript).

• The reference list has been checked for completeness and correctness; no retracted articles are cited.

• The marked-up (“Revised Manuscript with Track Changes”) file shows all changes since the previous submission, with the two sets of minor wording updates highlighted in red text for easy identification.

We are grateful for the detailed and constructive feedback throughout the review process, which has substantially improved the clarity, rigor, and scientific integrity of the manuscript.

Thank you again for considering our revised submission. We look forward to your final decision.

Sincerely,

Shailendra Mishra

Corresponding Author

(on behalf of all authors)

Department of Computer Engineering

College of Computer and Information Sciences

Majmaah University, Saudi Arabia

Attachment Submitted filename: Response to Reviewers PONE-D-25-59640R1--Rev 2.docx

# Peer review: Decision Letter 2

**DOI:** 10.1371/journal.pone.0356878.r005

**Authors:** Muhammad Anwar

26 Feb 2026

Dear Dr. Mishra,

Thank you for submitting your manuscript to PLOS ONE. After careful consideration, we feel that it has merit but does not fully meet PLOS ONE’s publication criteria as it currently stands. Therefore, we invite you to submit a revised version of the manuscript that addresses the points raised during the review process.

Please submit your revised manuscript by Apr 12 2026 11:59PM. If you will need more time than this to complete your revisions, please reply to this message or contact the journal office at plosone@plos.org. When you're ready to submit your revision, log on to https://www.editorialmanager.com/pone/ and select the 'Submissions Needing Revision' folder to locate your manuscript file.

- A letter that responds to each point raised by the academic editor and reviewer(s). You should upload this letter as a separate file labeled 'Response to Reviewers'.
- A marked-up copy of your manuscript that highlights changes made to the original version. You should upload this as a separate file labeled 'Revised Manuscript with Track Changes'.
- An unmarked version of your revised paper without tracked changes. You should upload this as a separate file labeled 'Manuscript'.

If applicable, we recommend that you deposit your laboratory protocols in protocols.io to enhance the reproducibility of your results. Protocols.io assigns your protocol its own identifier (DOI) so that it can be cited independently in the future. For instructions see: https://journals.plos.org/plosone/s/submission-guidelines#loc-laboratory-protocols. Additionally, PLOS ONE offers an option for publishing peer-reviewed Lab Protocol articles, which describe protocols hosted on protocols.io. Read more information on sharing protocols at https://plos.org/protocols?utm_medium=editorial-email&utm_source=authorletters&utm_campaign=protocols.

We look forward to receiving your revised manuscript.

Kind regards,

Muhammad Anwar, Ph.D.

Academic Editor

PLOS One

Journal Requirements:

1. If the reviewer comments include a recommendation to cite specific previously published works, please review and evaluate these publications to determine whether they are relevant and should be cited. There is no requirement to cite these works unless the editor has indicated otherwise.

2. Please review your reference list to ensure that it is complete and correct. If you have cited papers that have been retracted, please include the rationale for doing so in the manuscript text, or remove these references and replace them with relevant current references. Any changes to the reference list should be mentioned in the rebuttal letter that accompanies your revised manuscript. If you need to cite a retracted article, indicate the article’s retracted status in the References list and also include a citation and full reference for the retraction notice.

[Note: HTML markup is below. Please do not edit.]

Reviewers' comments:

Reviewer's Responses to Questions

**Comments to the Author**

Reviewer #1: All comments have been addressed

Reviewer #2: All comments have been addressed

**********

2. Is the manuscript technically sound, and do the data support the conclusions?

Reviewer #1: Yes

Reviewer #2: Partly

**********

3. Has the statistical analysis been performed appropriately and rigorously?

Reviewer #1: Yes

Reviewer #2: No

**********

4. Have the authors made all data underlying the findings in their manuscript fully available?

The PLOS Data policy

Reviewer #1: Yes

Reviewer #2: Yes

**********

5. Is the manuscript presented in an intelligible fashion and written in standard English?

Reviewer #1: Yes

Reviewer #2: Yes

**********

Reviewer #1: This manuscript presents a hybrid intrusion detection system (H-IDS) integrating supervised and unsupervised machine learning models within a weighted ensemble framework, combined with a lightweight blockchain-inspired immutable audit mechanism. The work addresses key challenges in IDS research, including class imbalance, dimensionality reduction, ensemble robustness, and tamper-evident logging.

The manuscript is technically sound, methodologically detailed, and reproducible (code and datasets are publicly available). The authors clearly position their contribution as a refinement and integration of existing approaches rather than claiming entirely novel components, which strengthens the scientific integrity of the paper.

Overall, the study is well-executed and suitable for publication after minor clarifications.

Reviewer #2: I appreciate the substantial revisions made in response to the previous review round. The manuscript has improved significantly in clarity, methodological transparency, and positioning of its contributions. In particular, the clarification of the leakage-proof preprocessing pipeline (train–test split prior to scaling, PCA, and SMOTE), the softened novelty claims, and the explicit limitation of the blockchain-inspired component to a single-node hash-chained audit mechanism are positive and constructive improvements.

The integration of supervised and unsupervised models within a weighted ensemble framework is clearly described, and the experimental setup is reproducible, with code and datasets made publicly available. The manuscript is generally well structured and intelligible.

However, a few issues still require clarification before final acceptance:

Statistical Rigor

The paired t-test analysis appears to be conducted with a very small number of repetitions (n = 3, df = 2). While the computations are technically valid, such a small sample size limits statistical power and the robustness of inferential claims. The authors are encouraged either to:

Provide clarification on what constitutes the three paired observations and justify the adequacy of this sample size, or

Temper claims of statistical significance accordingly.

Near-Perfect Supervised-Only Performance

The reported 99.9%+ accuracy on benchmark datasets (NSL-KDD and CIC-IDS2017) is exceptionally high. Although the preprocessing pipeline is described as leakage-resilient, the authors should further clarify how cross-validation, validation splits, and ensemble weighting are strictly isolated from the final test set to eliminate any possibility of inadvertent information leakage.

Blockchain Terminology Precision

The manuscript correctly clarifies that the proposed mechanism is single-node and does not implement decentralized consensus. To avoid possible conceptual confusion, the authors may consider ensuring consistent terminology (e.g., “blockchain-inspired hash-chained audit log”) throughout the manuscript to accurately reflect the security guarantees provided.

These issues are primarily clarification and rigor-related rather than structural flaws. Overall, the work is technically organized and contributes a practical integration of ensemble learning with tamper-evident logging for intrusion detection. With the above clarifications, the manuscript would be suitable for publication.

**********

what does this mean?). If published, this will include your full peer review and any attached files.

If you choose “no”, your identity will remain anonymous but your review may still be made public.

**Do you want your identity to be public for this peer review?**  For information about this choice, including consent withdrawal, please see our Privacy Policy

Reviewer #1: **Yes:**  Wenke Du

Reviewer #2: No

**********

[NOTE: If reviewer comments were submitted as an attachment file, they will be attached to this email and accessible via the submission site. Please log into your account, locate the manuscript record, and check for the action link "View Attachments". If this link does not appear, there are no attachment files.]

To ensure your figures meet our technical requirements, please review our figure guidelines: https://journals.plos.org/plosone/s/figures

You may also use PLOS’s free figure tool, NAAS, to help you prepare publication quality figures: https://journals.plos.org/plosone/s/figures#loc-tools-for-figure-preparation.

NAAS will assess whether your figures meet our technical requirements by comparing each figure against our figure specifications.

# Author comment: Author response to Decision Letter 3

**DOI:** 10.1371/journal.pone.0356878.r006

2 Mar 2026

Response to Reviewers

Manuscript ID: PONE-D-25-59640R2

Title: A Lightweight Blockchain-Inspired Hybrid Intrusion Detection System with Ensemble Learning for Tamper-Proof Auditing

Dear Academic Editor and Reviewers,

We sincerely thank both reviewers for their constructive and insightful evaluations. Reviewer #1’s positive assessment of the manuscript’s technical rigor, reproducibility, and balanced positioning of contributions is greatly appreciated. Reviewer #2’s recognition of the substantial improvements from the previous round, particularly the clarified leakage-safe preprocessing pipeline, moderated novelty claims, and explicit single-node scope of the audit mechanism, has further strengthened the manuscript.

All remaining points have been fully addressed through targeted clarifications, terminology standardization, and cautious statistical interpretation. The revised manuscript (tracked-changes version provided) incorporates all requested refinements while preserving the original scientific contributions.

Below we provide a complete point-by-point response with exact manuscript insertions.

Reviewer #1: This manuscript presents a hybrid intrusion detection system (H-IDS) integrating supervised and unsupervised machine learning models within a weighted ensemble framework, combined with a lightweight blockchain-inspired immutable audit mechanism. The work addresses key challenges in IDS research, including class imbalance, dimensionality reduction, ensemble robustness, and tamper-evident logging.

The manuscript is technically sound, methodologically detailed, and reproducible (code and datasets are publicly available). The authors clearly position their contribution as a refinement and integration of existing approaches rather than claiming entirely novel components, which strengthens the scientific integrity of the paper.

Response:

We sincerely thank the reviewer for this encouraging assessment.

Reviewer #2: I appreciate the substantial revisions made in response to the previous review round. The manuscript has improved significantly in clarity, methodological transparency, and positioning of its contributions. In particular, the clarification of the leakage-proof preprocessing pipeline (train–test split prior to scaling, PCA, and SMOTE), the softened novelty claims, and the explicit limitation of the blockchain-inspired component to a single-node hash-chained audit mechanism are positive and constructive improvements.

The integration of supervised and unsupervised models within a weighted ensemble framework is clearly described, and the experimental setup is reproducible, with code and datasets made publicly available. The manuscript is generally well structured and intelligible.

However, a few issues still require clarification before final acceptance:

1- Statistical Rigor

The paired t-test analysis appears to be conducted with a very small number of repetitions (n = 3, df = 2). While the computations are technically valid, such a small sample size limits statistical power and the robustness of inferential claims. The authors are encouraged either to:

1-Provide clarification on what constitutes the three paired observations and justify the adequacy of this sample size, or

Response:

We thank the reviewer for the constructive evaluation and clear guidance for final refinement. Each remaining issue has been addressed as detailed below.

We fully agree that n = 3 constrains inferential robustness. The paired observations represent three independent full-pipeline experimental executions comparing pre-ledger and post-ledger performance under identical configurations. Each execution includes preprocessing, model training, ensemble inference, and ledger integration, conducted with distinct random seeds to ensure independence and reproducibility.

To address the reviewer’s concern, we have:

• clarified the origin of paired observations

• explicitly stated statistical limitations

• tempered claims from “statistically significant” to cautious interpretation

• reported effects as indicative trends

Manuscript Insert LINE NO (971-977) - (Statistical Analysis)

The paired t-test comparing pre- and post-audit performance derives from three independent executions of the complete training–evaluation pipeline (preprocessing, model training, ensemble inference, and ledger integration) conducted with different random seeds. These executions constitute the paired observations (n = 3, df = 2). Although the paired t-test is mathematically valid for this sample size, the limited number of repetitions reduces statistical power; therefore, results are interpreted as indicative of consistent performance trends rather than definitive population-level inference. Absolute metric differences and effect magnitudes are reported alongside p-values.

Manuscript Insert, LINE NO (938-941) - (Ledger Impact)

The audit mechanism produced consistent reductions in false positives across all evaluated configurations. While paired comparisons indicate an improvement trend (paired t-test, n = 3, p < 0.05), the small number of observations limits statistical power; thus, results are interpreted as indicative rather than conclusive evidence of improvement.

Manuscript Insert, LINE NO (25-26) - Abstract

Original: “statistically significant reductions in false positives.”

Revised: observed reductions in false positives (paired t-test, n = 3, p < 0.05,).

2-Temper claims of statistical significance accordingly.

Response:

We agree with the reviewer that, given the limited number of paired observations (n = 3), strong inferential claims are not warranted. Accordingly, all statements implying definitive statistical significance have been revised to reflect cautious interpretation. Throughout the manuscript, wording such as “statistically significant improvement” has been replaced with neutral phrasing (e.g., “observed reduction,” “improvement trend,” or “paired t-test, n = 3”). We also explicitly state the limited statistical power and interpret the results as indicative rather than confirmatory.

Revisions made:

• Abstract:

“statistically significant reductions” →

“observed reductions (paired t-test, n = 3,p < 0.05)” (Line 25-26)

• Research Methods – Statistical Analysis (line 494-496):

As the paired t-test is mathematically correct for n = 3, the small number of repetitions means that the results are viewed as indicative of trends rather than making any statistical inference on the population.

Results – Ledger Impact (Line 938-941)

The audit mechanism has consistently reduced false positives in all configurations under evaluation. Although the paired comparison shows a trend of improvement (paired t-test, n = 3, p < 0.05), the low number of samples makes the results more indicative than conclusive of improvement.

These revisions appropriately temper statistical claims while preserving the descriptive reporting of observed performance differences.

3-Near-Perfect Supervised-Only Performance

The reported 99.9%+ accuracy on benchmark datasets (NSL-KDD and CIC-IDS2017) is exceptionally high. Although the preprocessing pipeline is described as leakage-resilient, the authors should further clarify how cross-validation, validation splits, and ensemble weighting are strictly isolated from the final test set to eliminate any possibility of inadvertent information leakage.

Response:

We appreciate this important safeguard check. The pipeline already enforced strict isolation; however, this was not sufficiently explicit. The manuscript now unequivocally states that:

• train/test split occurs first

• PCA fitted only on training data

• SMOTE applied only on training data

• cross-validation confined to training partition

• ensemble weights derived solely from training CV

• test set used exactly once for final evaluation

These clarifications have been added to Methods, Ensemble Integration, and Experimental Setup.

Manuscript Insert, LINE NO (301-309), Research Methods (Leakage-Safe Pipeline)

To eliminate any possibility of information leakage, all preprocessing, model selection, and weighting steps were confined strictly to the training data. The workflow proceeds as follows: (1) the dataset is first partitioned into fixed training and test sets; (2) PCA is fitted exclusively on the training set and then applied to the test set using the learned transformation; (3) SMOTE is applied only to the training data after PCA; (4) cross-validation, hyperparameter tuning, and ensemble weight estimation are performed solely within the training partition; (5) the held-out test set is accessed once for final performance evaluation. No stage of model fitting, weighting, or threshold selection uses information from the test data.

Manuscript Insert, LINE NO (395-397), Ensemble Integration

Ensemble weights were derived solely from macro-F1 scores obtained during cross-validation on the training data; no test-set information influenced weight assignment or decision thresholds.

Manuscript Insert, LINE NO (608-610), Experimental Setup

The final ensemble model was tested only once on the untouched test set after the completion of all the training, validation, and calibration procedures.

Manuscript Insert, LINE NO (1028-1032), Discussion (Performance Interpretation)

The near-perfect accuracy observed in the supervised-only ensemble should be interpreted in the context of leakage-resilient preprocessing and fixed benchmark datasets. Because dimensionality reduction, balancing, and weight estimation were confined entirely to the training partition, the reported performance does not arise from inadvertent test-set information leakage.

4-Blockchain Terminology Precision

The manuscript correctly clarifies that the proposed mechanism is single-node and does not implement decentralized consensus. To avoid possible conceptual confusion, the authors may consider ensuring consistent terminology (e.g., “blockchain-inspired hash-chained audit log”) throughout the manuscript to accurately reflect the security guarantees provided.

Response:

We fully agree and have standardized terminology throughout the manuscript to:

“blockchain-inspired hash-chained audit log (single-node)”

All ambiguous terms (“blockchain ledger,” “private blockchain,” “blockchain-based”) have been replaced to avoid implying decentralization or consensus.

Global Terminology Revision

• “blockchain ledger” → blockchain-inspired hash-chained audit log

• “private blockchain” → single-node hash-chained audit log

• “blockchain-based” → blockchain-inspired

Manuscript Insert, LINE NO (443-446), Lightweight Blockchain-Inspired Audit Ledger

The proposed mechanism is a single-node blockchain-inspired hash-chained audit log that links detection records using SHA-256 hashes without decentralized consensus, mining, peer-to-peer networking, or smart contracts. Its purpose is tamper-evident sequencing of intrusion-detection events rather than distributed trust establishment.

Manuscript Insert, LINE NO (19-22), Abstract

A lightweight blockchain-inspired hash-chained audit log provides tamper-evident logging of detection events in a single-node deployment without decentralized consensus.

The revised manuscript now incorporates:

• explicit clarification and cautious interpretation of paired t-test results (n = 3)

• unambiguous statements confirming zero test-set leakage across all pipeline stages

• uniform terminology: “blockchain-inspired hash-chained audit log”

• strengthened methodological transparency and reproducibility

We sincerely thank the reviewers and Academic Editor for their valuable guidance, which has substantially improved the clarity, rigor, and precision of this work. We believe the manuscript is now fully suitable for publication in PLOS ONE and remain available for any further clarification if required.

Sincerely,

Shailendra Mishra (on behalf of all authors)

Corresponding author: Reem Alshenaifi

Email: r.alshnaifi@mu.edu.sa

Attachment Submitted filename: Response to Reviewer March.docx

# Peer review: Decision Letter 3

**DOI:** 10.1371/journal.pone.0356878.r007

**Authors:** Muhammad Anwar

16 Mar 2026

Dear Dr. Mishra,

Thank you for submitting your manuscript to PLOS ONE. After careful consideration, we feel that it has merit but does not fully meet PLOS ONE’s publication criteria as it currently stands. Therefore, we invite you to submit a revised version of the manuscript that addresses the points raised during the review process.

Please submit your revised manuscript by Apr 30 2026 11:59PM. If you will need more time than this to complete your revisions, please reply to this message or contact the journal office at plosone@plos.org. When you're ready to submit your revision, log on to https://www.editorialmanager.com/pone/ and select the 'Submissions Needing Revision' folder to locate your manuscript file.

- A letter that responds to each point raised by the academic editor and reviewer(s). You should upload this letter as a separate file labeled 'Response to Reviewers'.
- A marked-up copy of your manuscript that highlights changes made to the original version. You should upload this as a separate file labeled 'Revised Manuscript with Track Changes'.
- An unmarked version of your revised paper without tracked changes. You should upload this as a separate file labeled 'Manuscript'.

If applicable, we recommend that you deposit your laboratory protocols in protocols.io to enhance the reproducibility of your results. Protocols.io assigns your protocol its own identifier (DOI) so that it can be cited independently in the future. For instructions see: https://journals.plos.org/plosone/s/submission-guidelines#loc-laboratory-protocols. Additionally, PLOS ONE offers an option for publishing peer-reviewed Lab Protocol articles, which describe protocols hosted on protocols.io. Read more information on sharing protocols at https://plos.org/protocols?utm_medium=editorial-email&utm_source=authorletters&utm_campaign=protocols.

We look forward to receiving your revised manuscript.

Kind regards,

Muhammad Anwar, Ph.D.

Academic Editor

PLOS One

Journal Requirements:

1. If the reviewer comments include a recommendation to cite specific previously published works, please review and evaluate these publications to determine whether they are relevant and should be cited. There is no requirement to cite these works unless the editor has indicated otherwise.

2. Please review your reference list to ensure that it is complete and correct. If you have cited papers that have been retracted, please include the rationale for doing so in the manuscript text, or remove these references and replace them with relevant current references. Any changes to the reference list should be mentioned in the rebuttal letter that accompanies your revised manuscript. If you need to cite a retracted article, indicate the article’s retracted status in the References list and also include a citation and full reference for the retraction notice.

[Note: HTML markup is below. Please do not edit.]

Reviewer's Responses to Questions

**Comments to the Author**

Reviewer #1: All comments have been addressed

Reviewer #2: (No Response)

**********

2. Is the manuscript technically sound, and do the data support the conclusions?

Reviewer #1: Yes

Reviewer #2: Partly

**********

3. Has the statistical analysis been performed appropriately and rigorously?

Reviewer #1: Yes

Reviewer #2: No

**********

4. Have the authors made all data underlying the findings in their manuscript fully available?

The PLOS Data policy

Reviewer #1: Yes

Reviewer #2: Yes

**********

5. Is the manuscript presented in an intelligible fashion and written in standard English?

Reviewer #1: Yes

Reviewer #2: Yes

**********

Reviewer #1: well writen compared with last time considering the rigorous template and the bullet point to prove the academic theis

Reviewer #2: Thank you for the careful revision of the manuscript and the detailed responses to the reviewers’ comments. The revised version has improved in clarity and methodological transparency. In particular, the clarification of the leakage-safe preprocessing pipeline, the moderation of statistical claims, and the consistent terminology describing the blockchain-inspired hash-chained audit log have strengthened the manuscript.

The methodology is clearly described, and the availability of the source code and datasets improves the reproducibility of the study. The explanation of the training–test separation and ensemble weighting procedure also addresses the earlier concerns regarding potential data leakage.

While the statistical comparison is based on a limited number of repetitions, the authors appropriately acknowledge this limitation and present the results cautiously. Overall, the manuscript now provides a technically sound and clearly presented study.

I believe the manuscript is suitable for publication in PLOS ONE.

**********

what does this mean?). If published, this will include your full peer review and any attached files.

If you choose “no”, your identity will remain anonymous but your review may still be made public.

**Do you want your identity to be public for this peer review?**  For information about this choice, including consent withdrawal, please see our Privacy Policy

Reviewer #1: No

Reviewer #2: No

**********

[NOTE: If reviewer comments were submitted as an attachment file, they will be attached to this email and accessible via the submission site. Please log into your account, locate the manuscript record, and check for the action link "View Attachments". If this link does not appear, there are no attachment files.]

To ensure your figures meet our technical requirements, please review our figure guidelines: https://journals.plos.org/plosone/s/figures

You may also use PLOS’s free figure tool, NAAS, to help you prepare publication quality figures: https://journals.plos.org/plosone/s/figures#loc-tools-for-figure-preparation.

NAAS will assess whether your figures meet our technical requirements by comparing each figure against our figure specifications.

# Author comment: Author response to Decision Letter 4

**DOI:** 10.1371/journal.pone.0356878.r008

17 Mar 2026

Response to Reviewers

PONE-D-25-59640R3

A Lightweight Blockchain-Inspired Hybrid Intrusion Detection System with Ensemble Learning for Tamper-Proof Auditing

PLOS One

Journal Requirements:

1. If the reviewer comments include a recommendation to cite specific previously published works, please review and evaluate these publications to determine whether they are relevant and should be cited. There is no requirement to cite these works unless the editor has indicated otherwise.

Response-

We thank the reviewers for their valuable suggestions, including recommendations to consider citing additional previously published works. We have carefully reviewed the suggested references and evaluated their relevance to our study. Relevant works have been incorporated where they meaningfully support the context and discussion of the manuscript, while those not directly aligned with the scope of this study were not included, in accordance with the journal guidelines. We also appreciate the positive feedback from both reviewers. We are grateful for Reviewer #1’s acknowledgment of the improved structure and academic clarity, and Reviewer #2’s detailed evaluation highlighting enhancements in methodological transparency, data handling, and reproducibility.

We have addressed all comments accordingly and believe the manuscript has been significantly strengthened. We thank the reviewers and the editor for their constructive feedback and consideration.

2. Please review your reference list to ensure that it is complete and correct. If you have cited papers that have been retracted, please include the rationale for doing so in the manuscript text, or remove these references and replace them with relevant current references. Any changes to the reference list should be mentioned in the rebuttal letter that accompanies your revised manuscript. If you need to cite a retracted article, indicate the article’s retracted status in the References list and also include a citation and full reference for the retraction notice.

Response- We have carefully reviewed the entire reference list(1–40) to ensure completeness, accuracy, and relevance in accordance with the journal guidelines. All cited works were verified for their publication status. To the best of our knowledge, none of the references included in the manuscript have been retracted.

Additionally, we ensured that all references are current, relevant to the scope of the study, and appropriately cited within the manuscript. No retracted articles were identified; therefore, no replacements or justifications were required.

We confirm that the reference list complies with the journal’s requirements.

[Note: HTML markup is below. Please do not edit.]

Reviewer's Responses to Questions

Comments to the Author

1. If the authors have adequately addressed your comments raised in a previous round of review and you feel that this manuscript is now acceptable for publication, you may indicate that here to bypass the “Comments to the Author” section, enter your conflict of interest statement in the “Confidential to Editor” section, and submit your "Accept" recommendation.

Reviewer #1: All comments have been addressed

Reviewer #2: (No Response)

Response- We thank the reviewers for their time and valuable feedback. We are pleased that Reviewer #1 has confirmed that all previously raised comments have been adequately addressed. We also appreciate Reviewer #2’s earlier constructive suggestions, which helped us improve the clarity, methodology, and overall quality of the manuscript.

We believe that all concerns have been resolved and that the manuscript is now suitable for publication.

2. Is the manuscript technically sound, and do the data support the conclusions?

The manuscript must describe a technically sound piece of scientific research with data that supports the conclusions. Experiments must have been conducted rigorously, with appropriate controls, replication, and sample sizes. The conclusions must be drawn appropriately based on the data presented.

Reviewer #1: Yes

Reviewer #2: Partly

Response-

3. Has the statistical analysis been performed appropriately and rigorously?

Reviewer #1: Yes

Reviewer #2: No

Response:

We thank the reviewers for their careful evaluation of the statistical analysis. We are encouraged by Reviewer #1’s positive assessment and have further strengthened the manuscript to address Reviewer #2’s concern regarding statistical rigor.

In the revised manuscript, we have clarified and improved the statistical methodology as follows:

• We explicitly described the paired t-test setup, including the use of independent experimental runs (n = 3) with different random seeds, ensuring reproducibility and proper pairing of observations.

• We verified and reported key assumptions of the paired t-test, including normality , independence of observations, and variance consistency.

• The p-values with effect size (Cohen’s d) and confidence interval estimates to provide a more comprehensive statistical interpretation.

• We clearly acknowledged the limitation of the small sample size (n = 3) and explicitly stated that the statistical results should be interpreted as indicative trends rather than definitive population-level conclusions.

These revisions improve the transparency, correctness, and interpretation of the statistical analysis. While the paired t-test is mathematically valid for the given experimental design, we have carefully moderated our claims and aligned conclusions with the available evidence.

We believe the statistical analysis is now appropriately conducted, clearly explained, and consistent with the scope of the study.

4. Have the authors made all data underlying the findings in their manuscript fully available?

The PLOS Data policy requires authors to make all data underlying the findings described in their manuscript fully available without restriction, with rare exception (please refer to the Data Availability Statement in the manuscript PDF file). The data should be provided as part of the manuscript or its supporting information, or deposited to a public repository. For example, in addition to summary statistics, the data points behind means, medians and variance measures should be available. If there are restrictions on publicly sharing data—e.g. participant privacy or use of data from a third party—those must be specified.

Reviewer #1: Yes

Reviewer #2: Yes

Response:

We thank the reviewers for confirming that the data underlying the findings are fully available. All datasets used in this study (NSL-KDD and CIC-IDS2017) are publicly accessible and have been properly referenced in the manuscript.

In addition, we have ensured that all relevant data, preprocessing steps, and implementation details are clearly described, and the source code has been made publicly available to support reproducibility.

We confirm that the manuscript complies with the PLOS Data Availability policy.

5. Is the manuscript presented in an intelligible fashion and written in standard English?

PLOS ONE does not copyedit accepted manuscripts, so the language in submitted articles must be clear, correct, and unambiguous. Any typographical or grammatical errors should be corrected at revision, so please note any specific errors here.

Reviewer #1: Yes

Reviewer #2: Yes

Response:

We thank the reviewers for their positive assessment of the manuscript’s clarity and language. We have carefully proofread the revised manuscript to ensure that it is written in clear, correct, and standard English, and that all typographical and grammatical issues have been addressed.

We confirm that the manuscript is presented in an intelligible and unambiguous manner in accordance with the journal’s requirements.

6. Review Comments to the Author

Please use the space provided to explain your answers to the questions above. You may also include additional comments for the author, including concerns about dual publication, research ethics, or publication ethics. (Please upload your review as an attachment if it exceeds 20,000 characters)

Reviewer #1: well writen compared with last time considering the rigorous template and the bullet point to prove the academic thesis

Response:

We sincerely thank the reviewer for the positive feedback and for recognizing the improvements in the manuscript. We appreciate the acknowledgment of the enhanced structure, adherence to the rigorous template, and the clearer presentation of the academic contributions.

We are grateful for the reviewer’s guidance throughout the revision process, which has helped us significantly improve the overall quality and clarity of the manuscript.

Reviewer #2: Thank you for the careful revision of the manuscript and the detailed responses to the reviewers’ comments. The revised version has improved in clarity and methodological transparency. In particular, the clarification of the leakage-safe preprocessing pipeline, the moderation of statistical claims, and the consistent terminology describing the blockchain-inspired hash-chained audit log have strengthened the manuscript.

The methodology is clearly described, and the availability of the source code and datasets improves the reproducibility of the study. The explanation of the training–test separation and ensemble weighting procedure also addresses the earlier concerns regarding potential data leakage.

While the statistical comparison is based on a limited number of repetitions, the authors appropriately acknowledge this limitation and present the results cautiously. Overall, the manuscript now provides a technically sound and clearly presented study.

I believe the manuscript is suitable for publication in PLOS ONE.

Response :

We sincerely thank the reviewer for the thorough evaluation and for recognizing the improvements made in the revised manuscript. We are grateful for the positive feedback regarding the enhanced clarity, methodological transparency, and overall presentation of the study.

We appreciate the acknowledgment of the leakage-safe preprocessing pipeline, the careful moderation of statistical claims, and the consistent description of the blockchain-inspired hash-chained audit log. We are also pleased that the explanations of training–test separation and ensemble weighting have addressed the earlier concerns regarding potential data leakage.

We acknowledge the reviewer’s note regarding the limited number of repetitions and confirm that this limitation has been clearly stated and carefully considered in the interpretation of results.

We are thankful for the reviewer’s support and for considering the manuscript suitable for publication in PLOS ONE.

We sincerely thank the reviewers and the editor for their time, constructive feedback, and valuable suggestions, which have significantly improved the quality and clarity of our manuscript. We greatly appreciate their consideration and support, and we look forward to the final decision.

Shailendra Mishra

(on the behalf of All authors)

Attachment Submitted filename: Response to Reviewers PLOOne 18 March.docx

# Peer review: Decision Letter 4

**DOI:** 10.1371/journal.pone.0356878.r009

**Authors:** Rizik Al-Sayyed

27 May 2026

Dear Dr. Mishra,

Thank you for submitting your manuscript to PLOS ONE. After careful consideration, we feel that it has merit but does not fully meet PLOS ONE’s publication criteria as it currently stands. Therefore, we invite you to submit a revised version of the manuscript that addresses the points raised during the review process.

Please submit your revised manuscript by Jul 11 2026 11:59PM. If you will need more time than this to complete your revisions, please reply to this message or contact the journal office at plosone@plos.org. When you're ready to submit your revision, log on to https://www.editorialmanager.com/pone/ and select the 'Submissions Needing Revision' folder to locate your manuscript file.

- A letter that responds to each point raised by the academic editor and reviewer(s). You should upload this letter as a separate file labeled 'Response to Reviewers'.
- A marked-up copy of your manuscript that highlights changes made to the original version. You should upload this as a separate file labeled 'Revised Manuscript with Track Changes'.
- An unmarked version of your revised paper without tracked changes. You should upload this as a separate file labeled 'Manuscript'.

If you would like to make changes to your financial disclosure, please include your updated statement in your cover letter. Guidelines for resubmitting your figure files are available below the reviewer comments at the end of this letter.

We look forward to receiving your revised manuscript.

Kind regards,

Rizik M. H. Al-Sayyed, Ph.D.

Academic Editor

PLOS One

Journal Requirements:

1. If the reviewer comments include a recommendation to cite specific previously published works, please review and evaluate these publications to determine whether they are relevant and should be cited. There is no requirement to cite these works unless the editor has indicated otherwise.

2. Please review your reference list to ensure that it is complete and correct. If you have cited papers that have been retracted, please include the rationale for doing so in the manuscript text, or remove these references and replace them with relevant current references. Any changes to the reference list should be mentioned in the rebuttal letter that accompanies your revised manuscript. If you need to cite a retracted article, indicate the article’s retracted status in the References list and also include a citation and full reference for the retraction notice.

Additional Editor Comments:

The manuscript is technically sound, methodologically rigorous, and presents a valuable H-IDS framework combining high detection accuracy with blockchain-inspired auditability. The study is suitable for publication after minor revisions, mainly requiring brief clarification on scalability under high-volume attacks, handling of encrypted traffic, and provision of code/data availability details to support reproducibility. No major methodological or ethical concerns were identified.

[Note: HTML markup is below. Please do not edit.]

Reviewer's Responses to Questions

**Comments to the Author**

Reviewer #1: All comments have been addressed

Reviewer #2: (No Response)

**********

2. Is the manuscript technically sound, and do the data support the conclusions?

Reviewer #1: Yes

Reviewer #2: Yes

**********

3. Has the statistical analysis been performed appropriately and rigorously?

Reviewer #1: Yes

Reviewer #2: Yes

**********

4. Have the authors made all data underlying the findings in their manuscript fully available?

The PLOS Data policy

Reviewer #1: Yes

Reviewer #2: No

**********

5. Is the manuscript presented in an intelligible fashion and written in standard English?

Reviewer #1: Yes

Reviewer #2: Yes

**********

Reviewer #1: The revised manuscript presents a substantial improvement over previous versions and now provides a clear, well-structured, and technically sound contribution. The authors have adequately addressed the concerns raised in earlier review rounds, particularly with respect to methodological transparency, data handling, and clarity of presentation.

The proposed hybrid intrusion detection system (H-IDS), integrating supervised and unsupervised ensemble learning with a lightweight blockchain-inspired audit mechanism, is well-motivated and clearly described. The inclusion of a leakage-safe preprocessing pipeline, explicit training–test separation, and adaptive weighted ensemble design significantly strengthens the technical rigor of the work.

The statistical analysis has also been improved. Although the number of experimental repetitions remains limited (n = 3), the authors have appropriately clarified assumptions, reported effect sizes, and moderated their claims. This transparent discussion of limitations is appropriate and aligns with good scientific practice.

Data availability and reproducibility are well addressed, with datasets, preprocessing details, and source code made publicly accessible. The manuscript is written in clear and standard English, and the overall presentation is coherent and easy to follow.

Overall, the study provides a technically sound and reproducible contribution with practical relevance to intrusion detection and auditability in cybersecurity systems. The combination of ensemble learning and tamper-evident logging is novel within the presented scope.

Recommendation: Accept for publication.

Reviewer #2: The manuscript presents a technically sound and well-executed study on a Hybrid Intrusion Detection System (H-IDS) that integrates machine learning ensembles with a blockchain-inspired auditing layer. The core strength of the work lies in its dual focus: achieving high detection accuracy (98%+) while providing a verifiable, tamper-proof forensic trail for network events. The methodology demonstrates high academic rigor, particularly in its handling of data preprocessing to prevent leakage. 1. Technical Rigor and MethodologyThe authors have established a robust pipeline for evaluating their H-IDS: Data Integrity: The use of Min-Max Scaling, PCA, and SMOTE is handled correctly by applying these transformations only after the train-test split. This ensures that the results are not artificially inflated by data leakage. Ensemble Design: The combination of seven diverse models—ranging from supervised learners like CatBoost and DNN to unsupervised anomaly detectors like Autoencoders—is well-justified for capturing both known and zero-day threats. Auditability: The integration of a SHA-256 hash-chaining mechanism for logging is a significant contribution. The "Tamper Evidence" analysis effectively proves the system's ability to identify unauthorized modifications to historical logs. 2. Statistical AnalysisThe statistical validation is appropriate for a study of this nature: The use of 5-fold cross-validation ensures that the performance metrics are stable and generalizable. The application of a paired t-test ($p < 0.05$) provides the necessary formal evidence that the hybrid ensemble's performance gains are statistically significant compared to individual base learners. 3. Areas for Improvement & RecommendationsWhile the manuscript is strong, addressing the following minor points would enhance its impact:Scalability Concerns: The throughput is reported at 210 blocks per second. The authors should include a brief discussion on how the system would behave under extreme traffic conditions, such as a volumetric DDoS attack, where log generation might exceed this rate. Encrypted Traffic: Given that the majority of modern network traffic is encrypted, it would be beneficial for the authors to clarify if the H-IDS operates on packet headers or if it requires decrypted payloads. Reproducibility: To align with PLOS ONE's commitment to open science, the authors are encouraged to provide a link to a public repository containing the custom weighting logic and the ledger implementation. 4. Ethics and Dual PublicationResearch Ethics: No ethical concerns were identified. The study utilizes public benchmarks (NSL-KDD and CIC-IDS2017) and does not involve human subjects or sensitive personal data. Publication Ethics: There are no indicators of dual publication or self-plagiarism. The work appears original and provides a distinct contribution to the intersection of cybersecurity and distributed ledger principles. Final Recommendation: Weak AcceptThe manuscript is a strong candidate for publication following minor clarifications regarding scalability and data availability

**********

what does this mean?). If published, this will include your full peer review and any attached files.

If you choose “no”, your identity will remain anonymous but your review may still be made public.

**Do you want your identity to be public for this peer review?**  For information about this choice, including consent withdrawal, please see our Privacy Policy

Reviewer #1: **Yes:**  Wenke Du

Reviewer #2: No

**********

[NOTE: If reviewer comments were submitted as an attachment file, they will be attached to this email and accessible via the submission site. Please log into your account, locate the manuscript record, and check for the action link "View Attachments". If this link does not appear, there are no attachment files.]

To ensure your figures meet our technical requirements, please review our figure guidelines: https://journals.plos.org/plosone/s/figures

You may also use PLOS’s free figure tool, NAAS, to help you prepare publication quality figures: https://journals.plos.org/plosone/s/figures#loc-tools-for-figure-preparation.

NAAS will assess whether your figures meet our technical requirements by comparing each figure against our figure specifications.

# Author comment: Author response to Decision Letter 5

**DOI:** 10.1371/journal.pone.0356878.r010

29 May 2026

Point to Point -RESPONSE TO ACADEMIC EDITOR & REVIEWER# 2 COMMENTS

Additional Editor Comments:

The manuscript is technically sound, methodologically rigorous, and presents a valuable H-IDS framework combining high detection accuracy with blockchain-inspired auditability. The study is suitable for publication after minor revisions, mainly requiring brief clarification on scalability under high-volume attacks, handling of encrypted traffic, and provision of code/data availability details to support reproducibility. No major methodological or ethical concerns were identified.

Response:

We sincerely thank the Academic Editor for the positive evaluation of our work and for identifying three important areas where additional clarification would strengthen the manuscript.

To address these recommendations:

1. Scalability under high-volume attacks -Scalability under high volume attack situations has been improved by having a separate discussion on the scalability under volumetric DDoS attacks, the throughput of the blockchain-based audit ledger, the behavior under volumetric DDoS attacks, and potential future scalability improvements such as asynchronous event buffering, batching of events, distributed ledger partitioning, and stream processing. The discussion now clearly indicates that the detection and audit modules are independent, enabling scalability without impacting the detection module.

2. Handling of encrypted traffic-Compatibility of the H-IDS with encrypted traffic was addressed through the explicit specification that the H-IDS works solely with statistical and metadata-based features derived from packet headers and flows, without relying on the content of the payload at the application layer. Therefore, the approach can be used effectively with encrypted flows utilizing TLS/SSL encryption. It is also noted that for application layer attacks performed using encrypted flows, other measures might need to be implemented.

3. Provision of code/data availability details to support reproducibility- The reproducibility of the study and compliance with open science principles have been enhanced through the explicit inclusion of the public GitHub repository (https://github.com/rubaamff/Hybrid-IDS-Blockchain) and the DOI-registered Zenodo archive (https://doi.org/10.5281/zenodo.18009378), ensuring full reproducibility, transparency, and long-term accessibility in accordance with PLOS ONE open science policy. The release includes the complete source code, data pre-processing pipeline, ensemble weighting algorithm, blockchain-based audit logging components, experimental configurations, and all other artifacts necessary to reproduce the reported results.

Also, all the descriptions of manuscripts have been checked to ensure that they comply with the audit technique that is used in the system, which is a minimalistic hash chaining audit ledger inspired by blockchains and implemented using Python code.The manuscript has been revised accordingly.

RESPONSE TO REVIEWER #2

Comments:

Reviewer #2: The manuscript presents a technically sound and well-executed study on a Hybrid Intrusion Detection System (H-IDS) that integrates machine learning ensembles with a blockchain-inspired auditing layer. The core strength of the work lies in its dual focus: achieving high detection accuracy (98%+) while providing a verifiable, tamper-proof forensic trail for network events. The methodology demonstrates high academic rigor, particularly in its handling of data preprocessing to prevent leakage.

1. Technical Rigor and Methodology The authors have established a robust pipeline for evaluating their H-IDS: Data Integrity: The use of Min-Max Scaling, PCA, and SMOTE is handled correctly by applying these transformations only after the train-test split. This ensures that the results are not artificially inflated by data leakage. Ensemble Design: The combination of seven diverse models—ranging from supervised learners like CatBoost and DNN to unsupervised anomaly detectors like Autoencoders—is well-justified for capturing both known and zero-day threats. Auditability: The integration of a SHA-256 hash-chaining mechanism for logging is a significant contribution. The "Tamper Evidence" analysis effectively proves the system's ability to identify unauthorized modifications to historical logs.

2. Statistical AnalysisThe statistical validation is appropriate for a study of this nature: The use of 5-fold cross-validation ensures that the performance metrics are stable and generalizable. The application of a paired t-test ($p < 0.05$) provides the necessary formal evidence that the hybrid ensemble's performance gains are statistically significant compared to individual base learners.

3. Areas for Improvement & Recommendations -While the manuscript is strong, addressing the following minor points would enhance its impact: Scalability Concerns: The throughput is reported at 210 blocks per second. The authors should include a brief discussion on how the system would behave under extreme traffic conditions, such as a volumetric DDoS attack, where log generation might exceed this rate. Encrypted Traffic: Given that the majority of modern network traffic is encrypted, it would be beneficial for the authors to clarify if the H-IDS operates on packet headers or if it requires decrypted payloads. Reproducibility: To align with PLOS ONE's commitment to open science, the authors are encouraged to provide a link to a public repository containing the custom weighting logic and the ledger implementation.

4. Ethics and Dual Publication Research Ethics: No ethical concerns were identified. The study utilizes public benchmarks (NSL-KDD and CIC-IDS2017) and does not involve human subjects or sensitive personal data. Publication Ethics: There are no indicators of dual publication or self-plagiarism. The work appears original and provides a distinct contribution to the intersection of cybersecurity and distributed ledger principles. Final Recommendation: Weak Accept The manuscript is a strong candidate for publication following minor clarifications regarding scalability and data availability

Comment 3(1): Scalability Under Extreme Traffic Conditions

Reviewer Comment:

"The throughput is reported at 210 blocks per second. The authors should include a brief discussion on how the system would behave under extreme traffic conditions, such as a volumetric DDoS attack, where log generation might exceed this rate."

Response:

We would like to acknowledge this insightful comment by the reviewer. We agree that practical deployment considerations under high-volume attack conditions warrant explicit discussion.

The proposed audit ledger supports about 210 blocks/second with average latency of about 5 milliseconds for each block. Overhead is mainly due to SHA-256 hash calculations, metadata serialization, and operations related to append-only ledger management; not due to consensus, mining, peer-to-peer communication, or smart-contract execution, as the design of the suggested system is deliberately aimed at being lightweight, blockchain-inspired single-node hash chain audit ledger. For volumetric DDoS attacks, the rate of alerts generated can briefly surpass the logging capabilities of the ledger. Such instances may impose logging backpressure; however, the proposed architecture distinguishes between detection capability and audit logging capability. This allows for potential improvement regarding scalability in future implementations via asynchronous event buffering, dedicated ledger writers, batching of blocks, ledger partitioning across multiple nodes, and use of stream processing frameworks, such as Apache Kafka or Apache Flink. To address this concern, a new discussion has been incorporated into the manuscript in discussion section also.

TRACK CHANGE MANUSCRIPT

Section:

Lightweight Blockchain-Inspired Audit Ledger - (Line No 795-812)

Discussion Section = (Line No –1009-1021)

Comment 3 (2): Encrypted Traffic Handling

Reviewer Comment:

"Given that the majority of modern network traffic is encrypted, it would be beneficial for the authors to clarify if the H-IDS operates on packet headers or if it requires decrypted payloads."

Response:

We thank the reviewer for highlighting this important deployment consideration.

The proposed H-IDS is based on statistical and metadata-based features extracted from the network traffic at the flow level. The NSL-KDD and CICIDS2017 dataset used in this study includes flow-duration, packets, bytes, inter-arrival, TCP flag frequency, connection metadata, and other relevant features. These features are extracted from the headers of the packets and the flow logs and are independent of application-layer payloads. Therefore, the approach proposed in this research work will be intrinsically compatible with any TLS/SSL encryption environment since the encrypted behavior at the transport layer and network layer can still be captured using the proposed approach. In addition, the application-layer attack specially crafted to behave as benign encrypted flows may require additional techniques including TLS fingerprint analysis, behavioral profiling, or sequence-based anomaly detection. The manuscript has been revised accordingly. To address this concern, a new discussion has been incorporated into the manuscript in discussion section also.

TRACK CHANGE MANUSCRIPT

Section:

Experimental Setup and Implementation (Line no- 503-512)

Discussion Section = (Line No –1030-1042)

Comment 3(3): Reproducibility and Open Science Compliance

Reviewer Comment:

"To align with PLOS, ONE's commitment to open science, the authors are encouraged to provide a link to a public repository containing the custom weighting logic and the ledger implementation."

Response:

We fully agree with the reviewer regarding the importance of reproducibility and open science.

To support independent verification of all reported experiments, all the reported experiments, the entire implementation of the H-IDS architecture has been made freely available for use by all users. This includes leakage-proof pre-processing, PCA and SMOTE processing modules, supervised and unsupervised models training scripts, adaptive ensemble weighting process implementation, SHA-256 hash-chain inspired audit ledger, notebooks, configuration files, and other reproducibility resources.

The repository is publicly accessible at:

Public GitHub repository (https://github.com/rubaamff/Hybrid-IDS-Blockchain) and the DOI-registered Zenodo archive (https://doi.org/10.5281/zenodo.18009378), ensuring full reproducibility, transparency, and long-term accessibility in accordance with PLOS ONE open science policy.

The manuscript has been revised accordingly.

TRACK CHANGE MANUSCRIPT

Section: Leakage-Proof Preprocessing Pipeline

Line No -269-274

Section: Data Availability Statement ( After Conclusion )

Line No -1089-1096

We sincerely thank the Academic Editor and Reviewer #2 for their constructive comments, which have helped improve the clarity, transparency, reproducibility, consistency, and practical relevance of the manuscript. We believe that the revised manuscript fully addresses all remaining concerns and respectfully submit it for further consideration.

Sincerely,

Shailendra Mishra

(On behalf of all the authors)

Attachment Submitted filename: Point to Point EDITOR and Reviewer 2 Responce.docx

# Peer review: Decision Letter 5

**DOI:** 10.1371/journal.pone.0356878.r011

**Authors:** Sohail Saif

10 Aug 2026

<p>A Lightweight Blockchain-Inspired Hybrid Intrusion Detection System with Ensemble Learning for Tamper-Proof Auditing

PONE-D-25-59640R5

Dear Dr. Mishra,

We’re pleased to inform you that your manuscript has been judged scientifically suitable for publication and will be formally accepted for publication once it meets all outstanding technical requirements.

Within one week, you’ll receive an e-mail detailing the required amendments. When these have been addressed, you’ll receive a formal acceptance letter and your manuscript will be scheduled for publication.

An invoice will be generated when your article is formally accepted. Please note, if your institution has a publishing partnership with PLOS and your article meets the relevant criteria, all or part of your publication costs will be covered. Please make sure your user information is up-to-date by logging into Editorial Manager at Editorial Manager® and clicking the ‘Update My Information' link at the top of the page. For questions related to billing, please contact billing support.

If your institution or institutions have a press office, please notify them about your upcoming paper to help maximize its impact. If they’ll be preparing press materials, please inform our press team as soon as possible -- no later than 48 hours after receiving the formal acceptance. Your manuscript will remain under strict press embargo until 2 pm Eastern Time on the date of publication. For more information, please contact onepress@plos.org.

Kind regards,

Sohail Saif, Ph.D

Academic Editor

PLOS One

Additional Editor Comments (optional):

Reviewers' comments:

Reviewer's Responses to Questions

**Comments to the Author**

Reviewer #1: All comments have been addressed

**********

2. Is the manuscript technically sound, and do the data support the conclusions?

Reviewer #1: Yes

**********

3. Has the statistical analysis been performed appropriately and rigorously?

Reviewer #1: Yes

**********

4. Have the authors made all data underlying the findings in their manuscript fully available?

The PLOS Data policy

Reviewer #1: Yes

**********

5. Is the manuscript presented in an intelligible fashion and written in standard English?

Reviewer #1: Yes

**********

Reviewer #1: The revised manuscript has been substantially improved and addresses the concerns raised during the previous review rounds. The authors have provided clear and satisfactory responses to the comments regarding scalability under high-volume attack scenarios, compatibility with encrypted traffic, and reproducibility through public release of the implementation and experimental resources. The additional discussions improve both the practical relevance and transparency of the work. PONE-D-25-59640_R5.pdf

The proposed hybrid intrusion detection system combines supervised and unsupervised learning with a lightweight blockchain-inspired audit mechanism in a well-motivated and technically sound framework. The experimental methodology follows appropriate machine learning practices, including leakage-free preprocessing, cross-validation, statistical significance testing, and evaluation on two widely used benchmark datasets. The reported performance demonstrates strong detection capability while maintaining a lightweight tamper-evident auditing mechanism. PONE-D-25-59640_R5.pdf

The authors have also strengthened the manuscript by clarifying that the audit ledger is intentionally implemented as a lightweight single-node hash-chain mechanism rather than a decentralized blockchain, discussing its scalability limitations and future extensions, and explaining that the proposed IDS operates on flow-level statistical and metadata features, making it applicable to encrypted network traffic without requiring payload inspection. Furthermore, the availability of the complete source code and datasets significantly enhances reproducibility and aligns with the journal’s open science requirements. PONE-D-25-59640_R5.pdf

Overall, I believe the manuscript is technically rigorous, clearly presented, and provides a meaningful contribution to the field of intrusion detection systems by integrating ensemble learning with tamper-evident auditing. I have no further major concerns and recommend the manuscript for publication in its current form.

**********

what does this mean?). If published, this will include your full peer review and any attached files.

If you choose “no”, your identity will remain anonymous but your review may still be made public.

**Do you want your identity to be public for this peer review?**  For information about this choice, including consent withdrawal, please see our Privacy Policy

Reviewer #1: **Yes:**  Wenke Du

**********

# Editor report: Acceptance letter

**DOI:** 10.1371/journal.pone.0356878.r012

**Authors:** Sohail Saif

PONE-D-25-59640R5

PLOS One

Dear Dr. Mishra,

I'm pleased to inform you that your manuscript has been deemed suitable for publication in PLOS One. Congratulations! Your manuscript is now being handed over to our production team.

At this stage, our production department will prepare your paper for publication. This includes ensuring the following:

* All references, tables, and figures are properly cited

* All relevant supporting information is included in the manuscript submission,

* There are no issues that prevent the paper from being properly typeset

You will receive further instructions from the production team, including instructions on how to review your proof when it is ready. Please keep in mind that we are working through a large volume of accepted articles, so please give us a few days to review your paper and let you know the next and final steps.

Lastly, if your institution or institutions have a press office, please let them know about your upcoming paper now to help maximize its impact. If they'll be preparing press materials, please inform our press team within the next 48 hours. Your manuscript will remain under strict press embargo until 2 pm Eastern Time on the date of publication. For more information, please contact onepress@plos.org.

You will receive an invoice from PLOS for your publication fee after your manuscript has reached the completed accept phase. If you receive an email requesting payment before acceptance or for any other service, this may be a phishing scheme. Learn how to identify phishing emails and protect your accounts at https://explore.plos.org/phishing.

If we can help with anything else, please email us at customercare@plos.org.

Thank you for submitting your work to PLOS One and supporting open access.

Kind regards,

PLOS One Editorial Office Staff

on behalf of

Dr. Sohail Saif

Academic Editor

PLOS One
